# Associations Between the Digital Clock Drawing Test and Brain Volume: Large Community-Based Prospective Cohort (Framingham Heart Study)

**DOI:** 10.2196/34513

**Published:** 2022-04-15

**Authors:** Jing Yuan, Rhoda Au, Cody Karjadi, Ting Fang Ang, Sherral Devine, Sanford Auerbach, Charles DeCarli, David J Libon, Jesse Mez, Honghuang Lin

**Affiliations:** 1 Department of Neurology Peking Union Medical College Hospital Peking Union Medical College, Chinese Academy of Medical Sciences Beijing China; 2 Department of Anatomy and Neurobiology Boston University School of Medicine Boston University Boston, MA United States; 3 Framingham Heart Study Boston University School of Medicine Boston University Boston, MA United States; 4 Department of Neurology Boston University School of Medicine Boston University Boston, MA United States; 5 Department of Epidemiology Boston University School of Public Health Boston University Boston, MA United States; 6 Slone Epidemiology Center Boston University School of Medicine Boston University Boston, MA United States; 7 Alzheimer’s Disease Research Center Boston University Boston, MA United States; 8 Department of Neurology and Center for Neuroscience University of California, Davis Sacramento, CA United States; 9 Department of Geriatrics and Gerontology and Department of Psychology New Jersey Institute for Successful Aging Rowan University, School of Osteopathic Medicine Stratford, NJ United States; 10 Division of Clinical Informatics Department of Medicine University of Massachusetts Chan Medical School Worcester, MA United States

**Keywords:** Clock Drawing Test, digital, neuropsychological test, cognitive, technology, Boston Process Approach, neurology, Framingham Heart Study, dementia, Alzheimer

## Abstract

**Background:**

The digital Clock Drawing Test (dCDT) has been recently used as a more objective tool to assess cognition. However, the association between digitally obtained clock drawing features and structural neuroimaging measures has not been assessed in large population-based studies.

**Objective:**

We aimed to investigate the association between dCDT features and brain volume.

**Methods:**

This study included participants from the Framingham Heart Study who had both a dCDT and magnetic resonance imaging (MRI) scan, and were free of dementia or stroke. Linear regression models were used to assess the association between 18 dCDT composite scores (derived from 105 dCDT raw features) and brain MRI measures, including total cerebral brain volume (TCBV), cerebral white matter volume, cerebral gray matter volume, hippocampal volume, and white matter hyperintensity (WMH) volume. Classification models were also built from clinical risk factors, dCDT composite scores, and MRI measures to distinguish people with mild cognitive impairment (MCI) from those whose cognition was intact.

**Results:**

A total of 1656 participants were included in this study (mean age 61 years, SD 13 years; 50.9% women), with 23 participants diagnosed with MCI. All dCDT composite scores were associated with TCBV after adjusting for multiple testing (*P* value <.05/18). Eleven dCDT composite scores were associated with cerebral white matter volume, but only 1 dCDT composite score was associated with cerebral gray matter volume. None of the dCDT composite scores was associated with hippocampal volume or WMH volume. The classification model for differentiating MCI and normal cognition participants, which incorporated age, sex, education, MRI measures, and dCDT composite scores, showed an area under the curve of 0.897.

**Conclusions:**

dCDT composite scores were significantly associated with multiple brain MRI measures in a large community-based cohort. The dCDT has the potential to be used as a cognitive assessment tool in the clinical diagnosis of MCI.

## Introduction

The Clock Drawing Test (CDT) is a widely used assessment tool able to screen for impairment associated with mild cognitive impairment (MCI) [1] and dementia [2-5]. The most common instruction for clock drawing asks participants to draw the face of a clock, put in all of the numbers, and set the hands for 10 past 11. This is followed by asking participants to copy a model of a clock [3,6]. Multiple cognitive domains are involved in completion of the test, including graphomotor ability, attention, syntactic comprehension, visual and semantic memory, executive function, and visuoconstructional ability [6]. Previous studies found that CDT performance evaluated by various manual scoring systems was correlated with a variety of cortical and subcortical areas on magnetic resonance imaging (MRI), without showing consistent and specific brain localization [7-9].

A significant innovation is the introduction of a digital CDT (dCDT), which was jointly developed by the Massachusetts Institute of Technology and Lahey Hospital & Medical Center, in collaboration with the Clock Sketch Consortium [10-13]. In addition to retaining the classic features of the traditional CDT, including easy, inexpensive, noninvasive administration, the dCDT captures more than 100 latencies and graphomotor features for subtle cognitive changes, which would be difficult or impossible without this technology [12-14]. A recent study reported that the DCTClock test (a commercial version of the dCDT) showed excellent discrimination between diagnostic groups of normal cognition (NC) and MCI or early Alzheimer dementia [14]. The test was also associated with amyloid β and tau burden in positron emission tomography scans among NC participants [14].

In our prior study [15], we validated the psychometric characteristics of the dCDT against standard paper-pencil neuropsychological (NP) tests in the community-based Framingham Heart Study (FHS) [15]. The composite scores derived from dCDT features were significantly associated with both NP test results and mild cognitive impairment [15]. Nonetheless, it is unclear whether dCDT composite scores are associated with MRI measures of atrophy, which have been used as important markers for cognitive impairment and cerebral small vessel disease injuries [16-20].

The objectives of this study were to investigate the associations between dCDT features and MRI measures in the FHS, and to assess the diagnostic potential of dCDT features as a surrogate for standard NP tests in the differentiation of MCI from NC that is validated against MRI measures of neurodegeneration.

## Methods

### Study Sample

The FHS is a community-based prospective cohort study that was established in 1948. Three generations of participants have been enrolled and followed up every 2 to 8 years. Details on the FHS cohorts can be found in previous publications [21-23]. As part of an ancillary study, participants were invited to undergo NP assessment and brain MRI scans regularly. The dCDT has been administered as part of the NP assessment along with other NP tests since 2011. This study included participants who completed at least one dCDT and had a contemporary MRI within 6 months (98.2% had MRI scans on the same day as the dCDT). Participants with prevalent dementia and stroke were excluded (n=84). We further excluded participants who were flagged as having possible cognitive impairment, but were not reviewed by an expert panel (n=139).

### Ethics Approval

The Boston University Medical Campus Institutional Review Board approved the study procedures and protocols. Written informed consent was obtained from all participants. All the data used for this study could be requested through the FHS research website [24].

### Standard NP Tests and the dCDT

In the FHS, standard NP tests were administered and scored to produce traditional quantitative measures of cognitive performance [25]. These tests included the Wechsler Memory Scale [26] Logical Memory—Immediate Recall, Delayed Recall, and Recognition; Visual Reproduction—Immediate Recall, Delayed Recall, and Recognition; and Paired Associate Learning—Immediate Recall, Delayed Recall, and Recognition; the Wechsler Adult Intelligence Scale [27] Digit Span—Forward and Backward and Similarities; the Boston Naming Test—30 item version [28]; the Trail Making Test A and B [29]; the Hooper Visual Organization Test [30]; and Verbal Fluency (FAS & FAS-Animal) [31,32]. These NP tests measure multiple cognitive domains of verbal memory, visual memory, attention and concentration, executive function, abstract reasoning, language, and visuoperceptual organization. For all tests, higher scores indicate better performance with the exception of the Trail Making Test A and B, where a shorter completion time indicates better cognitive performance.

Since October 2011, FHS participants have been administered the dCDT with a digital pen during their regular NP test visit by trained examiners [10-13]. The digital pen measures the pen position 80 times per second at a spatial resolution of ±0.002 inches [10]. After the dCDT test, the pen was connected to a computer with preinstalled software that automatically extracted the drawing information and classified each pen stroke and associated latencies. An external rater manually examined each drawing to ensure appropriate classification of strokes using a user-friendly drag-and-drop interface to correct any classification error. A total of 105 dCDT features were derived and used from the entire drawing process, which captured the strokes, latencies, and spatial relationships as the measures of drawing efficiency, simple motor functioning, information processing, and spatial reasoning (Multimedia Appendix 1). As reported in our previous study [15], we further derived composite scores based on dCDT features significantly associated with 18 NP tests, which represented a weighted combination of dCDT features that were previously associated with a specific NP test.

### Acquisition and Measurement of MRI Variables

The MRI protocol in the FHS has been described previously [33]. Briefly, participants were imaged using a Siemens 1.5T field strength machine (Siemens Medical) and a 3-dimensional T1-weighted coronal spoiled gradient-recalled echo sequence. All images were transferred to and processed by the University of California Davis Medical Center for centralized processing. Segmentation of brain structural MRI was performed by semiautomated procedures, and the complete information can be obtained elsewhere [33]. In brief, gray matter, white matter, and cerebrospinal fluid segmentation were performed using an expectation-maximization algorithm after skull stripping and intensity inhomogeneity correction. The hippocampus was segmented by a multi-atlas hippocampal segmentation algorithm [34]. White matter hyperintensity (WMH) was segmented on a combination of FLAIR and 3D T1 images using a modified Bayesian probability structure [35]. Total cerebral cranial volume (TCV) was determined by outlining the intracranial vault lying above the tentorium and was used for correcting the head size. The primary MRI measure was total cerebral brain volume (TCBV). Secondary measures were cerebral white matter volume, cerebral gray matter volume, hippocampal volume, and WMH volume. All MRI measures were corrected for head size by calculating the percentage of these volumes over the TCV. The percent of WMH/TCV was log-transformed for normality. The WMH volume was used both as a continuous variable and as a dichotomous variable (large WMH volume versus no or minimal WMH volume), similar to a previous study [36]. A large WMH volume (WMH-Large) was defined as a volume more than one standard deviation higher than the age-specific mean value.

### Case Ascertainment of MCI

The detailed cognitive ascertainment procedure and quality control in the FHS have been described previously [37,38]. Briefly, participants received NP assessments on average every 4 to 5 years, in addition to their regular research center examinations. For participants with possible cognitive impairment, more frequent NP tests were conducted on average every 1 to 2 years, and neurological examinations were performed on a subset of these participants. A clinical review was triggered when there was an indication of a potential cognitive impairment and/or decline, and was conducted by a panel consisting of at least one neurologist and one neuropsychologist. The review panel determined the MCI diagnosis, which required evidence of a decline in cognitive performance in one or more cognitive domains, no records indicating functional decline, and not meeting the criteria for dementia. Although the Clinical Dementia Rating (CDR) scale [39] was not formally applied, the review panel used the CDR scoring scale (0-3) to quantify the severity of impairment; all MCI cases were given a rating of 0.5.

### Statistical Analysis

The dCDT composite scores were derived by linear regression models based on dCDT features significantly associated with 18 NP tests, which represented a weighted combination of dCDT features that were previously associated with a specific NP test [15]. The scores and all MRI measures were normalized with a mean of 0 and standard deviation of 1. Linear regression models were used to assess the associations of dCDT composite scores with MRI measures, adjusting for age, sex, and education. The same models were also used to test the association between individual dCDT features and MRI measures. In the sensitivity analysis, the models were additionally adjusted for vascular risk factors, including hypertension, diabetes, smoking, and prevalent atrial fibrillation. Bonferroni correction was used to adjust for multiple testing in the linear regression models. Significant associations were claimed if *P*<.05/N, where N was the number of tests performed.

Logistic regression models were also constructed to classify MCI using 4 different sets of predictors. The analysis was restricted to participants who were 65 years or older at the time of NP testing (only 1 case was diagnosed before 65 years). Model 1 included only age, sex, and education. Model 2 included age, sex, education, and MRI measures (TCBV, cerebral white matter volume, cerebral gray matter volume, and hippocampal volume). Model 3 included age, sex, education, and dCDT composite scores. Model 4 included age, sex, education, MRI measures, and dCDT composite scores. Model performance was assessed by the area under the curve (AUC). All statistical analyses were performed using R software version 4.0.3 (R Core Team) [40].

## Results

As shown in Table 1, our study sample included 1656 participants (mean age 61 years, SD 13 years; 50.9% women; 42.9% received college-level education or higher) who were free of dementia or stroke when NP tests were conducted. Among them, 23 participants were diagnosed with MCI at the time of or before their NP tests but had not progressed to the threshold of clinical dementia diagnosis. As expected, participants with MCI were generally older and had worse cognitive performance, smaller cerebral volume measures, and larger WMH volume on MRI than those in the NC group.

**Table 1:** Clinical characteristics of the study sample.

Variable		Total (N=1656)	MCI^a^ (n=23)	NC^b^ (n=1633)
Age (years), mean (SD)		61 (13)	78 (7)	61 (13)
Women, n (%)		843 (50.9)	14 (60.9)	829 (50.8)
**Education, n (%)**				
	No high school	187 (11.3)	3 (13.0)	184 (11.3)
	High school	319 (19.3)	6 (26.1)	313 (19.2)
	Some college	440 (26.6)	3 (13.0)	437 (26.8)
	College and higher	710 (42.9)	11 (47.8)	699 (42.8)
**MRI^c^ measures^d^, mean (SD)**				
	Total cerebral cranial volume (cm^3^)	1251 (129)	1252 (104)	1251 (129)
	Total cerebral brain volume (%)	77.0 (2.9)	72.8 (2.5)	77.0 (2.9)
	Cerebral white matter volume (%)	36.3 (2.4)	34.3 (2.7)	36.3 (2.4)
	Cerebral gray matter volume (%)	40.4 (2.1)	37.4 (2.2)	40.4 (2.1)
	Hippocampal volume (%)	0.54 (0.05)	0.50 (0.05)	0.54 (0.05)
	Log (white matter hyperintensity volume) (%)	−2.33 (1.48)	−0.59 (1.38)	−2.35 (1.47)
**Neuropsychological test scores, mean (SD)**				
	LMi^e^	13 (3)	10 (4)	13 (3)
	LMd^e^	12 (4)	8 (4)	12 (4)
	LMr^e^	10 (1)	9 (2)	10 (1)
	VRi^f^	9 (3)	5 (2)	9 (3)
	VRd^f^	8 (3)	3 (3)	8 (3)
	VRr^f^	3 (1)	2 (1)	3 (1)
	PASi^g^	15 (3)	11 (3)	15 (3)
	PASd^g^	9 (1)	7 (2)	9 (1)
	PASr^g^	10 (1)	9 (2)	10 (1)
	DSf^h^	7 (1)	6 (1)	7 (1)
	DSb^h^	5 (1)	4 (1)	5 (1)
	Trails A^i^ (s)	31 (13)	44 (14)	31 (13)
	Trails B^i^ (s)	83 (65)	219 (159)	82 (61)
	Similarities	17 (3)	14 (5)	17 (3)
	Hooper Visual Organization Test	26 (3)	23 (4)	26 (3)
	Boston Naming Test—30 item version	26 (7)	24 (7)	26 (7)
	FAS^j^	41 (12)	32 (12)	41 (12)
	FAS-Animal^j^	19 (6)	15 (5)	19 (6)

^a^MCI: mild cognitive impairment.

^b^NC: normal cognition.

^c^MRI: magnetic resonance imaging.

^d^All MRI measures were corrected for head size by calculating the percentage of the volumes over the total cerebral cranial volume above the tentorium. The percentage of white matter hyperintensity volume/total cerebral cranial volume was log transformed.

^e^Wechsler Memory Scale Logical Memory—Immediate Recall (LMi), Delayed Recall (LMd), and Recognition (LMr).

^f^Visual Reproduction—Immediate Recall (VRi), Delayed Recall (VRd), and Recognition (VRr).

^g^Paired Associate Learning—Immediate Recall (PASi), Delayed Recall (PASd), and Recognition (PASr).

^h^Wechsler Adult Intelligence Scale Digit Span—Forward (DSf) and Backward (DSb).

^i^Trail Making Test A (Trails A) and B (Trails B).

^j^Verbal fluency test (FAS and FAS-Animal).

As shown in Table 2, all dCDT composite scores (n=18) were significantly associated with TCBV after adjusting for multiple testing. Better dCDT performance was associated with larger TCBV. The effect size of composite scores for visual memory and visuoperceptual organization tended to be slightly higher than other dCDT composite scores.

**Table 2:** Association between digital Clock Drawing Test composite scores and total cerebral brain volume.

dCDT^a^ composite score^b^	Effect size	Standard error	*P* value^c^
dCDT_LMi^d^	0.079	0.017	3.2×10^−6^
dCDT_LMd^d^	0.083	0.017	1.1×10^−6^
dCDT_LMr^d^	0.061	0.017	2.9×10^−4^
dCDT_VRi^e^	0.098	0.017	1.4×10^−8^
dCDT_VRd^e^	0.099	0.017	1.2×10^−8^
dCDT_VRr^e^	0.093	0.017	6.4×10^−8^
dCDT_PASi^f^	0.079	0.017	3.5×10^−6^
dCDT_PASd^f^	0.084	0.017	8.8×10^−7^
dCDT_PASr^f^	0.073	0.017	1.7×10^−5^
dCDT_DSf^g^	0.071	0.017	2.5×10^−5^
dCDT_DSb^g^	0.067	0.017	6.5×10^−5^
dCDT_Trails A^h^	−0.074	0.017	1.2×10^−5^
dCDT_Trails B^h^	−0.080	0.017	3.0×10^−6^
dCDT_SIM^i^	0.084	0.017	9.4×10^−7^
dCDT_HVOT^j^	0.094	0.017	6.1×10^−8^
dCDT_BNT30^k^	0.075	0.017	9.9×10^−6^
dCDT_FAS^l^	0.083	0.017	1.5×10^−6^
dCDT_FAS-Animal^l^	0.062	0.017	2.2×10^−4^

^a^dCDT: digital Clock Drawing Test.

^b^The models were adjusted for age, sex, and education.

^c^Bonferroni correction was used to adjust for multiple testing, and significant associations were claimed if *P*<.05/18 (2.8×10^−3^), where 18 is the number of tests performed. All *P* values were significant.

^d^Wechsler Memory Scale Logical Memory—Immediate Recall (LMi), Delayed Recall (LMd), and Recognition (LMr).

^e^Visual Reproduction—Immediate Recall (VRi), Delayed Recall (VRd), and Recognition (VRr).

^f^Paired Associate Learning—Immediate Recall (PASi), Delayed Recall (PASd), and Recognition (PASr).

^g^Wechsler Adult Intelligence Scale Digit Span—Forward (DSf) and Backward (DSb).

^h^Trail Making Test A (Trails A) and B (Trails B).

^i^SIM: Similarities.

^j^HVOT: Hooper Visual Organization Test.

^k^BNT30: Boston Naming Test—30 item version.

^l^Verbal fluency test (FAS and FAS-Animal).

As shown in Table 3, cerebral white matter volume was significantly associated with 11 dCDT composite scores involving the comprehensive cognitive domains of verbal memory, visual memory, abstract reasoning, language, and visuoperceptual organization (all with *P*<2.8×10^−3^). In contrast, cerebral gray matter volume was only associated with the visual memory composite score (dCDT_Visual Reproduction—Immediate Recall, β=.059; *P*=2.6×10^−3^). Hippocampal volume was not significantly associated with any dCDT composite scores. As shown in Table 4, none of the dCDT composite scores was significantly associated with WMH volume.

**Table 3:** Association of digital Clock Drawing Test composite scores with cerebral white matter, gray matter, and hippocampal volumes.

dCDT^a^ composite score^b^	Cerebral white matter volume^c^				Cerebral gray matter volume^c^				Hippocampal volume^c^			
	Effect size	Standard error	*P* value^d^	Effect size		Standard error	*P* value^d^	Effect size		Standard error	*P* value^d^	
dCDT_LMi^e^	0.071	0.023	1.9×10^−3f^	0.042		0.019	2.8×10^−2^	0.033		0.025	2.0×10^−1^	
dCDT_LMd^e^	0.075	0.023	1.1×10^−3f^	0.045		0.019	2.0×10^−2^	0.038		0.025	1.3×10^−1^	
dCDT_LMr^e^	0.052	0.022	2.0×10^−2^	0.034		0.019	7.1×10^−2^	0.019		0.025	4.6×10^−1^	
dCDT_VRi^g^	0.083	0.023	4.2×10^−4f^	0.059		0.020	2.6×10^−3f^	0.054		0.026	3.8×10^−2^	
dCDT_VRd^g^	0.084	0.023	3.0×10^−4f^	0.058		0.020	3.1×10^−3^	0.054		0.026	3.9×10^−2^	
dCDT_VRr^g^	0.080	0.023	5.6×10^−4f^	0.054		0.020	5.5×10^−3^	0.052		0.026	4.3×10^−2^	
dCDT_PASi^h^	0.070	0.023	2.3×10^−3f^	0.044		0.019	2.2×10^−2^	0.035		0.025	1.7×10^−1^	
dCDT_PASd^h^	0.078	0.023	7.3×10^−4f^	0.044		0.019	2.4×10^−2^	0.041		0.026	1.1×10^−1^	
dCDT_PASr^h^	0.067	0.023	3.2×10^−3^	0.036		0.019	6.2×10^−2^	0.047		0.025	6.5×10^−2^	
dCDT_DSf^i^	0.063	0.023	5.7×10^−3^	0.041		0.019	3.4×10^−2^	0.026		0.025	3.1×10^−1^	
dCDT_DSb^i^	0.059	0.023	9.0×10^−3^	0.037		0.019	5.0×10^−2^	0.023		0.025	3.7×10^−1^	
dCDT_Trails A^j^	−0.063	0.023	6.0×10^−3^	−0.045		0.019	1.8×10^−2^	−0.046		0.025	7.0×10^−2^	
dCDT_Trails B^j^	−0.069	0.023	2.9×10^−3^	−0.047		0.019	1.4×10^−2^	−0.047		0.025	6.7×10^−2^	
dCDT_SIM^k^	0.071	0.023	2.0×10^−3f^	0.049		0.019	1.2×10^−2^	0.034		0.026	1.8×10^−1^	
dCDT_HVOT^l^	0.083	0.023	4.0×10^−4f^	0.052		0.020	8.0×10^−3^	0.048		0.026	6.4×10^−2^	
dCDT_BNT30^m^	0.071	0.023	1.9×10^−3f^	0.036		0.019	5.9×10^−2^	0.028		0.025	2.7×10^−1^	
dCDT_FAS^n^	0.071	0.023	2.1×10^−3f^	0.047		0.019	1.5×10^−2^	0.040		0.026	1.2×10^−1^	
dCDT_FAS-Animal^n^	0.054	0.023	1.7×10^−2^	0.034		0.019	7.1×10^−2^	0.021		0.025	4.0×10^−1^	

^a^dCDT: digital Clock Drawing Test.

^b^The models were adjusted for age, sex, and education.

^c^All magnetic resonance imaging measures were the percentage of the volumes over the total cerebral cranial volume above the tentorium.

^d^Bonferroni correction was used to adjust for multiple testing, and significant associations were claimed if *P*<.05/18 (2.8×10^−3^), where 18 is the number of tests performed.

^e^Wechsler Memory Scale Logical Memory—Immediate Recall (LMi), Delayed Recall (LMd), and Recognition (LMr).

^f^Significant.

^g^Visual Reproduction—Immediate Recall (VRi), Delayed Recall (VRd), and Recognition (VRr).

^h^Paired Associate Learning—Immediate Recall (PASi), Delayed Recall (PASd), and Recognition (PASr).

^i^Wechsler Adult Intelligence Scale Digit Span—Forward (DSf) and Backward (DSb).

^j^Trail Making Test A (Trails A) and B (Trails B).

^k^SIM: Similarities.

^l^HVOT: Hooper Visual Organization Test.

^m^BNT30: Boston Naming Test—30 item version.

^n^Verbal fluency test (FAS and FAS-Animal).

**Table 4:** Association between digital Clock Drawing Test composite scores and white matter hyperintensity volume.

dCDT^a^ composite score^b^	White matter hyperintensity volume^c^				White matter hyperintensity-Large^d^		
	Effect size	Standard error	*P* value^e^	Effect size		Standard error	*P* value^e^
dCDT_LMi^f^	0.040	0.018	3.0×10^−2^	−0.120		0.073	1.1×10^−1^
dCDT_LMd^f^	−0.041	0.018	2.5×10^−2^	−0.130		0.073	8.2×10^−2^
dCDT_LMr^f^	−0.035	0.018	5.0×10^−2^	−0.110		0.071	1.3×10^−1^
dCDT_VRi^g^	−0.042	0.019	2.3×10^−2^	−0.150		0.074	3.7×10^−2^
dCDT_VRd^g^	−0.043	0.019	2.0×10^−2^	−0.150		0.074	3.7×10^−2^
dCDT_VRr^g^	−0.043	0.019	2.2×10^−2^	−0.160		0.074	2.9×10^−2^
dCDT_PASi^h^	−0.036	0.018	5.0×10^−2^	−0.100		0.073	1.5×10^−1^
dCDT_PASd^h^	−0.040	0.018	3.0×10^−2^	−0.130		0.073	8.0×10^−2^
dCDT_PASr^h^	−0.020	0.018	2.7×10^−1^	−0.035		0.073	6.3×10^−1^
dCDT_DSf^i^	−0.042	0.018	2.2×10^−2^	−0.160		0.072	3.0×10^−2^
dCDT_DSb^i^	−0.036	0.018	4.7×10^−2^	−0.100		0.072	1.5×10^−1^
dCDT_Trails A^j^	0.037	0.018	4.4×10^−2^	0.160		0.073	3.1×10^−2^
dCDT_Trails B^j^	0.039	0.018	3.4×10^−2^	0.150		0.074	3.6×10^−2^
dCDT_SIM^k^	−0.039	0.018	3.5×10^−2^	−0.130		0.073	6.6×10^−2^
dCDT_HVOT^l^	−0.040	0.019	3.4×10^−2^	−0.140		0.074	5.7×10^−2^
dCDT_BNT30^m^	−0.037	0.018	4.5×10^−2^	−0.120		0.073	9.8×10^−2^
dCDT_FAS^n^	−0.041	0.018	2.5×10^−2^	−0.160		0.074	3.2×10^−2^
dCDT_FAS-Animal^n^	−0.035	0.018	4.8×10^−2^	−0.110		0.071	1.3×10^−1^

^a^dCDT: digital Clock Drawing Test.

^b^The models were adjusted for age, sex, and education.

^c^The white matter hyperintensity volume was the percentage over the total cerebral cranial volume above the tentorium and was log transformed.

^d^The large white matter hyperintensity volume was defined as that more than one standard deviation higher than the age-specific mean value.

^e^Bonferroni correction was used to adjust for multiple testing, and significant associations were claimed if *P*<.05/18 (2.8×10^−3^), where 18 is the number of tests performed.

^f^Wechsler Memory Scale Logical Memory—Immediate Recall (LMi), Delayed Recall (LMd), and Recognition (LMr).

^g^Visual Reproduction—Immediate Recall (VRi), Delayed Recall (VRd), and Recognition (VRr).

^h^Paired Associate Learning—Immediate Recall (PASi), Delayed Recall (PASd), and Recognition (PASr).

^i^Wechsler Adult Intelligence Scale Digit Span—Forward (DSf) and Backward (DSb).

^j^Trail Making Test A (Trails A) and B (Trails B).

^k^SIM: Similarities.

^l^HVOT: Hooper Visual Organization Test.

^m^BNT30: Boston Naming Test—30 item version.

^n^Verbal fluency test (FAS and FAS-Animal).

Further detailed analysis for cortical gray matter and specific brain areas (frontal, parietal, temporal, and occipital cortical gray matter volumes) is shown in Multimedia Appendix 2. We found that cortical gray matter volume was associated with visual memory composite scores (*P*=1.4×10^−3^ for dCDT_Visual Reproduction—Immediate Recall and 1.7×10^−3^ for dCDT_Visual Reproduction—Delayed Recall), which was consistent with the results for cerebral gray matter volume shown in Table 3. For specific cortical regions, parietal and temporal cortical gray matter volumes were associated with dCDT composite scores, whereas no association was found for frontal and occipital cortical gray matter volumes. The results remained largely the same after additionally adjusting for vascular risk factors (Multimedia Appendix 3, Multimedia Appendix 4, Multimedia Appendix 5, and Multimedia Appendix 6).

We also examined the associations between individual dCDT features and MRI measures. Among the 105 dCDT features, 17 of them were significantly associated with TCBV. However, only 2 features were associated with cerebral white matter volume and 4 features were associated with cerebral gray matter volume (Multimedia Appendix 7). The scattered associations suggest that, rather than individual features, the composite scores can better characterize the combinatory effects of multiple features.

**Figure 1:**
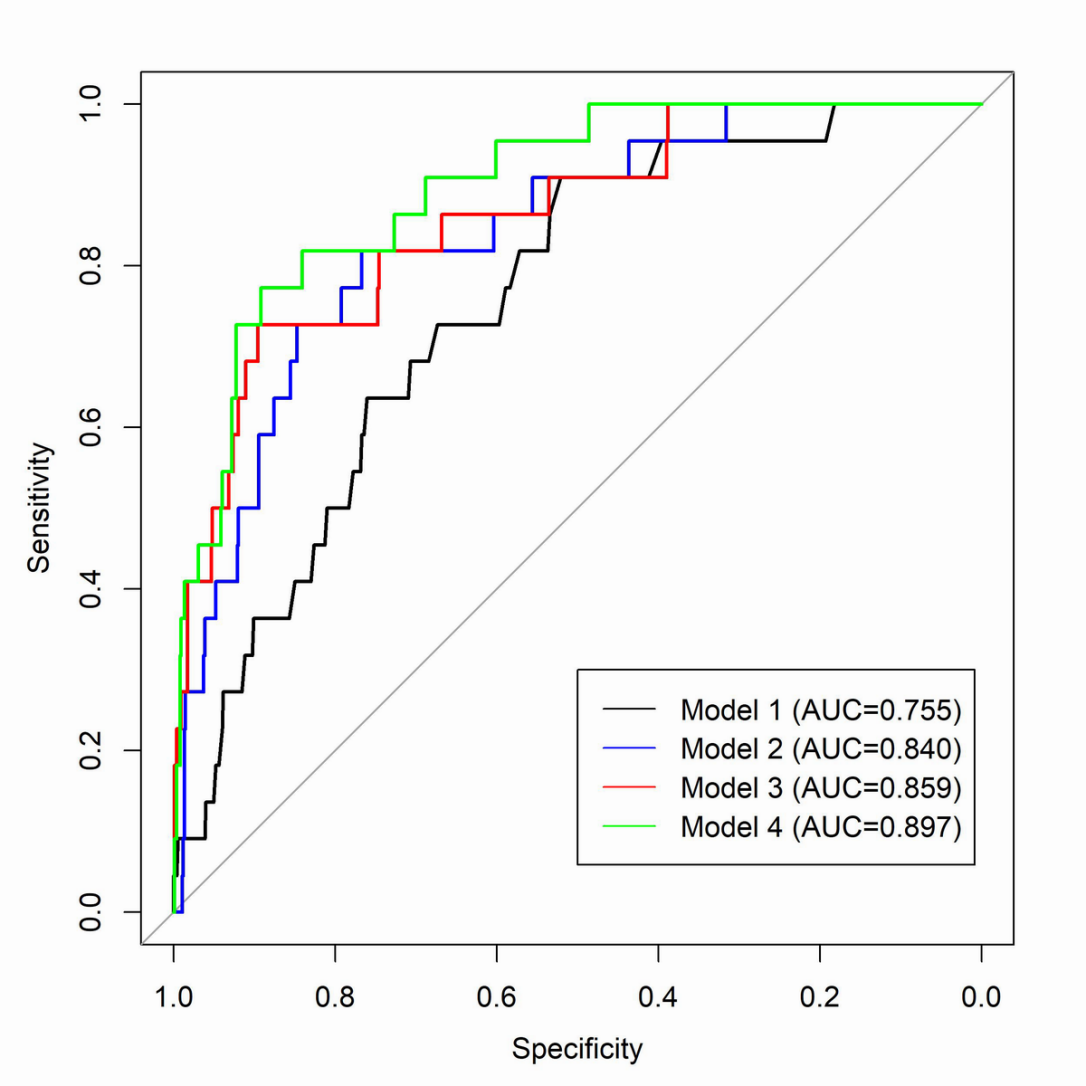
Performance of classification models to distinguish mild cognitive impairment from normal cognition. AUC: area under the curve.

We then built 4 classification models of MCI for participants over 65 years old, each with a different set of predictors. As shown in Figure 1, the AUCs were 0.755, 0.840, 0.859, and 0.897 for Models 1-4, respectively. Model 4 that incorporated traditional risk factors (age, sex, and education), MRI measures, and dCDT composite scores showed superior performance compared with models consisting of solely MRI or dCDT composite scores with traditional risk factors (Models 1-3).

## Discussion

In this study, we investigated the potential of the dCDT as a cognitive assessment tool by studying the association of dCDT features with brain MRI structural measures in more than 1600 participants. We observed significant associations between dCDT composite scores and multiple MRI measures, including TCBV, cerebral white matter volume, and cerebral gray matter volume. However, no association was observed between dCDT composite scores and hippocampal volume or WMH volume. The combination of dCDT composite scores with MRI measures and clinical risk factors reached an AUC of 0.897 to distinguish MCI from normal cognition. Given that standard NP tests are time burdensome and labor intensive, the dCDT might be used as an alternative tool to screen for MCI in a large population [12,13,41,42].

A previous study found that a smaller TCBV was associated with worse performance in Visual Reproduction—Immediate Recall, Visual Reproduction—Delayed Recall, Visual Reproduction—Recognition, the Trail Making Test A and B, and the Hooper Visual Organization Test [16]. Notably, this study found that global cerebrum atrophy was associated with several dCDT measures, suggesting a more extensive deficit in multiple cognitive domains, which might be used as an indicator of general “brain health.” Interestingly, dCDT performance tended to be better correlated with cerebral white matter volume, and parietal and temporal cortical gray matter volumes, suggesting that they may be the primary neuroanatomical basis for cognitive processes in the dCDT. While the cerebral cortex has long been considered as the major neuroanatomical basis of cognitive function, white matter is increasingly recognized as equally critical for cognition [43-45]. Cerebral white matter contains neural fibers that are the extensions of neurons into subcortical regions [46]. The neural network composed of cortical neurons and subcortical neural fibers is essential to maintain normal neural signal transmission and functional connectivity for cognitive processing [46]. Nonetheless, although there was a significant association between the dCDT and cerebral white matter volume, we did not find an association between dCDT performance and WMH volume. One possible reason is the exclusion of dementia patients in our sample, who generally have a larger WMH volume compared to that in individuals with normal cognition or MCI. Another reason may be that the dCDT performance was more closely associated with white matter atrophy rather than localized white matter injuries.

Several limitations of our study, however, merit consideration. First, the cross-sectional design could not reveal a temporal relationship between dCDT features and MRI measures. Future longitudinal analysis will investigate whether dCDT features could serve as early cognitive markers for predicting incident brain structural changes or incident MCI or dementia. Second, only a moderate number of MCI cases were observed in this study, and the number of dementia cases was too small to study. The diagnostic value of the dCDT could be further validated when more incident cases are observed during longer follow-up periods in the FHS. In addition, besides the Harvard Aging Brain Study, future studies with larger sample sizes of MCI and early Alzheimer dementia in more diverse populations (population-based or hospital-based samples) are warranted to further test the diagnostic performance before extensive application of the dCDT as a substitute for classic NP tests in classification and selection criteria for clinical research and clinical trials. Third, standard NP test performance was used during the dementia diagnosis review process, whereas dCDT composite scores were built from standard NP tests. However, dCDT performance was not directly used during the review process. Finally, yet importantly, FHS participants were mostly non-Hispanic White and native English speakers. Therefore, the generalizability of our findings to other ethnic populations is unknown.

Aside from these limitations, our study had several strengths. This is the first study to investigate the association between dCDT features and MRI measures in a population-based evaluation. All the NP tests and MRI measures were collected consistently with rigorous quality control. The MCI diagnosis was made by an experienced review panel, which capitalized on all available relevant data sources for consistent diagnosis. Further, given the current relatively widespread use of the paper-pencil clock drawing test as a cognitive screening tool in clinical practice, results from this study do support the potential use of a digital version in place of the paper-pencil version. This application may be of particular use in clinical settings where neuropsychological and neurological expertise is unavailable.

In summary, we assessed the psychometric characteristics of the dCDT with brain volume measures by MRI. In combination with age, sex, education, and MRI measures, the dCDT improved the classification performance of MCI similar to standard NP tests. Our results suggest that the dCDT may be used as a cost-effective and easy-to-administer tool by general practitioners to assess subtle cognitive changes occurring in MCI or early dementia stages and in underdeveloped countries or regions where medical resources are limited.

## Appendix

Multimedia Appendix 1 Raw feature names and descriptions.

Multimedia Appendix 2 Association between digital Clock Drawing Test composite scores and cortical gray matter volumes.

Multimedia Appendix 3 Association between digital Clock Drawing Test composite scores and total cerebral brain volume after additionally adjusting for vascular risk factors.

Multimedia Appendix 4 Association of digital Clock Drawing Test composite scores with cerebral white matter, gray matter, and hippocampal volumes after additionally adjusting for vascular risk factors.

Multimedia Appendix 5 Association between digital Clock Drawing Test composite scores and white matter hyperintensity volume after additionally adjusting for vascular risk factors.

Multimedia Appendix 6 Association between digital Clock Drawing Test composite scores and cortical gray matter after additionally adjusting for vascular risk factors.

Multimedia Appendix 7 Association between individual digital Clock Drawing Test features and magnetic resonance imaging measures.

## Abbreviations

AUC: area under the curve
CDR: Clinical Dementia Rating
CDT: Clock Drawing Test
dCDT: digital Clock Drawing Test
FHS: Framingham Heart Study
MCI: mild cognitive impairment
MRI: magnetic resonance imaging
NC: normal cognition
NP: neuropsychological
TCBV: total cerebral brain volume
TCV: total cerebral cranial volume
WMH: white matter hyperintensity
