# Supplementary material for: Associations Between the Digital Clock Drawing Test and Brain Volume: Large Community-Based Prospective Cohort (Framingham Heart Study)
Source: J Med Internet Res. 2022 Apr 15;24(4):e34513. doi: 10.2196/34513 (PMC9055470; doi:10.2196/34513)
Supplement: Multimedia Appendix 1 [file jmir_v24i4e34513_app1.docx]

**Multimedia Appendix 1.** Raw feature names and descriptions.

| No | Raw feature name | Short Description | Long Description |
| --- | --- | --- | --- |
| 1 | DCTScore | The DCTclock score | A number between 0 and 100 that represents a person's overall cognitive function as assessed by DCTclock. |
| 2 | COMDrawingEfficiency_s | Drawing Efficiency on command clock | The efficiency the patient demonstrated during the process of drawing the command clock. This considers metrics such as time spent relative to properties of the drawing produced including number of pen strokes, stroke length, and size of the drawing. |
| 3 | COPDrawingEfficiency_s | Drawing Efficiency on copy clock | The efficiency the patient demonstrated during the process of drawing the copy clock. This considers metrics such as time spent relative to properties of the drawing produced including number of pen strokes, stroke length, and size of the drawing. |
| 4 | COMInformationProcessing_s | Information Processing on command clock | The non-motor cognitive functions used during the process of drawing the command clock. This considers metrics such as absolute and relative duration of latencies, number of pauses, and relative time spent thinking versus actively drawing with pen on the paper. |
| 5 | COPInformationProcessing_s | Information Processing on copy clock | The non-motor cognitive functions used during the process of drawing the copy clock. This considers metrics such as absolute and relative duration of latencies, number of pauses, and relative time spent thinking versus actively drawing with pen on the paper. |
| 6 | COMSimpleMotor_s | Simple and Complex Motor on command clock | The graphomotor components involved in the process of drawing the command clock. This considers metrics such as pen stroke speeds and oscillatory motion and can be helpful in parsing out motor from non-motor cognitive functions. |
| 7 | COPSimpleMotor_s | Simple and Complex Motor on copy clock | The graphomotor components involved in the process of drawing the copy clock. This considers metrics such as pen stroke speeds and oscillatory motion and can be helpful in parsing out motor from non-motor cognitive functions. |
| 8 | COMSpatialReasoning_s | Spatial Reasoning on command clock | The spatial abilities demonstrated during the process of drawing the command clock. This considers metrics pertaining to the geometric properties of the drawing including the circularity of the clock circle, placement of clock components, and drawing placement on the page. |
| 9 | COPSpatialReasoning_s | Spatial Reasoning on copy clock | The spatial abilities demonstrated during the process of drawing the copy clock. This considers metrics pertaining to the geometric properties of the drawing including the circularity of the clock circle, placement of clock components, and drawing placement on the page. |
| 10 | COMDrawingEfficiency | Drawing Efficiency on command clock | The efficiency the patient demonstrated during the process of drawing the command clock. This considers metrics such as time spent relative to properties of the drawing produced including number of pen strokes, stroke length, and size of the drawing. |
| 11 | COPDrawingEfficiency | Drawing Efficiency on copy clock | The efficiency the patient demonstrated during the process of drawing the copy clock. This considers metrics such as time spent relative to properties of the drawing produced including number of pen strokes, stroke length, and size of the drawing. |
| 12 | COMInformationProcessing | Information Processing on command clock | The non-motor cognitive functions used during the process of drawing the command clock. This considers metrics such as absolute and relative duration of latencies, number of pauses, and relative time spent thinking versus actively drawing with pen on the paper. |
| 13 | COPInformationProcessing | Information Processing on copy clock | The non-motor cognitive functions used during the process of drawing the copy clock. This considers metrics such as absolute and relative duration of latencies, number of pauses, and relative time spent thinking versus actively drawing with pen on the paper. |
| 14 | COMSimpleMotor | Simple and Complex Motor on command clock | The graphomotor components involved in the process of drawing the command clock. This considers metrics such as pen stroke speeds and oscillatory motion and can be helpful in parsing out motor from non-motor cognitive functions. |
| 15 | COPSimpleMotor | Simple and Complex Motor on copy clock | The graphomotor components involved in the process of drawing the copy clock. This considers metrics such as pen stroke speeds and oscillatory motion and can be helpful in parsing out motor from non-motor cognitive functions. |
| 16 | COMSpatialReasoning | Spatial Reasoning on command clock | The spatial abilities demonstrated during the process of drawing the command clock. This considers metrics pertaining to the geometric properties of the drawing including the circularity of the clock circle, placement of clock components, and drawing placement on the page. |
| 17 | COPSpatialReasoning | Spatial Reasoning on copy clock | The spatial abilities demonstrated during the process of drawing the copy clock. This considers metrics pertaining to the geometric properties of the drawing including the circularity of the clock circle, placement of clock components, and drawing placement on the page. |
| 18 | COMStrokeCountConformity_s | Stroke Count Conformity on command clock | The deviation from the expected number of pen strokes in the drawing of the command clock. |
| 19 | COMTotalTime_s | Total Time on command clock | The total time spent completing the drawing of the command clock measured from the first touch of the pen on the paper to the last pen lift off the paper. |
| 20 | COMInkLength_s | Ink Length on command clock | The sum, in millimeters, of all pen stroke lengths used in the drawing of the command clock. |
| 21 | COMDrawingSize_s | Drawing Size on command clock | The size, in millimeters, of the clock face circle of the command clock |
| 22 | COMDrawingProcessEfficiency_s | Drawing Process Efficiency on command clock | A relative measure that combines Ink Length and Total Time on the command clock. |
| 23 | COMNoise_s | Noise on command clock | A measure of the drawing that includes non-standard pen strokes, cross- outs, and overwriting on the command clock. |
| 24 | COMPercentInkTime_s | Percent Ink Time on command clock | The percentage of the test time spent actively drawing with the pen on the paper for the command clock. |
| 25 | COMAverageSpeed_s | Average Speed on command clock | The average speed of the pen for all pen strokes used during the drawing of the clock face of the command clock. |
| 26 | COMMaxSpeed_s | Max Speed on command clock | The maximum speed of the pen on the page during the drawing of the clock face of the command clock. |
| 27 | COMInitiationSpeed_s | Initiation Speed on command clock | The speed of the pen when beginning to draw the clock face of the command clock. |
| 28 | COMTerminationSpeed_s | Termination Speed on command clock | The speed of the pen when finishing the clock face of the command clock. |
| 29 | COMOscillatoryMotion_s | Oscillatory Motion on command clock | A measure of how much the motion of the pen deviates from a smooth pen motion during the drawing process of the command clock. |
| 30 | COMPercentThinkTime_s | Percent Think Time on command clock | The percentage of the test time spent "thinking" (i.e., holding the pen off the page but not actively drawing), measured from the first touch of the pen on the paper to the last pen lift off the paper, on the command clock. |
| 31 | COMAverageLatency_s | Average Latency on command clock | The average duration of the latencies between each pen stroke of the command clock. |
| 32 | COMLatencyVariability_s | Latency Variability on command clock | The variability in the latencies throughout the drawing process of the command clock. |
| 33 | COMRelativeLongLatency_s | Relative Long Latency on command clock | A measure of the differences among the average latency and the longer latencies within the drawing of the command clock. |
| 34 | COMLongLatencyCount_s | Long Latency Count on command clock | The total number of latencies in drawing the command clock that are notably longer than the normative sample standard. |
| 35 | COMLongestLatency_s | Longest Latency on command clock | The duration of the longest latency in the drawing of the command clock. |
| 36 | COMClockfaceCircularity_s | Clock face Circularity on command clock | A measure of the roundness of the clock face circle on the command clock. |
| 37 | COMComponentPlacement_s | Component Placement on command clock | A measure of the spatial relationships among the drawing components on the command clock. |
| 38 | COMVerticalSpatialPlacement_s | Vertical Spatial Placement on command clock | A measure of the vertical position of the drawing on the page on the command clock. |
| 39 | COMHorizontalSpatialPlacement_s | Horizontal Spatial Placement on command clock | A measure of the horizontal position of the drawing on the page on the command clock. |
| 40 | COPStrokeCountConformity_s | Stroke Count Conformity on copy clock | The deviation from the expected number of pen strokes in the drawing of the copy clock. |
| 41 | COPTotalTime_s | Total Time on copy clock | The total time spent completing the drawing of the copy clock measured from the first touch of the pen on the paper to the last pen lift off the paper. |
| 42 | COPInkLength_s | Ink Length on copy clock | The sum, in millimeters, of all pen stroke lengths used in the drawing of the copy clock. |
| 43 | COPDrawingSize_s | Drawing Size on copy clock | The size, in millimeters, of the clock face circle of the copy clock. |
| 44 | COPDrawingProcessEfficiency_s | Drawing Process Efficiency on copy clock | A relative measure that combines Ink Length and Total Time on the copy clock. |
| 45 | COPNoise_s | Noise on copy clock | A measure of the drawing that includes non-standard pen strokes, cross- outs, and overwriting on the copy clock. |
| 46 | COPPercentInkTime_s | Percent Ink Time on copy clock | The percentage of the test time spent actively drawing with the pen on the paper for the copy clock. |
| 47 | COPAverageSpeed_s | Average Speed on copy clock | The average speed of the pen for all pen strokes used during the drawing of the clock face of the copy clock. |
| 48 | COPMaxSpeed_s | Max Speed on copy clock | The maximum speed of the pen on the page during the drawing of the clock face of the copy clock. |
| 49 | COPInitiationSpeed_s | Initiation Speed on copy clock | The speed of the pen when beginning to draw the clock face of the copy clock. |
| 50 | COPTerminationSpeed_s | Termination Speed on copy clock | The speed of the pen when finishing the clock face of the copy clock. |
| 51 | COPOscillatoryMotion_s | Oscillatory Motion on copy clock | A measure of how much the motion of the pen deviates from a smooth pen motion during the drawing process of the copy clock. |
| 52 | COPPercentThinkTime_s | Percent Think Time on copy clock | The percentage of the test time spent "thinking" (i.e., holding the pen off the page but not actively drawing), measured from the first touch of the pen on the paper to the last pen lift off the paper, on the copy clock. |
| 53 | COPAverageLatency_s | Average Latency on copy clock | The average duration of the latencies between each pen stroke of the copy clock. |
| 54 | COPLatencyVariability_s | Latency Variability on copy clock | The variability in the latencies throughout the drawing process of the copy clock. |
| 55 | COPRelativeLongLatency_s | Relative Long Latency on copy clock | A measure of the differences among the average latency and the longer latencies within the drawing of the copy clock. |
| 56 | COPLongLatencyCount_s | Long Latency Count on copy clock | The total number of latencies in drawing the copy clock that are notably longer than the normative sample standard. |
| 57 | COPLongestLatency_s | Longest Latency on copy clock | The duration of the longest latency in the drawing of the copy clock. |
| 58 | COPClockfaceCircularity_s | Clock face Circularity on copy clock | A measure of the roundness of the clock face circle on the copy clock. |
| 59 | COPComponentPlacement_s | Component Placement on copy clock | A measure of the spatial relationships among the drawing components on the copy clock. |
| 60 | COPVerticalSpatialPlacement_s | Vertical Spatial Placement on copy clock | A measure of the vertical position of the drawing on the page on the copy clock. |
| 61 | COPHorizontalSpatialPlacement_s | Horizontal Spatial Placement on copy clock | A measure of the horizontal position of the drawing on the page on the copy clock. |
| 62 | COMStrokeCountConformity | Stroke Count Conformity on command clock | The deviation from the expected number of pen strokes in the drawing of the command clock. |
| 63 | COMTotalTime | Total Time on command clock | The total time spent completing the drawing of the command clock measured from the first touch of the pen on the paper to the last pen lift off the paper. |
| 64 | COMInkLength | Ink Length on command clock | The sum, in millimeters, of all pen stroke lengths used in the drawing of the command clock. |
| 65 | COMDrawingSize | Drawing Size on command clock | The size, in millimeters, of the clock face circle of the command clock |
| 66 | COMDrawingProcessEfficiency | Drawing Process Efficiency on command clock | A relative measure that combines Ink Length and Total Time on the command clock. |
| 67 | COMNoise | Noise on command clock | A measure of the drawing that includes non-standard pen strokes, cross- outs, and overwriting on the command clock. |
| 68 | COMPercentInkTime | Percent Ink Time on command clock | The percentage of the test time spent actively drawing with the pen on the paper for the command clock. |
| 69 | COMAverageSpeed | Average Speed on command clock | The average speed of the pen for all pen strokes used during the drawing of the clock face of the command clock. |
| 70 | COMMaxSpeed | Max Speed on command clock | The maximum speed of the pen on the page during the drawing of the clock face of the command clock. |
| 71 | COMInitiationSpeed | Initiation Speed on command clock | The speed of the pen when beginning to draw the clock face of the command clock. |
| 72 | COMTerminationSpeed | Termination Speed on command clock | The speed of the pen when finishing the clock face of the command clock. |
| 73 | COMOscillatoryMotion | Oscillatory Motion on command clock | A measure of how much the motion of the pen deviates from a smooth pen motion during the drawing process of the command clock. |
| 74 | COMPercentThinkTime | Percent Think Time on command clock | The percentage of the test time spent "thinking" (i.e., holding the pen off the page but not actively drawing), measured from the first touch of the pen on the paper to the last pen lift off the paper, on the command clock. |
| 75 | COMAverageLatency | Average Latency on command clock | The average duration of the latencies between each pen stroke of the command clock. |
| 76 | COMLatencyVariability | Latency Variability on command clock | The variability in the latencies throughout the drawing process of the command clock. |
| 77 | COMRelativeLongLatency | Relative Long Latency on command clock | A measure of the differences among the average latency and the longer latencies within the drawing of the command clock. |
| 78 | COMLongLatencyCount | Long Latency Count on command clock | The total number of latencies in drawing the command clock that are notably longer than the normative sample standard. |
| 79 | COMLongestLatency | Longest Latency on command clock | The duration of the longest latency in the drawing of the command clock. |
| 80 | COMClockfaceCircularity | Clock face Circularity on command clock | A measure of the roundness of the clock face circle on the command clock. |
| 81 | COMComponentPlacement | Component Placement on command clock | A measure of the spatial relationships among the drawing components on the command clock. |
| 82 | COMVerticalSpatialPlacement | Vertical Spatial Placement on command clock | A measure of the vertical position of the drawing on the page on the command clock. |
| 83 | COMHorizontalSpatialPlacement | Horizontal Spatial Placement on command clock | A measure of the horizontal position of the drawing on the page on the command clock. |
| 84 | COPStrokeCountConformity | Stroke Count Conformity on copy clock | The deviation from the expected number of pen strokes in the drawing of the copy clock. |
| 85 | COPTotalTime | Total Time on copy clock | The total time spent completing the drawing of the copy clock measured from the first touch of the pen on the paper to the last pen lift off the paper. |
| 86 | COPInkLength | Ink Length on copy clock | The sum, in millimeters, of all pen stroke lengths used in the drawing of the copy clock. |
| 87 | COPDrawingSize | Drawing Size on copy clock | The size, in millimeters, of the clock face circle of the copy clock. |
| 88 | COPDrawingProcessEfficiency | Drawing Process Efficiency on copy clock | A relative measure that combines Ink Length and Total Time on the copy clock. |
| 89 | COPNoise | Noise on copy clock | A measure of the drawing that includes non-standard pen strokes, cross- outs, and overwriting on the copy clock. |
| 90 | COPPercentInkTime | Percent Ink Time on copy clock | The percentage of the test time spent actively drawing with the pen on the paper for the copy clock. |
| 91 | COPAverageSpeed | Average Speed on copy clock | The average speed of the pen for all pen strokes used during the drawing of the clock face of the copy clock. |
| 92 | COPMaxSpeed | Max Speed on copy clock | The maximum speed of the pen on the page during the drawing of the clock face of the copy clock. |
| 93 | COPInitiationSpeed | Initiation Speed on copy clock | The speed of the pen when beginning to draw the clock face of the copy clock. |
| 94 | COPTerminationSpeed | Termination Speed on copy clock | The speed of the pen when finishing the clock face of the copy clock. |
| 95 | COPOscillatoryMotion | Oscillatory Motion on copy clock | A measure of how much the motion of the pen deviates from a smooth pen motion during the drawing process of the copy clock. |
| 96 | COPPercentThinkTime | Percent Think Time on copy clock | The percentage of the test time spent "thinking" (i.e., holding the pen off the page but not actively drawing), measured from the first touch of the pen on the paper to the last pen lift off the paper, on the copy clock. |
| 97 | COPAverageLatency | Average Latency on copy clock | The average duration of the latencies between each pen stroke of the copy clock. |
| 98 | COPLatencyVariability | Latency Variability on copy clock | The variability in the latencies throughout the drawing process of the copy clock. |
| 99 | COPRelativeLongLatency | Relative Long Latency on copy clock | A measure of the differences among the average latency and the longer latencies within the drawing of the copy clock. |
| 100 | COPLongLatencyCount | Long Latency Count on copy clock | The total number of latencies in drawing the copy clock that are notably longer than the normative sample standard. |
| 101 | COPLongestLatency | Longest Latency on copy clock | The duration of the longest latency in the drawing of the copy clock. |
| 102 | COPClockfaceCircularity | Clock face Circularity on copy clock | A measure of the roundness of the clock face circle on the copy clock. |
| 103 | COPComponentPlacement | Component Placement on copy clock | A measure of the spatial relationships among the drawing components on the copy clock. |
| 104 | COPVerticalSpatialPlacement | Vertical Spatial Placement on copy clock | A measure of the vertical position of the drawing on the page on the copy clock. |
| 105 | COPHorizontalSpatialPlacement | Horizontal Spatial Placement on copy clock | A measure of the horizontal position of the drawing on the page on the copy clock. |
