# Supplementary material for: Associations Between the Digital Clock Drawing Test and Brain Volume: Large Community-Based Prospective Cohort (Framingham Heart Study)
Source: J Med Internet Res. 2022 Apr 15;24(4):e34513. doi: 10.2196/34513 (PMC9055470; doi:10.2196/34513)
Supplement: Multimedia Appendix 2 [file jmir_v24i4e34513_app2.docx]

**Multimedia Appendix 2.** Association between digital Clock Drawing Test composite scores and cortical gray matter volumes.

| dCDT composite score | Cortical gray matter | | | | Frontal cortical gray matter | | | | Parietal cortical gray matter | | | | Temporal cortical gray matter | | | | Occipital cortical gray matter | | |
| --- | --- | --- | --- | --- | --- | --- | --- | --- | --- | --- | --- | --- | --- | --- | --- | --- | --- | --- | --- |
|  | **Effect size** | **Standard error** | ***P* value ^a^** | **Effect size** | | **Standard error** | ***P* value ^a^** | **Effect size** | | **Standard error** | ***P* value ^a^** | **Effect size** | | **Standard error** | ***P* value ^a^** | **Effect size** | | **Standard error** | ***P* value ^a^** |
| dCDT_LMi | 4.7×10^-2^ | 1.9×10^-2^ | 1.4×10^-2^ | 2.0×10^-2^ | | 1.9×10^-2^ | 2.9×10^-1^ | 5.6×10^-2^ | | 2.3×10^-2^ | 1.4×10^-2^ | 6.5×10^-2^ | | 2.2×10^-2^ | 3.5×10^-3^ | 1.4×10^-2^ | | 2.4×10^-2^ | 5.5×10^-1^ |
| dCDT_LMd | 4.9×10^-2^ | 1.9×10^-2^ | 1.0×10^-2^ | 2.1×10^-2^ | | 1.9×10^-2^ | 2.7×10^-1^ | 6.5×10^-2^ | | 2.3×10^-2^ | 4.6×10^-3^ | 6.5×10^-2^ | | 2.2×10^-2^ | 3.7×10^-3^ | 1.2×10^-2^ | | 2.4×10^-2^ | 6.2×10^-1^ |
| dCDT_LMr | 3.9×10^-2^ | 1.9×10^-2^ | 3.6×10^-2^ | 1.8×10^-2^ | | 1.9×10^-2^ | 3.3×10^-1^ | 4.1×10^-2^ | | 2.3×10^-2^ | 7.0×10^-2^ | 5.4×10^-2^ | | 2.2×10^-2^ | 1.3×10^-2^ | 1.6×10^-2^ | | 2.3×10^-2^ | 5.1×10^-1^ |
| dCDT_VRi | 6.2×10^-2^ | 1.9×10^-2^ | **1.4×10^-3^** | 2.9×10^-2^ | | 2.0×10^-2^ | 1.4×10^-1^ | 8.7×10^-2^ | | 2.3×10^-2^ | **2.3×10^-4^** | 7.8×10^-2^ | | 2.3×10^-2^ | **6.6×10^-4^** | 1.2×10^-2^ | | 2.4×10^-2^ | 6.2×10^-1^ |
| dCDT_VRd | 6.1×10^-2^ | 1.9×10^-2^ | **1.7×10^-3^** | 2.7×10^-2^ | | 1.9×10^-2^ | 1.6×10^-1^ | 8.6×10^-2^ | | 2.3×10^-2^ | **2.3×10^-4^** | 7.7×10^-2^ | | 2.3×10^-2^ | **7.6×10^-4^** | 1.2×10^-2^ | | 2.4×10^-2^ | 6.3×10^-1^ |
| dCDT_VRr | 5.7×10^-2^ | 1.9×10^-2^ | 3.2×10^-3^ | 2.6×10^-2^ | | 1.9×10^-2^ | 1.9×10^-1^ | 8.5×10^-2^ | | 2.3×10^-2^ | **2.7×10^-4^** | 7.1×10^-2^ | | 2.3×10^-2^ | **1.7×10^-3^** | 6.9×10^-3^ | | 2.4×10^-2^ | 7.8×10^-1^ |
| dCDT_PASi | 4.9×10^-2^ | 1.9×10^-2^ | 1.0×10^-2^ | 2.1×10^-2^ | | 1.9×10^-2^ | 2.8×10^-1^ | 5.0×10^-2^ | | 2.3×10^-2^ | 2.9×10^-2^ | 7.4×10^-2^ | | 2.2×10^-2^ | **9.7×10^-4^** | 1.7×10^-2^ | | 2.4×10^-2^ | 4.8×10^-1^ |
| dCDT_PASd | 4.8×10^-2^ | 1.9×10^-2^ | 1.2×10^-2^ | 1.6×10^-2^ | | 1.9×10^-2^ | 4.0×10^-1^ | 6.9×10^-2^ | | 2.3×10^-2^ | 2.9×10^-3^ | 6.5×10^-2^ | | 2.3×10^-2^ | 4.3×10^-3^ | 1.5×10^-2^ | | 2.4×10^-2^ | 5.4×10^-1^ |
| dCDT_PASr | 3.8×10^-2^ | 1.9×10^-2^ | 4.5×10^-2^ | 9.1×10^-3^ | | 1.9×10^-2^ | 6.3×10^-1^ | 5.9×10^-2^ | | 2.3×10^-2^ | 9.9×10^-3^ | 5.6×10^-2^ | | 2.2×10^-2^ | 1.2×10^-2^ | 7.6×10^-3^ | | 2.4×10^-2^ | 7.5×10^-1^ |
| dCDT_DSf | 4.5×10^-2^ | 1.9×10^-2^ | 1.6×10^-2^ | 2.0×10^-2^ | | 1.9×10^-2^ | 3.0×10^-1^ | 6.1×10^-2^ | | 2.3×10^-2^ | 7.2×10^-3^ | 5.8×10^-2^ | | 2.2×10^-2^ | 9.3×10^-3^ | 1.4×10^-2^ | | 2.4×10^-2^ | 5.7×10^-1^ |
| dCDT_DSb | 4.2×10^-2^ | 1.9×10^-2^ | 2.6×10^-2^ | 1.9×10^-2^ | | 1.9×10^-2^ | 3.1×10^-1^ | 4.4×10^-2^ | | 2.3×10^-2^ | 5.2×10^-2^ | 6.0×10^-2^ | | 2.2×10^-2^ | 6.6×10^-3^ | 1.5×10^-2^ | | 2.4×10^-2^ | 5.3×10^-1^ |
| dCDT_Trails A | -4.6×10^-2^ | 1.9×10^-2^ | 1.4×10^-2^ | -2.2×10^-2^ | | 1.9×10^-2^ | 2.6×10^-1^ | -9.2×10^-2^ | | 2.3×10^-2^ | **6.0×10^-5^** | -4.7×10^-2^ | | 2.2×10^-2^ | 3.4×10^-2^ | 7.0×10^-3^ | | 2.4×10^-2^ | 7.7×10^-1^ |
| dCDT_Trails B | -4.9×10^-2^ | 1.9×10^-2^ | 9.8×10^-3^ | -2.2×10^-2^ | | 1.9×10^-2^ | 2.5×10^-1^ | -8.9×10^-2^ | | 2.3×10^-2^ | **1.1×10^-4^** | -5.5×10^-2^ | | 2.2×10^-2^ | 1.5×10^-2^ | 1.7×10^-3^ | | 2.4×10^-2^ | 9.4×10^-1^ |
| dCDT_SIM | 5.2×10^-2^ | 1.9×10^-2^ | 6.2×10^-3^ | 2.6×10^-2^ | | 1.9×10^-2^ | 1.7×10^-1^ | 6.2×10^-2^ | | 2.3×10^-2^ | 7.7×10^-3^ | 7.2×10^-2^ | | 2.2×10^-2^ | **1.5×10^-3^** | 1.1×10^-2^ | | 2.4×10^-2^ | 6.4×10^-1^ |
| dCDT_HVOT | 5.5×10^-2^ | 1.9×10^-2^ | 4.2×10^-3^ | 2.2×10^-2^ | | 1.9×10^-2^ | 2.5×10^-1^ | 7.7×10^-2^ | | 2.3×10^-2^ | **9.9×10^-4^** | 7.3×10^-2^ | | 2.3×10^-2^ | **1.4×10^-3^** | 1.4×10^-2^ | | 2.4×10^-2^ | 5.6×10^-1^ |
| dCDT_BNT30 | 4.1×10^-2^ | 1.9×10^-2^ | 3.0×10^-2^ | 1.4×10^-2^ | | 1.9×10^-2^ | 4.7×10^-1^ | 5.4×10^-2^ | | 2.3×10^-2^ | 1.8×10^-2^ | 5.9×10^-2^ | | 2.2×10^-2^ | 8.7×10^-3^ | 1.4×10^-2^ | | 2.4×10^-2^ | 5.6×10^-1^ |
| dCDT_FAS | 5.0×10^-2^ | 1.9×10^-2^ | 9.1×10^-3^ | 2.2×10^-2^ | | 1.9×10^-2^ | 2.5×10^-1^ | 8.0×10^-2^ | | 2.3×10^-2^ | **5.2×10^-4^** | 5.9×10^-2^ | | 2.3×10^-2^ | 8.8×10^-3^ | 4.7×10^-3^ | | 2.4×10^-2^ | 8.5×10^-1^ |
| dCDT_FAS-animal | 3.9×10^-2^ | 1.9×10^-2^ | 3.7×10^-2^ | 1.7×10^-2^ | | 1.9×10^-2^ | 3.6×10^-1^ | 4.3×10^-2^ | | 2.3×10^-2^ | 5.5×10^-2^ | 5.4×10^-2^ | | 2.2×10^-2^ | 1.5×10^-2^ | 1.6×10^-2^ | | 2.4×10^-2^ | 5.0×10^-1^ |

The models were adjusted for age, sex, and education. All MRI measures were the percent of these volumes over the total cerebral cranial volume (TCV) above the tentorium.

^a^ Bonferroni correction was used to adjust for multiple testing, and significant associations were claimed if *p*<0.05/18 (2.8×10^-3^) and indicated in bold, where 18 was the number of tests performed.
