# Supplementary material for: Associations Between the Digital Clock Drawing Test and Brain Volume: Large Community-Based Prospective Cohort (Framingham Heart Study)
Source: J Med Internet Res. 2022 Apr 15;24(4):e34513. doi: 10.2196/34513 (PMC9055470; doi:10.2196/34513)
Supplement: Multimedia Appendix 3 [file jmir_v24i4e34513_app3.docx]

**Multimedia Appendix 3.** Association between digital Clock Drawing Test composite scores and total cerebral brain volume after additionally adjusting for vascular risk factors.

| dCDT composite score | Effect size | Standard error | *P* value ^a^ |
| --- | --- | --- | --- |
| dCDT_LMi | 9.2×10^-2^ | 1.8×10^-2^ | **5.6×10^-7^** |
| dCDT_LMd | 9.6×10^-2^ | 1.8×10^-2^ | **2.1×10^-7^** |
| dCDT_LMr | 7.3×10^-2^ | 1.8×10^-2^ | **6.6×10^-5^** |
| dCDT_VRi | 1.1×10^-1^ | 1.9×10^-2^ | **5.9×10^-9^** |
| dCDT_VRd | 1.1×10^-1^ | 1.9×10^-2^ | **6.7×10^-9^** |
| dCDT_VRr | 1.1×10^-1^ | 1.9×10^-2^ | **1.9×10^-8^** |
| dCDT_PASi | 8.9×10^-2^ | 1.9×10^-2^ | **1.6×10^-6^** |
| dCDT_PASd | 9.9×10^-2^ | 1.9×10^-2^ | **1.3×10^-7^** |
| dCDT_PASr | 8.4×10^-2^ | 1.8×10^-2^ | **4.3×10^-6^** |
| dCDT_DSf | 8.4×10^-2^ | 1.8×10^-2^ | **5.0×10^-6^** |
| dCDT_DSb | 8.0×10^-2^ | 1.8×10^-2^ | **1.3×10^-5^** |
| dCDT_Trails A | -8.7×10^-2^ | 1.8×10^-2^ | **2.3×10^-6^** |
| dCDT_Trails B | -9.4×10^-2^ | 1.9×10^-2^ | **4.9×10^-7^** |
| dCDT_SIM | 9.6×10^-2^ | 1.9×10^-2^ | **2.7×10^-7^** |
| dCDT_HVOT | 1.1×10^-1^ | 1.9×10^-2^ | **1.5×10^-8^** |
| dCDT_BNT30 | 8.9×10^-2^ | 1.9×10^-2^ | **1.8×10^-6^** |
| dCDT_FAS | 9.9×10^-2^ | 1.9×10^-2^ | **1.3×10^-7^** |
| dCDT_FAS-animal | 7.5×10^-2^ | 1.8×10^-2^ | **4.3×10^-5^** |

The model was adjusted for age, sex, education, and vascular risk factors (hypertension, diabetes, smoking and atrial fibrillation). TCBV is the percent of total Cerebral brain volume over the total cranial volume (TCV) above the tentorium.

^a^ Bonferroni correction was used to adjust for multiple testing, and significant associations were claimed if *p*<0.05/18 (2.8×10^-3^) and indicated in bold, where 18 was the number of tests performed.
