# Supplementary material for: Associations Between the Digital Clock Drawing Test and Brain Volume: Large Community-Based Prospective Cohort (Framingham Heart Study)
Source: J Med Internet Res. 2022 Apr 15;24(4):e34513. doi: 10.2196/34513 (PMC9055470; doi:10.2196/34513)
Supplement: Multimedia Appendix 4 [file jmir_v24i4e34513_app4.docx]

**Multimedia Appendix 4.** Association of digital Clock Drawing Test composite scores with cerebral white matter, gray matter, and hippocampal volumes after additionally adjusting for vascular risk factors.

| dCDT  composite score | Cerebral white matter volume | | | Cerebral gray matter volume | | | Hippocampal volume | | |
| --- | --- | --- | --- | --- | --- | --- | --- | --- | --- |
|  | **Effect size** | **Standard error** | ***P* value ^a^** | **Effect size** | **Standard error** | ***P* value ^a^** | **Effect size** | **Standard error** | ***P* value ^a^** |
| dCDT_LMi | 8.4×10^-2^ | 2.5×10^-2^ | **7.7×10^-4^** | 5.0×10^-2^ | 2.1×10^-2^ | 1.7×10^-2^ | 4.0×10^-2^ | 2.8×10^-2^ | 1.6×10^-1^ |
| dCDT_LMd | 8.6×10^-2^ | 2.5×10^-2^ | **5.7×10^-4^** | 5.4×10^-2^ | 2.1×10^-2^ | 1.0×10^-2^ | 4.3×10^-2^ | 2.8×10^-2^ | 1.3×10^-1^ |
| dCDT_LMr | 6.8×10^-2^ | 2.4×10^-2^ | 5.5×10^-3^ | 3.7×10^-2^ | 2.1×10^-2^ | 7.3×10^-2^ | 2.7×10^-2^ | 2.8×10^-2^ | 3.2×10^-1^ |
| dCDT_VRi | 9.2×10^-2^ | 2.5×10^-2^ | **3.3×10^-4^** | 6.9×10^-2^ | 2.1×10^-2^ | **1.5×10^-3^** | 5.7×10^-2^ | 2.9×10^-2^ | 4.6×10^-2^ |
| dCDT_VRd | 9.2×10^-2^ | 2.5×10^-2^ | **2.9×10^-4^** | 6.7×10^-2^ | 2.1×10^-2^ | **1.7×10^-3^** | 5.6×10^-2^ | 2.9×10^-2^ | 4.9×10^-2^ |
| dCDT_VRr | 8.9×10^-2^ | 2.5×10^-2^ | **4.4×10^-4^** | 6.5×10^-2^ | 2.1×10^-2^ | **2.4×10^-3^** | 5.3×10^-2^ | 2.9×10^-2^ | 6.2×10^-2^ |
| dCDT_PASi | 8.3×10^-2^ | 2.5×10^-2^ | **1.0×10^-3^** | 4.9×10^-2^ | 2.1×10^-2^ | 2.1×10^-2^ | 4.5×10^-2^ | 2.8×10^-2^ | 1.1×10^-1^ |
| dCDT_PASd | 9.0×10^-2^ | 2.5×10^-2^ | **3.5×10^-4^** | 5.5×10^-2^ | 2.1×10^-2^ | 1.0×10^-2^ | 4.6×10^-2^ | 2.8×10^-2^ | 1.1×10^-1^ |
| dCDT_PASr | 7.0×10^-2^ | 2.5×10^-2^ | 4.6×10^-3^ | 5.1×10^-2^ | 2.1×10^-2^ | 1.3×10^-2^ | 4.7×10^-2^ | 2.8×10^-2^ | 9.2×10^-2^ |
| dCDT_DSf | 7.7×10^-2^ | 2.5×10^-2^ | **1.8×10^-3^** | 4.6×10^-2^ | 2.1×10^-2^ | 2.7×10^-2^ | 3.1×10^-2^ | 2.8×10^-2^ | 2.7×10^-1^ |
| dCDT_DSb | 7.5×10^-2^ | 2.5×10^-2^ | **2.6×10^-3^** | 4.2×10^-2^ | 2.1×10^-2^ | 4.6×10^-2^ | 3.3×10^-2^ | 2.8×10^-2^ | 2.3×10^-1^ |
| dCDT_Trails A | -7.0×10^-2^ | 2.5×10^-2^ | 4.8×10^-3^ | -5.8×10^-2^ | 2.1×10^-2^ | 6.0×10^-3^ | -4.2×10^-2^ | 2.8×10^-2^ | 1.4×10^-1^ |
| dCDT_Trails B | -7.8×10^-2^ | 2.5×10^-2^ | **2.0×10^-3^** | -6.0×10^-2^ | 2.1×10^-2^ | 4.9×10^-3^ | -4.4×10^-2^ | 2.8×10^-2^ | 1.2×10^-1^ |
| dCDT_SIM | 8.4×10^-2^ | 2.5×10^-2^ | **8.7×10^-4^** | 5.5×10^-2^ | 2.1×10^-2^ | 9.8×10^-3^ | 4.1×10^-2^ | 2.8×10^-2^ | 1.5×10^-1^ |
| dCDT_HVOT | 9.3×10^-2^ | 2.5×10^-2^ | **2.6×10^-4^** | 6.3×10^-2^ | 2.2×10^-2^ | 3.7×10^-3^ | 5.2×10^-2^ | 2.9×10^-2^ | 6.9×10^-2^ |
| dCDT_BNT30 | 8.5×10^-2^ | 2.5×10^-2^ | **7.5×10^-4^** | 4.5×10^-2^ | 2.1×10^-2^ | 3.6×10^-2^ | 3.5×10^-2^ | 2.8×10^-2^ | 2.1×10^-1^ |
| dCDT_FAS | 8.4×10^-2^ | 2.5×10^-2^ | **8.7×10^-4^** | 5.9×10^-2^ | 2.1×10^-2^ | 6.2×10^-3^ | 4.2×10^-2^ | 2.8×10^-2^ | 1.4×10^-1^ |
| dCDT_FAS-animal | 7.0×10^-2^ | 2.5×10^-2^ | 4.6×10^-3^ | 3.8×10^-2^ | 2.1×10^-2^ | 6.5×10^-2^ | 3.0×10^-2^ | 2.8×10^-2^ | 2.8×10^-1^ |

The model was adjusted for age, sex, education, and vascular risk factors (hypertension, diabetes, smoking and atrial fibrillation). All MRI measures were the percent of these volumes over the total cerebral cranial volume (TCV) above the tentorium.

^a^ Bonferroni correction was used to adjust for multiple testing, and significant associations were claimed if *p*<0.05/18 (2.8×10^-3^) and indicated in bold, where 18 was the number of tests performed.
