# Supplementary material for: Associations Between the Digital Clock Drawing Test and Brain Volume: Large Community-Based Prospective Cohort (Framingham Heart Study)
Source: J Med Internet Res. 2022 Apr 15;24(4):e34513. doi: 10.2196/34513 (PMC9055470; doi:10.2196/34513)
Supplement: Multimedia Appendix 5 [file jmir_v24i4e34513_app5.docx]

**Multimedia Appendix 5.** Association between digital Clock Drawing Test composite scores and white matter hyperintensity volume after additionally adjusting for vascular risk factors.

| dCDT  composite score | WMH volume | | | WMH-Large | | |
| --- | --- | --- | --- | --- | --- | --- |
|  | **Effect size** | **Standard error** | ***P* value ^a^** | **Effect size** | **Standard error** | ***P* value ^a^** |
| dCDT_LMi | -4.5×10^-2^ | 2.0×10^-2^ | 2.2×10^-2^ | -1.7×10^-1^ | 7.9×10^-2^ | 3.0×10^-2^ |
| dCDT_LMd | -4.7×10^-2^ | 2.0×10^-2^ | 1.9×10^-2^ | -1.8×10^-1^ | 7.9×10^-2^ | 2.3×10^-2^ |
| dCDT_LMr | -4.5×10^-2^ | 1.9×10^-2^ | 2.2×10^-2^ | -1.7×10^-1^ | 7.7×10^-2^ | 3.0×10^-2^ |
| dCDT_VRi | -4.5×10^-2^ | 2.0×10^-2^ | 2.5×10^-2^ | -2.0×10^-1^ | 8.1×10^-2^ | 1.2×10^-2^ |
| dCDT_VRd | -4.5×10^-2^ | 2.0×10^-2^ | 2.7×10^-2^ | -2.0×10^-1^ | 8.1×10^-2^ | 1.3×10^-2^ |
| dCDT_VRr | -4.5×10^-2^ | 2.0×10^-2^ | 2.4×10^-2^ | -2.1×10^-1^ | 8.1×10^-2^ | 9.4×10^-3^ |
| dCDT_PASi | -4.1×10^-2^ | 2.0×10^-2^ | 3.9×10^-2^ | -1.5×10^-1^ | 7.9×10^-2^ | 5.3×10^-2^ |
| dCDT_PASd | -4.8×10^-2^ | 2.0×10^-2^ | 1.8×10^-2^ | -1.8×10^-1^ | 8.0×10^-2^ | 2.1×10^-2^ |
| dCDT_PASr | -2.5×10^-2^ | 2.0×10^-2^ | 2.0×10^-1^ | -7.6×10^-2^ | 7.9×10^-2^ | 3.4×10^-1^ |
| dCDT_DSf | -4.9×10^-2^ | 2.0×10^-2^ | 1.4×10^-2^ | -2.1×10^-1^ | 7.8×10^-2^ | 7.1×10^-3^ |
| dCDT_DSb | -4.2×10^-2^ | 2.0×10^-2^ | 3.2×10^-2^ | -1.5×10^-1^ | 7.8×10^-2^ | 4.8×10^-2^ |
| dCDT_Trails A | 4.0×10^-2^ | 2.0×10^-2^ | 4.2×10^-2^ | 2.1×10^-1^ | 8.0×10^-2^ | 9.9×10^-3^ |
| dCDT_Trails B | 4.2×10^-2^ | 2.0×10^-2^ | 3.4×10^-2^ | 2.1×10^-1^ | 8.1×10^-2^ | 1.0×10^-2^ |
| dCDT_SIM | -4.1×10^-2^ | 2.0×10^-2^ | 3.9×10^-2^ | -1.8×10^-1^ | 7.9×10^-2^ | 2.6×10^-2^ |
| dCDT_HVOT | -4.6×10^-2^ | 2.0×10^-2^ | 2.4×10^-2^ | -2.0×10^-1^ | 8.1×10^-2^ | 1.5×10^-2^ |
| dCDT_BNT30 | -4.7×10^-2^ | 2.0×10^-2^ | 1.7×10^-2^ | -1.8×10^-1^ | 7.9×10^-2^ | 2.0×10^-2^ |
| dCDT_FAS | -4.6×10^-2^ | 2.0×10^-2^ | 2.2×10^-2^ | -2.1×10^-1^ | 8.1×10^-2^ | 9.7×10^-3^ |
| dCDT_FAS-animal | -4.5×10^-2^ | 1.9×10^-2^ | 2.0×10^-2^ | -1.7×10^-1^ | 7.7×10^-2^ | 3.0×10^-2^ |

The model was adjusted for age, sex, education, and vascular risk factors (hypertension, diabetes, smoking and atrial fibrillation). The white matter hyperintensity (WMH) volume was the percent over the total cerebral cranial volume (TCV) above the tentorium and was log-transformed. The large WMH volume (WMH-Large) was defined as those with more than one standard deviation higher than the age-specific mean values.

^a^ Bonferroni correction was used to adjust for multiple testing, and significant associations were claimed if *p*<0.05/18 (2.8×10^-3^) and indicated in bold, where 18 was the number of tests performed.
