# Supplementary material for: Associations Between the Digital Clock Drawing Test and Brain Volume: Large Community-Based Prospective Cohort (Framingham Heart Study)
Source: J Med Internet Res. 2022 Apr 15;24(4):e34513. doi: 10.2196/34513 (PMC9055470; doi:10.2196/34513)
Supplement: Multimedia Appendix 6 [file jmir_v24i4e34513_app6.docx]

**Multimedia Appendix 6.** Association between digital Clock Drawing Test composite scores and cortical gray matter after additionally adjusting for vascular risk factors.

| dCDT composite score | Cortical gray matter | | | | Frontal cortical gray matter | | | | Parietal cortical gray matter | | | | Temporal cortical gray matter | | | | Occipital cortical gray matter | | | |
| --- | --- | --- | --- | --- | --- | --- | --- | --- | --- | --- | --- | --- | --- | --- | --- | --- | --- | --- | --- | --- |
|  | **Effect size** | **Standard error** | ***P* value ^a^** | **Effect size** | | **Standard error** | ***P* value ^a^** | **Effect size** | | **Standard error** | ***P* value ^a^** | **Effect size** | | **Standard error** | ***P* value ^a^** | **Effect size** | | **Standard error** | ***P* value ^a^** |  |
| dCDT_LMi | 5.5×10^-2^ | 2.1×10^-2^ | 7.5×10^-3^ | 3.2×10^-2^ | | 2.1×10^-2^ | 1.3×10^-1^ | 6.4×10^-2^ | | 2.5×10^-2^ | 1.1×10^-2^ | 7.4×10^-2^ | | 2.5×10^-2^ | **2.5×10^-3^** | 6.4×10^-3^ | | 2.6×10^-2^ | 8.1×10^-1^ |  |
| dCDT_LMd | 5.9×10^-2^ | 2.1×10^-2^ | 4.6×10^-3^ | 3.6×10^-2^ | | 2.1×10^-2^ | 9.0×10^-2^ | 7.4×10^-2^ | | 2.5×10^-2^ | 3.5×10^-3^ | 7.3×10^-2^ | | 2.5×10^-2^ | 3.1×10^-3^ | 4.8×10^-3^ | | 2.6×10^-2^ | 8.5×10^-1^ |  |
| dCDT_LMr | 4.2×10^-2^ | 2.0×10^-2^ | 3.7×10^-2^ | 2.5×10^-2^ | | 2.1×10^-2^ | 2.3×10^-1^ | 4.7×10^-2^ | | 2.5×10^-2^ | 6.0×10^-2^ | 6.0×10^-2^ | | 2.4×10^-2^ | 1.4×10^-2^ | 3.7×10^-3^ | | 2.6×10^-2^ | 8.8×10^-1^ |  |
| dCDT_VRi | 7.2×10^-2^ | 2.1×10^-2^ | **7.1×10^-4^** | 4.4×10^-2^ | | 2.1×10^-2^ | 4.1×10^-2^ | 9.7×10^-2^ | | 2.6×10^-2^ | **1.7×10^-4^** | 8.4×10^-2^ | | 2.5×10^-2^ | **8.4×10^-4^** | 3.5×10^-3^ | | 2.7×10^-2^ | 8.9×10^-1^ |  |
| dCDT_VRd | 7.1×10^-2^ | 2.1×10^-2^ | **8.4×10^-4^** | 4.2×10^-2^ | | 2.1×10^-2^ | 4.7×10^-2^ | 9.6×10^-2^ | | 2.6×10^-2^ | **2.0×10^-4^** | 8.4×10^-2^ | | 2.5×10^-2^ | **8.7×10^-4^** | 4.0×10^-3^ | | 2.7×10^-2^ | 8.8×10^-1^ |  |
| dCDT_VRr | 6.8×10^-2^ | 2.1×10^-2^ | **1.3×10^-3^** | 4.1×10^-2^ | | 2.1×10^-2^ | 5.2×10^-2^ | 9.7×10^-2^ | | 2.6×10^-2^ | **1.6×10^-4^** | 7.8×10^-2^ | | 2.5×10^-2^ | **1.9×10^-3^** | 1.0×10^-3^ | | 2.7×10^-2^ | 9.7×10^-1^ |  |
| dCDT_PASi | 5.5×10^-2^ | 2.1×10^-2^ | 9.1×10^-3^ | 2.7×10^-2^ | | 2.1×10^-2^ | 2.0×10^-1^ | 6.2×10^-2^ | | 2.5×10^-2^ | 1.5×10^-2^ | 8.3×10^-2^ | | 2.5×10^-2^ | **8.2×10^-4^** | 4.6×10^-3^ | | 2.6×10^-2^ | 8.6×10^-1^ |  |
| dCDT_PASd | 6.0×10^-2^ | 2.1×10^-2^ | 4.5×10^-3^ | 3.2×10^-2^ | | 2.1×10^-2^ | 1.4×10^-1^ | 8.2×10^-2^ | | 2.6×10^-2^ | **1.3×10^-3^** | 7.2×10^-2^ | | 2.5×10^-2^ | 3.9×10^-3^ | 8.8×10^-3^ | | 2.6×10^-2^ | 7.4×10^-1^ |  |
| dCDT_PASr | 5.4×10^-2^ | 2.1×10^-2^ | 9.0×10^-3^ | 3.1×10^-2^ | | 2.1×10^-2^ | 1.3×10^-1^ | 7.0×10^-2^ | | 2.5×10^-2^ | 4.9×10^-3^ | 6.3×10^-2^ | | 2.4×10^-2^ | 1.0×10^-2^ | 8.6×10^-3^ | | 2.6×10^-2^ | 7.4×10^-1^ |  |
| dCDT_DSf | 5.2×10^-2^ | 2.1×10^-2^ | 1.2×10^-2^ | 2.8×10^-2^ | | 2.1×10^-2^ | 1.8×10^-1^ | 7.3×10^-2^ | | 2.5×10^-2^ | 3.6×10^-3^ | 6.3×10^-2^ | | 2.5×10^-2^ | 1.0×10^-2^ | 3.6×10^-3^ | | 2.6×10^-2^ | 8.9×10^-1^ |  |
| dCDT_DSb | 4.7×10^-2^ | 2.1×10^-2^ | 2.2×10^-2^ | 2.4×10^-2^ | | 2.1×10^-2^ | 2.4×10^-1^ | 5.4×10^-2^ | | 2.5×10^-2^ | 3.3×10^-2^ | 6.9×10^-2^ | | 2.4×10^-2^ | 5.0×10^-3^ | 5.0×10^-3^ | | 2.6×10^-2^ | 8.5×10^-1^ |  |
| dCDT_Trails A | -5.9×10^-2^ | 2.1×10^-2^ | 4.4×10^-3^ | -4.2×10^-2^ | | 2.1×10^-2^ | 4.5×10^-2^ | -1.0×10^-1^ | | 2.5×10^-2^ | **4.2×10^-5^** | -4.9×10^-2^ | | 2.5×10^-2^ | 4.5×10^-2^ | 9.3×10^-3^ | | 2.6×10^-2^ | 7.2×10^-1^ |  |
| dCDT_Trails B | -6.2×10^-2^ | 2.1×10^-2^ | 3.0×10^-3^ | -4.1×10^-2^ | | 2.1×10^-2^ | 5.2×10^-2^ | -1.0×10^-1^ | | 2.5×10^-2^ | **7.7×10^-5^** | -6.0×10^-2^ | | 2.5×10^-2^ | 1.6×10^-2^ | 5.0×10^-3^ | | 2.6×10^-2^ | 8.5×10^-1^ |  |
| dCDT_SIM | 5.9×10^-2^ | 2.1×10^-2^ | 4.5×10^-3^ | 3.3×10^-2^ | | 2.1×10^-2^ | 1.2×10^-1^ | 7.3×10^-2^ | | 2.5×10^-2^ | 4.3×10^-3^ | 8.1×10^-2^ | | 2.5×10^-2^ | **1.1×10^-3^** | 3.8×10^-3^ | | 2.6×10^-2^ | 8.9×10^-1^ |  |
| dCDT_HVOT | 6.6×10^-2^ | 2.1×10^-2^ | **1.8×10^-3^** | 3.8×10^-2^ | | 2.1×10^-2^ | 7.6×10^-2^ | 9.0×10^-2^ | | 2.6×10^-2^ | **4.9×10^-4^** | 8.0×10^-2^ | | 2.5×10^-2^ | **1.6×10^-3^** | 5.6×10^-3^ | | 2.7×10^-2^ | 8.3×10^-1^ |  |
| dCDT_BNT30 | 5.0×10^-2^ | 2.1×10^-2^ | 1.7×10^-2^ | 2.5×10^-2^ | | 2.1×10^-2^ | 2.3×10^-1^ | 6.7×10^-2^ | | 2.5×10^-2^ | 8.4×10^-3^ | 6.6×10^-2^ | | 2.5×10^-2^ | 7.5×10^-3^ | 3.1×10^-3^ | | 2.6×10^-2^ | 9.1×10^-1^ |  |
| dCDT_FAS | 6.2×10^-2^ | 2.1×10^-2^ | 3.2×10^-3^ | 3.7×10^-2^ | | 2.1×10^-2^ | 7.9×10^-2^ | 9.4×10^-2^ | | 2.6×10^-2^ | **2.3×10^-4^** | 6.6×10^-2^ | | 2.5×10^-2^ | 8.4×10^-3^ | 1.8×10^-3^ | | 2.6×10^-2^ | 9.5×10^-1^ |  |
| dCDT_FAS-animal | 4.3×10^-2^ | 2.0×10^-2^ | 3.4×10^-2^ | 2.5×10^-2^ | | 2.1×10^-2^ | 2.3×10^-1^ | 4.9×10^-2^ | | 2.5×10^-2^ | 4.7×10^-2^ | 5.9×10^-2^ | | 2.4×10^-2^ | 1.5×10^-2^ | 5.2×10^-3^ | | 2.6×10^-2^ | 8.4×10^-1^ |  |

The model was adjusted for age, sex, education, and vascular risk factors (hypertension, diabetes, smoking and atrial fibrillation). All MRI measures were the percent of these volumes over the total cerebral cranial volume (TCV) above the tentorium.

^a^ Bonferroni correction was used to adjust for multiple testing, and significant associations were claimed if *p*<0.05/18 (2.8×10^-3^) and indicated in bold, where 18 was the number of tests performed.
