# Supplementary material for: Associations Between the Digital Clock Drawing Test and Brain Volume: Large Community-Based Prospective Cohort (Framingham Heart Study)
Source: J Med Internet Res. 2022 Apr 15;24(4):e34513. doi: 10.2196/34513 (PMC9055470; doi:10.2196/34513)
Supplement: Multimedia Appendix 7 [file jmir_v24i4e34513_app7.docx]

**Multimedia Appendix 7.** Association between individual digital Clock Drawing Test features and magnetic resonance imaging measures.

| MRI measures ^a^ | dCDT feature | β | SE | *P* value ^b^ |
| --- | --- | --- | --- | --- |
| Total Cerebral brain volume | DCTScore | 1.0×10^-1^ | 1.7×10^-2^ | **8.3×10^-9^** |
| Total Cerebral brain volume | COPOscillatoryMotion | -9.6×10^-2^ | 1.7×10^-2^ | **9.3×10^-9^** |
| Total Cerebral brain volume | COPOscillatoryMotion_s | 8.2×10^-2^ | 1.6×10^-2^ | **4.2×10^-7^** |
| Total Cerebral brain volume | COMOscillatoryMotion | -8.1×10^-2^ | 1.7×10^-2^ | **3.0×10^-6^** |
| Total Cerebral brain volume | COPSimpleMotor | 7.3×10^-2^ | 1.6×10^-2^ | **9.7×10^-6^** |
| Total Cerebral brain volume | COPTotalTime | -7.2×10^-2^ | 1.7×10^-2^ | **2.5×10^-5^** |
| Total Cerebral brain volume | COMDrawingProcessEfficiency | 7.1×10^-2^ | 1.7×10^-2^ | **3.1×10^-5^** |
| Total Cerebral brain volume | COPDrawingProcessEfficiency | 6.9×10^-2^ | 1.7×10^-2^ | **4.8×10^-5^** |
| Total Cerebral brain volume | COMOscillatoryMotion_s | 6.7×10^-2^ | 1.7×10^-2^ | **6.4×10^-5^** |
| Total Cerebral brain volume | COPDrawingEfficiency | 6.5×10^-2^ | 1.7×10^-2^ | **1.0×10^-4^** |
| Total Cerebral brain volume | COPAverageLatency | -6.3×10^-2^ | 1.7×10^-2^ | **1.8×10^-4^** |
| Total Cerebral brain volume | COMAverageLatency | -6.2×10^-2^ | 1.7×10^-2^ | **2.4×10^-4^** |
| Total Cerebral brain volume | COMSimpleMotor | 6.2×10^-2^ | 1.7×10^-2^ | **2.5×10^-4^** |
| Total Cerebral brain volume | COPSpatialReasoning | 6.1×10^-2^ | 1.7×10^-2^ | **2.7×10^-4^** |
| Total Cerebral brain volume | COMDrawingProcessEfficiency_s | 6.0×10^-2^ | 1.7×10^-2^ | **2.8×10^-4^** |
| Total Cerebral brain volume | COPSimpleMotor_s | 5.8×10^-2^ | 1.6×10^-2^ | **3.1×10^-4^** |
| Total Cerebral brain volume | COPDrawingProcessEfficiency_s | 5.9×10^-2^ | 1.7×10^-2^ | **4.3×10^-4^** |
| Total Cerebral brain volume | COPTotalTime_s | 5.6×10^-2^ | 1.6×10^-2^ | 6.5×10^-4^ |
| Total Cerebral brain volume | COMComponentPlacement | -5.6×10^-2^ | 1.7×10^-2^ | 9.3×10^-4^ |
| Total Cerebral brain volume | COPDrawingEfficiency_s | 5.5×10^-2^ | 1.7×10^-2^ | 1.0×10^-3^ |
| Total Cerebral brain volume | COPComponentPlacement | -5.4×10^-2^ | 1.7×10^-2^ | 1.1×10^-3^ |
| Total Cerebral brain volume | COPInformationProcessing | 5.5×10^-2^ | 1.7×10^-2^ | 1.2×10^-3^ |
| Total Cerebral brain volume | COPAverageLatency_s | 5.4×10^-2^ | 1.7×10^-2^ | 1.2×10^-3^ |
| Total Cerebral brain volume | COPSpatialReasoning_s | 5.2×10^-2^ | 1.6×10^-2^ | 1.4×10^-3^ |
| Total Cerebral brain volume | COMTotalTime | -5.3×10^-2^ | 1.7×10^-2^ | 1.5×10^-3^ |
| Total Cerebral brain volume | COMAverageLatency_s | 5.2×10^-2^ | 1.7×10^-2^ | 1.7×10^-3^ |
| Total Cerebral brain volume | COPRelativeLongLatency | -5.2×10^-2^ | 1.7×10^-2^ | 2.2×10^-3^ |
| Total Cerebral brain volume | COMDrawingEfficiency | 5.1×10^-2^ | 1.7×10^-2^ | 2.3×10^-3^ |
| Total Cerebral brain volume | COMSpatialReasoning | 5.1×10^-2^ | 1.7×10^-2^ | 2.7×10^-3^ |
| Total Cerebral brain volume | COMComponentPlacement_s | 5.0×10^-2^ | 1.7×10^-2^ | 2.7×10^-3^ |
| Total Cerebral brain volume | COPLongLatencyCount | -5.2×10^-2^ | 1.7×10^-2^ | 3.0×10^-3^ |
| Total Cerebral brain volume | COMSimpleMotor_s | 4.9×10^-2^ | 1.6×10^-2^ | 3.3×10^-3^ |
| Total Cerebral brain volume | COPLongestLatency | -4.9×10^-2^ | 1.7×10^-2^ | 3.5×10^-3^ |
| Total Cerebral brain volume | COPLatencyVariability | -4.8×10^-2^ | 1.7×10^-2^ | 4.3×10^-3^ |
| Total Cerebral brain volume | COMSpatialReasoning_s | 4.8×10^-2^ | 1.7×10^-2^ | 4.6×10^-3^ |
| Total Cerebral brain volume | COMInformationProcessing | 4.7×10^-2^ | 1.7×10^-2^ | 5.0×10^-3^ |
| Total Cerebral brain volume | COPInformationProcessing_s | 4.6×10^-2^ | 1.7×10^-2^ | 5.4×10^-3^ |
| Total Cerebral brain volume | COPComponentPlacement_s | 4.5×10^-2^ | 1.6×10^-2^ | 5.5×10^-3^ |
| Total Cerebral brain volume | COPRelativeLongLatency_s | 4.5×10^-2^ | 1.7×10^-2^ | 6.8×10^-3^ |
| Total Cerebral brain volume | COMLatencyVariability | -4.4×10^-2^ | 1.7×10^-2^ | 7.8×10^-3^ |
| Total Cerebral brain volume | COMDrawingEfficiency_s | 4.4×10^-2^ | 1.7×10^-2^ | 8.0×10^-3^ |
| Total Cerebral brain volume | COPLongestLatency_s | 4.3×10^-2^ | 1.6×10^-2^ | 8.9×10^-3^ |
| Total Cerebral brain volume | COMTotalTime_s | 4.3×10^-2^ | 1.6×10^-2^ | 9.4×10^-3^ |
| Total Cerebral brain volume | COPLatencyVariability_s | 4.2×10^-2^ | 1.6×10^-2^ | 1.0×10^-2^ |
| Total Cerebral brain volume | COMNoise | -4.2×10^-2^ | 1.7×10^-2^ | 1.1×10^-2^ |
| Total Cerebral brain volume | COMRelativeLongLatency | -4.2×10^-2^ | 1.7×10^-2^ | 1.2×10^-2^ |
| Total Cerebral brain volume | COMInformationProcessing_s | 4.0×10^-2^ | 1.6×10^-2^ | 1.6×10^-2^ |
| Total Cerebral brain volume | COPNoise | -3.9×10^-2^ | 1.6×10^-2^ | 1.7×10^-2^ |
| Total Cerebral brain volume | COMNoise_s | 3.8×10^-2^ | 1.6×10^-2^ | 1.9×10^-2^ |
| Total Cerebral brain volume | COMLatencyVariability_s | 3.7×10^-2^ | 1.6×10^-2^ | 2.4×10^-2^ |
| Total Cerebral brain volume | COMLongLatencyCount | -3.8×10^-2^ | 1.7×10^-2^ | 2.6×10^-2^ |
| Total Cerebral brain volume | COMRelativeLongLatency_s | 3.4×10^-2^ | 1.6×10^-2^ | 3.7×10^-2^ |
| Total Cerebral brain volume | COMLongestLatency | -3.4×10^-2^ | 1.6×10^-2^ | 4.1×10^-2^ |
| Total Cerebral brain volume | COPStrokeCountConformity | -3.5×10^-2^ | 1.8×10^-2^ | 4.8×10^-2^ |
| Total Cerebral brain volume | COPLongLatencyCount_s | 3.1×10^-2^ | 1.7×10^-2^ | 6.2×10^-2^ |
| Total Cerebral brain volume | COMInkLength_s | 3.0×10^-2^ | 1.7×10^-2^ | 7.2×10^-2^ |
| Total Cerebral brain volume | COMTerminationSpeed | 2.9×10^-2^ | 1.6×10^-2^ | 7.9×10^-2^ |
| Total Cerebral brain volume | COMLongestLatency_s | 2.8×10^-2^ | 1.6×10^-2^ | 8.8×10^-2^ |
| Total Cerebral brain volume | COMDrawingSize_s | 2.8×10^-2^ | 1.7×10^-2^ | 9.1×10^-2^ |
| Total Cerebral brain volume | COMInkLength | 2.8×10^-2^ | 1.7×10^-2^ | 9.3×10^-2^ |
| Total Cerebral brain volume | COMStrokeCountConformity | -2.6×10^-2^ | 1.7×10^-2^ | 1.2×10^-1^ |
| Total Cerebral brain volume | COMDrawingSize | 2.4×10^-2^ | 1.7×10^-2^ | 1.4×10^-1^ |
| Total Cerebral brain volume | COPVerticalSpatialPlacement_s | 2.4×10^-2^ | 1.6×10^-2^ | 1.4×10^-1^ |
| Total Cerebral brain volume | COMTerminationSpeed_s | 2.4×10^-2^ | 1.6×10^-2^ | 1.5×10^-1^ |
| Total Cerebral brain volume | COPDrawingSize_s | 2.2×10^-2^ | 1.6×10^-2^ | 1.7×10^-1^ |
| Total Cerebral brain volume | COPHorizontalSpatialPlacement | 2.1×10^-2^ | 1.6×10^-2^ | 1.8×10^-1^ |
| Total Cerebral brain volume | COPHorizontalSpatialPlacement_s | -2.1×10^-2^ | 1.6×10^-2^ | 1.9×10^-1^ |
| Total Cerebral brain volume | COMLongLatencyCount_s | 2.2×10^-2^ | 1.7×10^-2^ | 1.9×10^-1^ |
| Total Cerebral brain volume | COMAverageSpeed | 2.1×10^-2^ | 1.6×10^-2^ | 2.0×10^-1^ |
| Total Cerebral brain volume | COPVerticalSpatialPlacement | -2.1×10^-2^ | 1.6×10^-2^ | 2.0×10^-1^ |
| Total Cerebral brain volume | COPDrawingSize | 2.0×10^-2^ | 1.6×10^-2^ | 2.3×10^-1^ |
| Total Cerebral brain volume | COMInitiationSpeed | 1.9×10^-2^ | 1.6×10^-2^ | 2.3×10^-1^ |
| Total Cerebral brain volume | COMClockfaceCircularity | -1.9×10^-2^ | 1.7×10^-2^ | 2.6×10^-1^ |
| Total Cerebral brain volume | COMClockfaceCircularity_s | 1.8×10^-2^ | 1.6×10^-2^ | 2.7×10^-1^ |
| Total Cerebral brain volume | COPTerminationSpeed | 1.7×10^-2^ | 1.6×10^-2^ | 3.0×10^-1^ |
| Total Cerebral brain volume | COMInitiationSpeed_s | 1.7×10^-2^ | 1.6×10^-2^ | 3.0×10^-1^ |
| Total Cerebral brain volume | COMAverageSpeed_s | 1.6×10^-2^ | 1.6×10^-2^ | 3.2×10^-1^ |
| Total Cerebral brain volume | COPAverageSpeed | 1.5×10^-2^ | 1.6×10^-2^ | 3.4×10^-1^ |
| Total Cerebral brain volume | COPTerminationSpeed_s | 1.2×10^-2^ | 1.6×10^-2^ | 4.6×10^-1^ |
| Total Cerebral brain volume | COPInitiationSpeed | 1.0×10^-2^ | 1.6×10^-2^ | 5.2×10^-1^ |
| Total Cerebral brain volume | COMPercentInkTime_s | -9.6×10^-3^ | 1.6×10^-2^ | 5.6×10^-1^ |
| Total Cerebral brain volume | COMPercentThinkTime_s | 9.3×10^-3^ | 1.6×10^-2^ | 5.7×10^-1^ |
| Total Cerebral brain volume | COPAverageSpeed_s | 9.1×10^-3^ | 1.6×10^-2^ | 5.7×10^-1^ |
| Total Cerebral brain volume | COMVerticalSpatialPlacement | 9.1×10^-3^ | 1.6×10^-2^ | 5.7×10^-1^ |
| Total Cerebral brain volume | COMPercentThinkTime | -8.4×10^-3^ | 1.6×10^-2^ | 6.1×10^-1^ |
| Total Cerebral brain volume | COMPercentInkTime | 8.1×10^-3^ | 1.6×10^-2^ | 6.2×10^-1^ |
| Total Cerebral brain volume | COPClockfaceCircularity_s | 7.7×10^-3^ | 1.6×10^-2^ | 6.3×10^-1^ |
| Total Cerebral brain volume | COMHorizontalSpatialPlacement | 7.4×10^-3^ | 1.6×10^-2^ | 6.5×10^-1^ |
| Total Cerebral brain volume | COPInkLength_s | 6.7×10^-3^ | 1.6×10^-2^ | 6.8×10^-1^ |
| Total Cerebral brain volume | COPStrokeCountConformity_s | 6.1×10^-3^ | 1.7×10^-2^ | 7.2×10^-1^ |
| Total Cerebral brain volume | COPMaxSpeed_s | -5.1×10^-3^ | 1.6×10^-2^ | 7.5×10^-1^ |
| Total Cerebral brain volume | COPInitiationSpeed_s | 4.9×10^-3^ | 1.6×10^-2^ | 7.6×10^-1^ |
| Total Cerebral brain volume | COMMaxSpeed | 5.0×10^-3^ | 1.6×10^-2^ | 7.6×10^-1^ |
| Total Cerebral brain volume | COPNoise_s | 4.5×10^-3^ | 1.7×10^-2^ | 7.9×10^-1^ |
| Total Cerebral brain volume | COPPercentThinkTime | 3.4×10^-3^ | 1.7×10^-2^ | 8.4×10^-1^ |
| Total Cerebral brain volume | COPClockfaceCircularity | -3.2×10^-3^ | 1.6×10^-2^ | 8.4×10^-1^ |
| Total Cerebral brain volume | COPPercentThinkTime_s | 3.1×10^-3^ | 1.7×10^-2^ | 8.5×10^-1^ |
| Total Cerebral brain volume | COMVerticalSpatialPlacement_s | -2.7×10^-3^ | 1.6×10^-2^ | 8.7×10^-1^ |
| Total Cerebral brain volume | COMHorizontalSpatialPlacement_s | -2.6×10^-3^ | 1.6×10^-2^ | 8.8×10^-1^ |
| Total Cerebral brain volume | COMMaxSpeed_s | 2.0×10^-3^ | 1.6×10^-2^ | 9.0×10^-1^ |
| Total Cerebral brain volume | COPInkLength | 1.7×10^-3^ | 1.6×10^-2^ | 9.2×10^-1^ |
| Total Cerebral brain volume | COPPercentInkTime | -1.1×10^-3^ | 1.7×10^-2^ | 9.5×10^-1^ |
| Total Cerebral brain volume | COPPercentInkTime_s | -9.0×10^-4^ | 1.7×10^-2^ | 9.6×10^-1^ |
| Total Cerebral brain volume | COMStrokeCountConformity_s | 6.3×10^-4^ | 1.7×10^-2^ | 9.7×10^-1^ |
| Total Cerebral brain volume | COPMaxSpeed | 3.0×10^-5^ | 1.6×10^-2^ | 1.0×10^0^ |
|  |  |  |  |  |
| Cerebral white matter volume | DCTScore | 9.3×10^-2^ | 2.3×10^-2^ | **7.3×10^-5^** |
| Cerebral white matter volume | COPNoise | -7.8×10^-2^ | 2.2×10^-2^ | **4.5×10^-4^** |
| Cerebral white matter volume | COPSpatialReasoning | 7.7×10^-2^ | 2.2×10^-2^ | 5.9×10^-4^ |
| Cerebral white matter volume | COPDrawingEfficiency | 7.6×10^-2^ | 2.3×10^-2^ | 7.9×10^-4^ |
| Cerebral white matter volume | COPTotalTime | -7.5×10^-2^ | 2.3×10^-2^ | 1.1×10^-3^ |
| Cerebral white matter volume | COPSpatialReasoning_s | 6.7×10^-2^ | 2.2×10^-2^ | 2.2×10^-3^ |
| Cerebral white matter volume | COPDrawingProcessEfficiency | 7.0×10^-2^ | 2.3×10^-2^ | 2.2×10^-3^ |
| Cerebral white matter volume | COPAverageLatency | -6.6×10^-2^ | 2.3×10^-2^ | 3.6×10^-3^ |
| Cerebral white matter volume | COPDrawingEfficiency_s | 6.3×10^-2^ | 2.2×10^-2^ | 5.1×10^-3^ |
| Cerebral white matter volume | COMAverageLatency | -5.9×10^-2^ | 2.3×10^-2^ | 9.6×10^-3^ |
| Cerebral white matter volume | COPComponentPlacement | -5.7×10^-2^ | 2.2×10^-2^ | 1.1×10^-2^ |
| Cerebral white matter volume | COPDrawingProcessEfficiency_s | 5.7×10^-2^ | 2.3×10^-2^ | 1.1×10^-2^ |
| Cerebral white matter volume | COPOscillatoryMotion | -5.7×10^-2^ | 2.3×10^-2^ | 1.1×10^-2^ |
| Cerebral white matter volume | COMDrawingProcessEfficiency | 5.7×10^-2^ | 2.3×10^-2^ | 1.2×10^-2^ |
| Cerebral white matter volume | COPNoise_s | 5.5×10^-2^ | 2.3×10^-2^ | 1.5×10^-2^ |
| Cerebral white matter volume | COPLongLatencyCount | -5.7×10^-2^ | 2.3×10^-2^ | 1.5×10^-2^ |
| Cerebral white matter volume | COPTotalTime_s | 5.2×10^-2^ | 2.2×10^-2^ | 1.8×10^-2^ |
| Cerebral white matter volume | COPStrokeCountConformity | -5.6×10^-2^ | 2.4×10^-2^ | 1.8×10^-2^ |
| Cerebral white matter volume | COPLongestLatency | -5.3×10^-2^ | 2.2×10^-2^ | 1.9×10^-2^ |
| Cerebral white matter volume | COPInformationProcessing | 5.3×10^-2^ | 2.3×10^-2^ | 2.0×10^-2^ |
| Cerebral white matter volume | COPAverageLatency_s | 5.1×10^-2^ | 2.2×10^-2^ | 2.4×10^-2^ |
| Cerebral white matter volume | COPRelativeLongLatency | -5.1×10^-2^ | 2.3×10^-2^ | 2.7×10^-2^ |
| Cerebral white matter volume | COPComponentPlacement_s | 4.8×10^-2^ | 2.2×10^-2^ | 2.7×10^-2^ |
| Cerebral white matter volume | COMDrawingEfficiency | 4.9×10^-2^ | 2.3×10^-2^ | 3.0×10^-2^ |
| Cerebral white matter volume | COPLatencyVariability | -4.8×10^-2^ | 2.3×10^-2^ | 3.4×10^-2^ |
| Cerebral white matter volume | COMDrawingProcessEfficiency_s | 4.7×10^-2^ | 2.2×10^-2^ | 3.5×10^-2^ |
| Cerebral white matter volume | COMAverageLatency_s | 4.7×10^-2^ | 2.2×10^-2^ | 3.6×10^-2^ |
| Cerebral white matter volume | COPVerticalSpatialPlacement | -4.5×10^-2^ | 2.2×10^-2^ | 3.8×10^-2^ |
| Cerebral white matter volume | COMComponentPlacement | -4.6×10^-2^ | 2.3×10^-2^ | 4.1×10^-2^ |
| Cerebral white matter volume | COMSpatialReasoning | 4.6×10^-2^ | 2.3×10^-2^ | 4.2×10^-2^ |
| Cerebral white matter volume | COPLongestLatency_s | 4.3×10^-2^ | 2.2×10^-2^ | 5.1×10^-2^ |
| Cerebral white matter volume | COMTotalTime | -4.3×10^-2^ | 2.3×10^-2^ | 5.5×10^-2^ |
| Cerebral white matter volume | COPVerticalSpatialPlacement_s | 4.1×10^-2^ | 2.2×10^-2^ | 5.7×10^-2^ |
| Cerebral white matter volume | COPInformationProcessing_s | 4.2×10^-2^ | 2.2×10^-2^ | 5.8×10^-2^ |
| Cerebral white matter volume | COMOscillatoryMotion | -4.4×10^-2^ | 2.3×10^-2^ | 5.9×10^-2^ |
| Cerebral white matter volume | COMDrawingEfficiency_s | 4.0×10^-2^ | 2.2×10^-2^ | 7.5×10^-2^ |
| Cerebral white matter volume | COPLatencyVariability_s | 3.9×10^-2^ | 2.2×10^-2^ | 7.6×10^-2^ |
| Cerebral white matter volume | COMLatencyVariability | -3.9×10^-2^ | 2.2×10^-2^ | 8.2×10^-2^ |
| Cerebral white matter volume | COMSpatialReasoning_s | 3.9×10^-2^ | 2.3×10^-2^ | 8.5×10^-2^ |
| Cerebral white matter volume | COPRelativeLongLatency_s | 3.9×10^-2^ | 2.2×10^-2^ | 8.5×10^-2^ |
| Cerebral white matter volume | COMComponentPlacement_s | 3.8×10^-2^ | 2.3×10^-2^ | 8.9×10^-2^ |
| Cerebral white matter volume | COPSimpleMotor | 3.8×10^-2^ | 2.2×10^-2^ | 9.1×10^-2^ |
| Cerebral white matter volume | COMInformationProcessing | 3.7×10^-2^ | 2.2×10^-2^ | 9.5×10^-2^ |
| Cerebral white matter volume | COPOscillatoryMotion_s | 3.6×10^-2^ | 2.2×10^-2^ | 9.8×10^-2^ |
| Cerebral white matter volume | COMRelativeLongLatency | -3.6×10^-2^ | 2.2×10^-2^ | 1.0×10^-1^ |
| Cerebral white matter volume | COMDrawingSize_s | 3.3×10^-2^ | 2.2×10^-2^ | 1.3×10^-1^ |
| Cerebral white matter volume | COMSimpleMotor | 3.3×10^-2^ | 2.3×10^-2^ | 1.5×10^-1^ |
| Cerebral white matter volume | COMTotalTime_s | 3.1×10^-2^ | 2.2×10^-2^ | 1.6×10^-1^ |
| Cerebral white matter volume | COMLatencyVariability_s | 3.0×10^-2^ | 2.2×10^-2^ | 1.7×10^-1^ |
| Cerebral white matter volume | COMDrawingSize | 2.9×10^-2^ | 2.2×10^-2^ | 1.9×10^-1^ |
| Cerebral white matter volume | COMInformationProcessing_s | 2.9×10^-2^ | 2.2×10^-2^ | 1.9×10^-1^ |
| Cerebral white matter volume | COPDrawingSize_s | 2.7×10^-2^ | 2.2×10^-2^ | 2.1×10^-1^ |
| Cerebral white matter volume | COMRelativeLongLatency_s | 2.7×10^-2^ | 2.2×10^-2^ | 2.1×10^-1^ |
| Cerebral white matter volume | COMLongestLatency | -2.7×10^-2^ | 2.2×10^-2^ | 2.3×10^-1^ |
| Cerebral white matter volume | COMLongLatencyCount | -2.6×10^-2^ | 2.3×10^-2^ | 2.5×10^-1^ |
| Cerebral white matter volume | COMNoise_s | 2.4×10^-2^ | 2.2×10^-2^ | 2.7×10^-1^ |
| Cerebral white matter volume | COPDrawingSize | 2.4×10^-2^ | 2.2×10^-2^ | 2.7×10^-1^ |
| Cerebral white matter volume | COMHorizontalSpatialPlacement | -2.3×10^-2^ | 2.2×10^-2^ | 2.8×10^-1^ |
| Cerebral white matter volume | COMNoise | -2.3×10^-2^ | 2.2×10^-2^ | 3.0×10^-1^ |
| Cerebral white matter volume | COMOscillatoryMotion_s | 2.3×10^-2^ | 2.3×10^-2^ | 3.1×10^-1^ |
| Cerebral white matter volume | COPSimpleMotor_s | 2.1×10^-2^ | 2.2×10^-2^ | 3.4×10^-1^ |
| Cerebral white matter volume | COMVerticalSpatialPlacement | -2.1×10^-2^ | 2.2×10^-2^ | 3.4×10^-1^ |
| Cerebral white matter volume | COMLongestLatency_s | 2.0×10^-2^ | 2.2×10^-2^ | 3.5×10^-1^ |
| Cerebral white matter volume | COMSimpleMotor_s | 2.0×10^-2^ | 2.2×10^-2^ | 3.8×10^-1^ |
| Cerebral white matter volume | COMPercentThinkTime_s | 1.9×10^-2^ | 2.2×10^-2^ | 3.8×10^-1^ |
| Cerebral white matter volume | COMPercentInkTime_s | -1.9×10^-2^ | 2.2×10^-2^ | 3.8×10^-1^ |
| Cerebral white matter volume | COMInkLength_s | 1.9×10^-2^ | 2.2×10^-2^ | 3.8×10^-1^ |
| Cerebral white matter volume | COMPercentInkTime | 1.9×10^-2^ | 2.2×10^-2^ | 4.0×10^-1^ |
| Cerebral white matter volume | COMPercentThinkTime | -1.9×10^-2^ | 2.2×10^-2^ | 4.0×10^-1^ |
| Cerebral white matter volume | COMHorizontalSpatialPlacement_s | 1.9×10^-2^ | 2.2×10^-2^ | 4.0×10^-1^ |
| Cerebral white matter volume | COPStrokeCountConformity_s | 1.9×10^-2^ | 2.3×10^-2^ | 4.1×10^-1^ |
| Cerebral white matter volume | COMInkLength | 1.8×10^-2^ | 2.2×10^-2^ | 4.1×10^-1^ |
| Cerebral white matter volume | COMVerticalSpatialPlacement_s | 1.7×10^-2^ | 2.2×10^-2^ | 4.2×10^-1^ |
| Cerebral white matter volume | COMTerminationSpeed | 1.8×10^-2^ | 2.2×10^-2^ | 4.2×10^-1^ |
| Cerebral white matter volume | COPLongLatencyCount_s | 1.7×10^-2^ | 2.3×10^-2^ | 4.4×10^-1^ |
| Cerebral white matter volume | COPHorizontalSpatialPlacement_s | -1.7×10^-2^ | 2.2×10^-2^ | 4.4×10^-1^ |
| Cerebral white matter volume | COMStrokeCountConformity_s | -1.6×10^-2^ | 2.2×10^-2^ | 4.8×10^-1^ |
| Cerebral white matter volume | COPInitiationSpeed_s | -1.4×10^-2^ | 2.1×10^-2^ | 5.2×10^-1^ |
| Cerebral white matter volume | COPMaxSpeed_s | -1.4×10^-2^ | 2.2×10^-2^ | 5.3×10^-1^ |
| Cerebral white matter volume | COMClockfaceCircularity | -1.3×10^-2^ | 2.2×10^-2^ | 5.5×10^-1^ |
| Cerebral white matter volume | COPPercentInkTime_s | -1.2×10^-2^ | 2.2×10^-2^ | 5.9×10^-1^ |
| Cerebral white matter volume | COMStrokeCountConformity | -1.2×10^-2^ | 2.3×10^-2^ | 5.9×10^-1^ |
| Cerebral white matter volume | COMTerminationSpeed_s | 1.2×10^-2^ | 2.2×10^-2^ | 6.0×10^-1^ |
| Cerebral white matter volume | COPPercentThinkTime_s | 1.1×10^-2^ | 2.2×10^-2^ | 6.1×10^-1^ |
| Cerebral white matter volume | COPHorizontalSpatialPlacement | 9.9×10^-3^ | 2.2×10^-2^ | 6.5×10^-1^ |
| Cerebral white matter volume | COPInitiationSpeed | -8.1×10^-3^ | 2.2×10^-2^ | 7.1×10^-1^ |
| Cerebral white matter volume | COPInkLength_s | 8.1×10^-3^ | 2.2×10^-2^ | 7.1×10^-1^ |
| Cerebral white matter volume | COPClockfaceCircularity | -8.0×10^-3^ | 2.2×10^-2^ | 7.2×10^-1^ |
| Cerebral white matter volume | COPClockfaceCircularity_s | 7.8×10^-3^ | 2.2×10^-2^ | 7.2×10^-1^ |
| Cerebral white matter volume | COPMaxSpeed | -7.7×10^-3^ | 2.2×10^-2^ | 7.2×10^-1^ |
| Cerebral white matter volume | COPTerminationSpeed | 7.1×10^-3^ | 2.2×10^-2^ | 7.4×10^-1^ |
| Cerebral white matter volume | COPPercentThinkTime | -7.2×10^-3^ | 2.2×10^-2^ | 7.5×10^-1^ |
| Cerebral white matter volume | COPPercentInkTime | 6.8×10^-3^ | 2.2×10^-2^ | 7.6×10^-1^ |
| Cerebral white matter volume | COMAverageSpeed | 6.3×10^-3^ | 2.2×10^-2^ | 7.8×10^-1^ |
| Cerebral white matter volume | COMMaxSpeed | 4.5×10^-3^ | 2.2×10^-2^ | 8.4×10^-1^ |
| Cerebral white matter volume | COPAverageSpeed_s | -4.2×10^-3^ | 2.2×10^-2^ | 8.5×10^-1^ |
| Cerebral white matter volume | COMClockfaceCircularity_s | 4.2×10^-3^ | 2.2×10^-2^ | 8.5×10^-1^ |
| Cerebral white matter volume | COMInitiationSpeed_s | -3.6×10^-3^ | 2.2×10^-2^ | 8.7×10^-1^ |
| Cerebral white matter volume | COPAverageSpeed | 3.5×10^-3^ | 2.2×10^-2^ | 8.7×10^-1^ |
| Cerebral white matter volume | COMLongLatencyCount_s | 2.8×10^-3^ | 2.3×10^-2^ | 9.0×10^-1^ |
| Cerebral white matter volume | COPInkLength | 1.2×10^-3^ | 2.2×10^-2^ | 9.6×10^-1^ |
| Cerebral white matter volume | COPTerminationSpeed_s | 7.9×10^-4^ | 2.2×10^-2^ | 9.7×10^-1^ |
| Cerebral white matter volume | COMMaxSpeed_s | 5.8×10^-4^ | 2.2×10^-2^ | 9.8×10^-1^ |
| Cerebral white matter volume | COMInitiationSpeed | 3.6×10^-4^ | 2.2×10^-2^ | 9.9×10^-1^ |
| Cerebral white matter volume | COMAverageSpeed_s | -3.0×10^-4^ | 2.2×10^-2^ | 9.9×10^-1^ |
|  |  |  |  |  |
| Cerebral gray matter volume | COPOscillatoryMotion_s | 8.3×10^-2^ | 1.8×10^-2^ | **5.6×10^-6^** |
| Cerebral gray matter volume | COPOscillatoryMotion | -7.6×10^-2^ | 1.9×10^-2^ | **5.6×10^-5^** |
| Cerebral gray matter volume | COPSimpleMotor | 7.4×10^-2^ | 1.9×10^-2^ | **7.7×10^-5^** |
| Cerebral gray matter volume | COPSimpleMotor_s | 6.5×10^-2^ | 1.8×10^-2^ | **3.5×10^-4^** |
| Cerebral gray matter volume | COMOscillatoryMotion_s | 6.4×10^-2^ | 1.9×10^-2^ | 7.2×10^-4^ |
| Cerebral gray matter volume | COMOscillatoryMotion | -6.6×10^-2^ | 2.0×10^-2^ | 7.4×10^-4^ |
| Cerebral gray matter volume | COPNoise_s | -5.8×10^-2^ | 1.9×10^-2^ | 2.2×10^-3^ |
| Cerebral gray matter volume | DCTScore | 5.4×10^-2^ | 2.0×10^-2^ | 6.7×10^-3^ |
| Cerebral gray matter volume | COMSimpleMotor | 5.2×10^-2^ | 1.9×10^-2^ | 6.8×10^-3^ |
| Cerebral gray matter volume | COMDrawingProcessEfficiency | 4.7×10^-2^ | 1.9×10^-2^ | 1.4×10^-2^ |
| Cerebral gray matter volume | COMSimpleMotor_s | 4.4×10^-2^ | 1.9×10^-2^ | 2.0×10^-2^ |
| Cerebral gray matter volume | COMTotalTime | -4.1×10^-2^ | 1.9×10^-2^ | 3.2×10^-2^ |
| Cerebral gray matter volume | COMDrawingProcessEfficiency_s | 3.9×10^-2^ | 1.9×10^-2^ | 3.6×10^-2^ |
| Cerebral gray matter volume | COMVerticalSpatialPlacement | 3.6×10^-2^ | 1.8×10^-2^ | 4.6×10^-2^ |
| Cerebral gray matter volume | COMNoise | -3.7×10^-2^ | 1.9×10^-2^ | 4.8×10^-2^ |
| Cerebral gray matter volume | COPDrawingProcessEfficiency | 3.7×10^-2^ | 1.9×10^-2^ | 5.4×10^-2^ |
| Cerebral gray matter volume | COMHorizontalSpatialPlacement | 3.4×10^-2^ | 1.8×10^-2^ | 6.0×10^-2^ |
| Cerebral gray matter volume | COPInitiationSpeed | 3.4×10^-2^ | 1.8×10^-2^ | 6.2×10^-2^ |
| Cerebral gray matter volume | COMTotalTime_s | 3.5×10^-2^ | 1.9×10^-2^ | 6.4×10^-2^ |
| Cerebral gray matter volume | COMInitiationSpeed | 3.4×10^-2^ | 1.8×10^-2^ | 6.4×10^-2^ |
| Cerebral gray matter volume | COMNoise_s | 3.2×10^-2^ | 1.8×10^-2^ | 7.5×10^-2^ |
| Cerebral gray matter volume | COMInitiationSpeed_s | 3.2×10^-2^ | 1.8×10^-2^ | 7.9×10^-2^ |
| Cerebral gray matter volume | COMInformationProcessing | 3.3×10^-2^ | 1.9×10^-2^ | 8.0×10^-2^ |
| Cerebral gray matter volume | COMComponentPlacement | -3.3×10^-2^ | 1.9×10^-2^ | 8.1×10^-2^ |
| Cerebral gray matter volume | COMComponentPlacement_s | 3.2×10^-2^ | 1.9×10^-2^ | 9.5×10^-2^ |
| Cerebral gray matter volume | COMAverageLatency | -3.2×10^-2^ | 1.9×10^-2^ | 9.8×10^-2^ |
| Cerebral gray matter volume | COPVerticalSpatialPlacement | 3.0×10^-2^ | 1.8×10^-2^ | 1.0×10^-1^ |
| Cerebral gray matter volume | COPDrawingProcessEfficiency_s | 3.1×10^-2^ | 1.9×10^-2^ | 1.0×10^-1^ |
| Cerebral gray matter volume | COPHorizontalSpatialPlacement | 2.9×10^-2^ | 1.8×10^-2^ | 1.1×10^-1^ |
| Cerebral gray matter volume | COPInformationProcessing | 3.1×10^-2^ | 1.9×10^-2^ | 1.1×10^-1^ |
| Cerebral gray matter volume | COPInitiationSpeed_s | 2.9×10^-2^ | 1.8×10^-2^ | 1.1×10^-1^ |
| Cerebral gray matter volume | COPNoise | 2.9×10^-2^ | 1.9×10^-2^ | 1.2×10^-1^ |
| Cerebral gray matter volume | COMAverageSpeed | 2.8×10^-2^ | 1.8×10^-2^ | 1.3×10^-1^ |
| Cerebral gray matter volume | COMInformationProcessing_s | 2.8×10^-2^ | 1.9×10^-2^ | 1.3×10^-1^ |
| Cerebral gray matter volume | COPAverageSpeed | 2.8×10^-2^ | 1.8×10^-2^ | 1.3×10^-1^ |
| Cerebral gray matter volume | COMRelativeLongLatency | -2.8×10^-2^ | 1.9×10^-2^ | 1.3×10^-1^ |
| Cerebral gray matter volume | COPDrawingEfficiency | 2.8×10^-2^ | 1.9×10^-2^ | 1.5×10^-1^ |
| Cerebral gray matter volume | COMDrawingEfficiency | 2.8×10^-2^ | 1.9×10^-2^ | 1.5×10^-1^ |
| Cerebral gray matter volume | COMLatencyVariability | -2.7×10^-2^ | 1.9×10^-2^ | 1.5×10^-1^ |
| Cerebral gray matter volume | COMStrokeCountConformity | -2.7×10^-2^ | 1.9×10^-2^ | 1.5×10^-1^ |
| Cerebral gray matter volume | COPLongLatencyCount_s | 2.7×10^-2^ | 1.9×10^-2^ | 1.5×10^-1^ |
| Cerebral gray matter volume | COPTotalTime_s | 2.6×10^-2^ | 1.9×10^-2^ | 1.6×10^-1^ |
| Cerebral gray matter volume | COMSpatialReasoning_s | 2.6×10^-2^ | 1.9×10^-2^ | 1.6×10^-1^ |
| Cerebral gray matter volume | COPComponentPlacement | -2.6×10^-2^ | 1.9×10^-2^ | 1.7×10^-1^ |
| Cerebral gray matter volume | COMLongLatencyCount | -2.6×10^-2^ | 1.9×10^-2^ | 1.7×10^-1^ |
| Cerebral gray matter volume | COMAverageSpeed_s | 2.5×10^-2^ | 1.8×10^-2^ | 1.7×10^-1^ |
| Cerebral gray matter volume | COMLongLatencyCount_s | 2.6×10^-2^ | 1.9×10^-2^ | 1.8×10^-1^ |
| Cerebral gray matter volume | COMAverageLatency_s | 2.5×10^-2^ | 1.9×10^-2^ | 1.8×10^-1^ |
| Cerebral gray matter volume | COMVerticalSpatialPlacement_s | -2.4×10^-2^ | 1.8×10^-2^ | 1.8×10^-1^ |
| Cerebral gray matter volume | COPTerminationSpeed | 2.5×10^-2^ | 1.8×10^-2^ | 1.8×10^-1^ |
| Cerebral gray matter volume | COMLongestLatency | -2.5×10^-2^ | 1.9×10^-2^ | 1.8×10^-1^ |
| Cerebral gray matter volume | COMSpatialReasoning | 2.5×10^-2^ | 1.9×10^-2^ | 1.8×10^-1^ |
| Cerebral gray matter volume | COPAverageLatency_s | 2.5×10^-2^ | 1.9×10^-2^ | 1.9×10^-1^ |
| Cerebral gray matter volume | COPRelativeLongLatency_s | 2.5×10^-2^ | 1.9×10^-2^ | 1.9×10^-1^ |
| Cerebral gray matter volume | COPInformationProcessing_s | 2.5×10^-2^ | 1.9×10^-2^ | 1.9×10^-1^ |
| Cerebral gray matter volume | COMHorizontalSpatialPlacement_s | -2.4×10^-2^ | 1.9×10^-2^ | 1.9×10^-1^ |
| Cerebral gray matter volume | COMDrawingEfficiency_s | 2.4×10^-2^ | 1.9×10^-2^ | 2.0×10^-1^ |
| Cerebral gray matter volume | COMRelativeLongLatency_s | 2.3×10^-2^ | 1.8×10^-2^ | 2.1×10^-1^ |
| Cerebral gray matter volume | COPAverageSpeed_s | 2.3×10^-2^ | 1.8×10^-2^ | 2.1×10^-1^ |
| Cerebral gray matter volume | COPDrawingEfficiency_s | 2.3×10^-2^ | 1.9×10^-2^ | 2.3×10^-1^ |
| Cerebral gray matter volume | COPPercentThinkTime | 2.3×10^-2^ | 1.9×10^-2^ | 2.3×10^-1^ |
| Cerebral gray matter volume | COMLatencyVariability_s | 2.2×10^-2^ | 1.9×10^-2^ | 2.3×10^-1^ |
| Cerebral gray matter volume | COPLatencyVariability_s | 2.2×10^-2^ | 1.9×10^-2^ | 2.4×10^-1^ |
| Cerebral gray matter volume | COMTerminationSpeed | 2.2×10^-2^ | 1.9×10^-2^ | 2.4×10^-1^ |
| Cerebral gray matter volume | COPComponentPlacement_s | 2.1×10^-2^ | 1.8×10^-2^ | 2.5×10^-1^ |
| Cerebral gray matter volume | COPTerminationSpeed_s | 2.1×10^-2^ | 1.8×10^-2^ | 2.5×10^-1^ |
| Cerebral gray matter volume | COPTotalTime | -2.1×10^-2^ | 1.9×10^-2^ | 2.6×10^-1^ |
| Cerebral gray matter volume | COPLongestLatency_s | 2.0×10^-2^ | 1.9×10^-2^ | 2.7×10^-1^ |
| Cerebral gray matter volume | COMLongestLatency_s | 2.0×10^-2^ | 1.8×10^-2^ | 2.7×10^-1^ |
| Cerebral gray matter volume | COPMaxSpeed | 2.0×10^-2^ | 1.8×10^-2^ | 2.8×10^-1^ |
| Cerebral gray matter volume | COPPercentInkTime_s | 2.0×10^-2^ | 1.9×10^-2^ | 2.9×10^-1^ |
| Cerebral gray matter volume | COPDrawingSize_s | 1.9×10^-2^ | 1.8×10^-2^ | 3.0×10^-1^ |
| Cerebral gray matter volume | COMTerminationSpeed_s | 1.8×10^-2^ | 1.9×10^-2^ | 3.3×10^-1^ |
| Cerebral gray matter volume | COPDrawingSize | 1.8×10^-2^ | 1.8×10^-2^ | 3.4×10^-1^ |
| Cerebral gray matter volume | COPHorizontalSpatialPlacement_s | -1.7×10^-2^ | 1.8×10^-2^ | 3.6×10^-1^ |
| Cerebral gray matter volume | COPAverageLatency | -1.8×10^-2^ | 1.9×10^-2^ | 3.6×10^-1^ |
| Cerebral gray matter volume | COPStrokeCountConformity | 1.8×10^-2^ | 2.0×10^-2^ | 3.6×10^-1^ |
| Cerebral gray matter volume | COPVerticalSpatialPlacement_s | -1.6×10^-2^ | 1.8×10^-2^ | 3.8×10^-1^ |
| Cerebral gray matter volume | COMInkLength_s | 1.7×10^-2^ | 1.9×10^-2^ | 3.8×10^-1^ |
| Cerebral gray matter volume | COMStrokeCountConformity_s | 1.6×10^-2^ | 1.9×10^-2^ | 3.9×10^-1^ |
| Cerebral gray matter volume | COPRelativeLongLatency | -1.6×10^-2^ | 1.9×10^-2^ | 3.9×10^-1^ |
| Cerebral gray matter volume | COPLatencyVariability | -1.6×10^-2^ | 1.9×10^-2^ | 4.0×10^-1^ |
| Cerebral gray matter volume | COPInkLength_s | 1.5×10^-2^ | 1.8×10^-2^ | 4.1×10^-1^ |
| Cerebral gray matter volume | COPMaxSpeed_s | 1.5×10^-2^ | 1.8×10^-2^ | 4.2×10^-1^ |
| Cerebral gray matter volume | COMClockfaceCircularity_s | 1.5×10^-2^ | 1.8×10^-2^ | 4.2×10^-1^ |
| Cerebral gray matter volume | COMInkLength | 1.5×10^-2^ | 1.9×10^-2^ | 4.3×10^-1^ |
| Cerebral gray matter volume | COMPercentInkTime | -1.4×10^-2^ | 1.9×10^-2^ | 4.4×10^-1^ |
| Cerebral gray matter volume | COPInkLength | 1.4×10^-2^ | 1.9×10^-2^ | 4.4×10^-1^ |
| Cerebral gray matter volume | COMPercentThinkTime | 1.4×10^-2^ | 1.9×10^-2^ | 4.5×10^-1^ |
| Cerebral gray matter volume | COMPercentThinkTime_s | -1.4×10^-2^ | 1.9×10^-2^ | 4.5×10^-1^ |
| Cerebral gray matter volume | COMPercentInkTime_s | 1.3×10^-2^ | 1.9×10^-2^ | 4.7×10^-1^ |
| Cerebral gray matter volume | COPStrokeCountConformity_s | -1.4×10^-2^ | 1.9×10^-2^ | 4.7×10^-1^ |
| Cerebral gray matter volume | COPLongestLatency | -1.3×10^-2^ | 1.9×10^-2^ | 4.8×10^-1^ |
| Cerebral gray matter volume | COPClockfaceCircularity_s | 1.3×10^-2^ | 1.8×10^-2^ | 4.9×10^-1^ |
| Cerebral gray matter volume | COPSpatialReasoning | 1.2×10^-2^ | 1.9×10^-2^ | 5.1×10^-1^ |
| Cerebral gray matter volume | COMClockfaceCircularity | -9.4×10^-3^ | 1.9×10^-2^ | 6.2×10^-1^ |
| Cerebral gray matter volume | COPPercentInkTime | -8.8×10^-3^ | 1.9×10^-2^ | 6.4×10^-1^ |
| Cerebral gray matter volume | COPSpatialReasoning_s | 7.8×10^-3^ | 1.9×10^-2^ | 6.7×10^-1^ |
| Cerebral gray matter volume | COMMaxSpeed | 7.4×10^-3^ | 1.9×10^-2^ | 6.9×10^-1^ |
| Cerebral gray matter volume | COPLongLatencyCount | -6.8×10^-3^ | 2.0×10^-2^ | 7.3×10^-1^ |
| Cerebral gray matter volume | COPPercentThinkTime_s | -5.7×10^-3^ | 1.9×10^-2^ | 7.6×10^-1^ |
| Cerebral gray matter volume | COMMaxSpeed_s | 4.7×10^-3^ | 1.8×10^-2^ | 8.0×10^-1^ |
| Cerebral gray matter volume | COPClockfaceCircularity | 4.1×10^-3^ | 1.8×10^-2^ | 8.2×10^-1^ |
| Cerebral gray matter volume | COMDrawingSize_s | 1.2×10^-3^ | 1.9×10^-2^ | 9.5×10^-1^ |
| Cerebral gray matter volume | COMDrawingSize | -2.2×10^-4^ | 1.9×10^-2^ | 9.9×10^-1^ |
|  |  |  |  |  |
| Hippocampus volume | COPOscillatoryMotion | -6.9×10^-2^ | 2.5×10^-2^ | 5.5×10^-3^ |
| Hippocampus volume | DCTScore | 6.8×10^-2^ | 2.6×10^-2^ | 9.0×10^-3^ |
| Hippocampus volume | COPSpatialReasoning | 5.7×10^-2^ | 2.5×10^-2^ | 2.1×10^-2^ |
| Hippocampus volume | COPSimpleMotor | 5.5×10^-2^ | 2.5×10^-2^ | 2.6×10^-2^ |
| Hippocampus volume | COPAverageLatency | -5.3×10^-2^ | 2.5×10^-2^ | 3.6×10^-2^ |
| Hippocampus volume | COPTotalTime | -5.2×10^-2^ | 2.5×10^-2^ | 3.8×10^-2^ |
| Hippocampus volume | COPDrawingProcessEfficiency | 5.1×10^-2^ | 2.5×10^-2^ | 4.1×10^-2^ |
| Hippocampus volume | COPSpatialReasoning_s | 4.9×10^-2^ | 2.4×10^-2^ | 4.2×10^-2^ |
| Hippocampus volume | COPOscillatoryMotion_s | 4.6×10^-2^ | 2.4×10^-2^ | 5.9×10^-2^ |
| Hippocampus volume | COPComponentPlacement | -4.5×10^-2^ | 2.5×10^-2^ | 7.0×10^-2^ |
| Hippocampus volume | COMSimpleMotor | 4.5×10^-2^ | 2.5×10^-2^ | 7.1×10^-2^ |
| Hippocampus volume | COPStrokeCountConformity_s | -4.5×10^-2^ | 2.5×10^-2^ | 7.5×10^-2^ |
| Hippocampus volume | COPDrawingEfficiency | 4.3×10^-2^ | 2.5×10^-2^ | 8.7×10^-2^ |
| Hippocampus volume | COMTotalTime | -4.2×10^-2^ | 2.5×10^-2^ | 9.0×10^-2^ |
| Hippocampus volume | COPVerticalSpatialPlacement | -4.1×10^-2^ | 2.4×10^-2^ | 9.0×10^-2^ |
| Hippocampus volume | COPComponentPlacement_s | 4.1×10^-2^ | 2.4×10^-2^ | 9.1×10^-2^ |
| Hippocampus volume | COPSimpleMotor_s | 4.0×10^-2^ | 2.4×10^-2^ | 9.3×10^-2^ |
| Hippocampus volume | COMHorizontalSpatialPlacement | 4.0×10^-2^ | 2.4×10^-2^ | 9.9×10^-2^ |
| Hippocampus volume | COPLongestLatency | -4.1×10^-2^ | 2.5×10^-2^ | 1.0×10^-1^ |
| Hippocampus volume | COPInformationProcessing | 4.1×10^-2^ | 2.5×10^-2^ | 1.0×10^-1^ |
| Hippocampus volume | COMOscillatoryMotion | -4.2×10^-2^ | 2.6×10^-2^ | 1.0×10^-1^ |
| Hippocampus volume | COPDrawingProcessEfficiency_s | 4.0×10^-2^ | 2.5×10^-2^ | 1.1×10^-1^ |
| Hippocampus volume | COMHorizontalSpatialPlacement_s | -3.7×10^-2^ | 2.4×10^-2^ | 1.2×10^-1^ |
| Hippocampus volume | COPAverageLatency_s | 3.7×10^-2^ | 2.5×10^-2^ | 1.3×10^-1^ |
| Hippocampus volume | COMAverageSpeed | 3.6×10^-2^ | 2.4×10^-2^ | 1.4×10^-1^ |
| Hippocampus volume | COPDrawingEfficiency_s | 3.6×10^-2^ | 2.5×10^-2^ | 1.5×10^-1^ |
| Hippocampus volume | COMSimpleMotor_s | 3.5×10^-2^ | 2.5×10^-2^ | 1.5×10^-1^ |
| Hippocampus volume | COPTotalTime_s | 3.4×10^-2^ | 2.5×10^-2^ | 1.7×10^-1^ |
| Hippocampus volume | COMInitiationSpeed | 3.2×10^-2^ | 2.4×10^-2^ | 1.8×10^-1^ |
| Hippocampus volume | COMTotalTime_s | 3.2×10^-2^ | 2.4×10^-2^ | 1.9×10^-1^ |
| Hippocampus volume | COPLatencyVariability | -3.2×10^-2^ | 2.5×10^-2^ | 2.0×10^-1^ |
| Hippocampus volume | COMTerminationSpeed | 3.1×10^-2^ | 2.4×10^-2^ | 2.1×10^-1^ |
| Hippocampus volume | COPRelativeLongLatency | -3.2×10^-2^ | 2.5×10^-2^ | 2.1×10^-1^ |
| Hippocampus volume | COMAverageSpeed_s | 2.9×10^-2^ | 2.4×10^-2^ | 2.4×10^-1^ |
| Hippocampus volume | COPInformationProcessing_s | 2.9×10^-2^ | 2.5×10^-2^ | 2.5×10^-1^ |
| Hippocampus volume | COMMaxSpeed | 2.8×10^-2^ | 2.4×10^-2^ | 2.5×10^-1^ |
| Hippocampus volume | COMInitiationSpeed_s | 2.7×10^-2^ | 2.4×10^-2^ | 2.7×10^-1^ |
| Hippocampus volume | COPLongestLatency_s | 2.7×10^-2^ | 2.4×10^-2^ | 2.8×10^-1^ |
| Hippocampus volume | COMOscillatoryMotion_s | 2.7×10^-2^ | 2.5×10^-2^ | 2.8×10^-1^ |
| Hippocampus volume | COMInkLength | -2.5×10^-2^ | 2.5×10^-2^ | 3.0×10^-1^ |
| Hippocampus volume | COPLongLatencyCount | -2.6×10^-2^ | 2.6×10^-2^ | 3.2×10^-1^ |
| Hippocampus volume | COPVerticalSpatialPlacement_s | 2.4×10^-2^ | 2.4×10^-2^ | 3.2×10^-1^ |
| Hippocampus volume | COMInkLength_s | -2.5×10^-2^ | 2.5×10^-2^ | 3.2×10^-1^ |
| Hippocampus volume | COMAverageLatency | -2.5×10^-2^ | 2.5×10^-2^ | 3.2×10^-1^ |
| Hippocampus volume | COMTerminationSpeed_s | 2.4×10^-2^ | 2.4×10^-2^ | 3.3×10^-1^ |
| Hippocampus volume | COMClockfaceCircularity | -2.3×10^-2^ | 2.5×10^-2^ | 3.5×10^-1^ |
| Hippocampus volume | COPMaxSpeed | 2.1×10^-2^ | 2.4×10^-2^ | 3.7×10^-1^ |
| Hippocampus volume | COMMaxSpeed_s | 2.1×10^-2^ | 2.4×10^-2^ | 3.9×10^-1^ |
| Hippocampus volume | COPLongLatencyCount_s | 2.2×10^-2^ | 2.5×10^-2^ | 3.9×10^-1^ |
| Hippocampus volume | COMLongestLatency | -2.0×10^-2^ | 2.4×10^-2^ | 4.0×10^-1^ |
| Hippocampus volume | COMLongLatencyCount_s | 2.0×10^-2^ | 2.5×10^-2^ | 4.1×10^-1^ |
| Hippocampus volume | COPAverageSpeed | 2.0×10^-2^ | 2.4×10^-2^ | 4.1×10^-1^ |
| Hippocampus volume | COPTerminationSpeed | 1.9×10^-2^ | 2.4×10^-2^ | 4.2×10^-1^ |
| Hippocampus volume | COMInformationProcessing | 1.9×10^-2^ | 2.5×10^-2^ | 4.3×10^-1^ |
| Hippocampus volume | COPRelativeLongLatency_s | 1.9×10^-2^ | 2.5×10^-2^ | 4.6×10^-1^ |
| Hippocampus volume | COMLatencyVariability | -1.8×10^-2^ | 2.5×10^-2^ | 4.6×10^-1^ |
| Hippocampus volume | COMPercentThinkTime_s | -1.8×10^-2^ | 2.4×10^-2^ | 4.7×10^-1^ |
| Hippocampus volume | COMSpatialReasoning | 1.8×10^-2^ | 2.5×10^-2^ | 4.7×10^-1^ |
| Hippocampus volume | COMPercentInkTime_s | 1.7×10^-2^ | 2.4×10^-2^ | 4.8×10^-1^ |
| Hippocampus volume | COPLatencyVariability_s | 1.7×10^-2^ | 2.5×10^-2^ | 4.9×10^-1^ |
| Hippocampus volume | COMSpatialReasoning_s | 1.7×10^-2^ | 2.5×10^-2^ | 4.9×10^-1^ |
| Hippocampus volume | COMAverageLatency_s | 1.7×10^-2^ | 2.5×10^-2^ | 5.0×10^-1^ |
| Hippocampus volume | COMVerticalSpatialPlacement_s | 1.6×10^-2^ | 2.4×10^-2^ | 5.1×10^-1^ |
| Hippocampus volume | COPInitiationSpeed | 1.6×10^-2^ | 2.4×10^-2^ | 5.1×10^-1^ |
| Hippocampus volume | COMDrawingProcessEfficiency | 1.6×10^-2^ | 2.5×10^-2^ | 5.2×10^-1^ |
| Hippocampus volume | COMVerticalSpatialPlacement | -1.5×10^-2^ | 2.4×10^-2^ | 5.2×10^-1^ |
| Hippocampus volume | COMPercentInkTime | -1.5×10^-2^ | 2.4×10^-2^ | 5.3×10^-1^ |
| Hippocampus volume | COMPercentThinkTime | 1.5×10^-2^ | 2.4×10^-2^ | 5.4×10^-1^ |
| Hippocampus volume | COMRelativeLongLatency | -1.5×10^-2^ | 2.5×10^-2^ | 5.4×10^-1^ |
| Hippocampus volume | COMClockfaceCircularity_s | 1.4×10^-2^ | 2.4×10^-2^ | 5.6×10^-1^ |
| Hippocampus volume | COMDrawingEfficiency | 1.4×10^-2^ | 2.5×10^-2^ | 5.8×10^-1^ |
| Hippocampus volume | COMLongestLatency_s | 1.3×10^-2^ | 2.4×10^-2^ | 5.9×10^-1^ |
| Hippocampus volume | COPStrokeCountConformity | 1.4×10^-2^ | 2.6×10^-2^ | 5.9×10^-1^ |
| Hippocampus volume | COMInformationProcessing_s | 1.3×10^-2^ | 2.4×10^-2^ | 5.9×10^-1^ |
| Hippocampus volume | COMNoise_s | 1.2×10^-2^ | 2.4×10^-2^ | 6.2×10^-1^ |
| Hippocampus volume | COPPercentInkTime_s | -1.2×10^-2^ | 2.5×10^-2^ | 6.3×10^-1^ |
| Hippocampus volume | COPPercentThinkTime_s | 1.2×10^-2^ | 2.5×10^-2^ | 6.4×10^-1^ |
| Hippocampus volume | COPHorizontalSpatialPlacement_s | -1.1×10^-2^ | 2.4×10^-2^ | 6.5×10^-1^ |
| Hippocampus volume | COMLatencyVariability_s | 1.0×10^-2^ | 2.4×10^-2^ | 6.7×10^-1^ |
| Hippocampus volume | COPTerminationSpeed_s | 9.7×10^-3^ | 2.4×10^-2^ | 6.9×10^-1^ |
| Hippocampus volume | COPMaxSpeed_s | 9.5×10^-3^ | 2.4×10^-2^ | 6.9×10^-1^ |
| Hippocampus volume | COPPercentThinkTime | -9.8×10^-3^ | 2.5×10^-2^ | 6.9×10^-1^ |
| Hippocampus volume | COPPercentInkTime | 9.7×10^-3^ | 2.5×10^-2^ | 6.9×10^-1^ |
| Hippocampus volume | COMLongLatencyCount | -1.0×10^-2^ | 2.5×10^-2^ | 7.0×10^-1^ |
| Hippocampus volume | COPClockfaceCircularity | -8.1×10^-3^ | 2.4×10^-2^ | 7.4×10^-1^ |
| Hippocampus volume | COPAverageSpeed_s | 8.0×10^-3^ | 2.4×10^-2^ | 7.4×10^-1^ |
| Hippocampus volume | COMStrokeCountConformity | -8.2×10^-3^ | 2.5×10^-2^ | 7.4×10^-1^ |
| Hippocampus volume | COMRelativeLongLatency_s | 6.1×10^-3^ | 2.4×10^-2^ | 8.0×10^-1^ |
| Hippocampus volume | COMDrawingEfficiency_s | 5.1×10^-3^ | 2.5×10^-2^ | 8.4×10^-1^ |
| Hippocampus volume | COPInitiationSpeed_s | 4.4×10^-3^ | 2.4×10^-2^ | 8.5×10^-1^ |
| Hippocampus volume | COPHorizontalSpatialPlacement | -4.2×10^-3^ | 2.4×10^-2^ | 8.6×10^-1^ |
| Hippocampus volume | COMNoise | -4.3×10^-3^ | 2.5×10^-2^ | 8.6×10^-1^ |
| Hippocampus volume | COMDrawingProcessEfficiency_s | 3.7×10^-3^ | 2.5×10^-2^ | 8.8×10^-1^ |
| Hippocampus volume | COPNoise | -3.1×10^-3^ | 2.5×10^-2^ | 9.0×10^-1^ |
| Hippocampus volume | COPDrawingSize_s | 3.0×10^-3^ | 2.4×10^-2^ | 9.0×10^-1^ |
| Hippocampus volume | COMDrawingSize | -2.9×10^-3^ | 2.5×10^-2^ | 9.1×10^-1^ |
| Hippocampus volume | COMDrawingSize_s | -2.5×10^-3^ | 2.5×10^-2^ | 9.2×10^-1^ |
| Hippocampus volume | COMStrokeCountConformity_s | 2.4×10^-3^ | 2.5×10^-2^ | 9.2×10^-1^ |
| Hippocampus volume | COPInkLength_s | 1.7×10^-3^ | 2.4×10^-2^ | 9.4×10^-1^ |
| Hippocampus volume | COMComponentPlacement | -1.5×10^-3^ | 2.5×10^-2^ | 9.5×10^-1^ |
| Hippocampus volume | COPNoise_s | 1.5×10^-3^ | 2.5×10^-2^ | 9.5×10^-1^ |
| Hippocampus volume | COPInkLength | -1.0×10^-3^ | 2.4×10^-2^ | 9.7×10^-1^ |
| Hippocampus volume | COMComponentPlacement_s | -9.5×10^-4^ | 2.5×10^-2^ | 9.7×10^-1^ |
| Hippocampus volume | COPClockfaceCircularity_s | -5.6×10^-4^ | 2.4×10^-2^ | 9.8×10^-1^ |
| Hippocampus volume | COPDrawingSize | -3.0×10^-4^ | 2.4×10^-2^ | 9.9×10^-1^ |
|  |  |  |  |  |
| WMH volume | COMDrawingProcessEfficiency | -4.9×10^-2^ | 1.8×10^-2^ | 6.6×10^-3^ |
| WMH volume | COMDrawingProcessEfficiency_s | -4.5×10^-2^ | 1.8×10^-2^ | 1.2×10^-2^ |
| WMH volume | COMLongestLatency | 4.1×10^-2^ | 1.8×10^-2^ | 2.0×10^-2^ |
| WMH volume | COMInformationProcessing | -4.1×10^-2^ | 1.8×10^-2^ | 2.1×10^-2^ |
| WMH volume | COMLatencyVariability | 4.1×10^-2^ | 1.8×10^-2^ | 2.1×10^-2^ |
| WMH volume | COMAverageLatency | 4.0×10^-2^ | 1.8×10^-2^ | 2.6×10^-2^ |
| WMH volume | COMTotalTime | 4.0×10^-2^ | 1.8×10^-2^ | 2.7×10^-2^ |
| WMH volume | COMDrawingEfficiency | -4.0×10^-2^ | 1.8×10^-2^ | 2.8×10^-2^ |
| WMH volume | COMLongestLatency_s | -3.7×10^-2^ | 1.7×10^-2^ | 3.3×10^-2^ |
| WMH volume | COPDrawingEfficiency | -3.8×10^-2^ | 1.8×10^-2^ | 3.4×10^-2^ |
| WMH volume | COPDrawingSize_s | -3.6×10^-2^ | 1.7×10^-2^ | 3.7×10^-2^ |
| WMH volume | COPDrawingProcessEfficiency | -3.8×10^-2^ | 1.8×10^-2^ | 3.7×10^-2^ |
| WMH volume | COPHorizontalSpatialPlacement | -3.6×10^-2^ | 1.7×10^-2^ | 3.7×10^-2^ |
| WMH volume | COMLatencyVariability_s | -3.6×10^-2^ | 1.8×10^-2^ | 3.9×10^-2^ |
| WMH volume | DCTScore | -3.8×10^-2^ | 1.9×10^-2^ | 4.0×10^-2^ |
| WMH volume | COPPercentThinkTime | -3.7×10^-2^ | 1.8×10^-2^ | 4.1×10^-2^ |
| WMH volume | COPDrawingSize | -3.6×10^-2^ | 1.7×10^-2^ | 4.2×10^-2^ |
| WMH volume | COMInformationProcessing_s | -3.6×10^-2^ | 1.8×10^-2^ | 4.2×10^-2^ |
| WMH volume | COMDrawingEfficiency_s | -3.6×10^-2^ | 1.8×10^-2^ | 4.5×10^-2^ |
| WMH volume | COPOscillatoryMotion | 3.6×10^-2^ | 1.8×10^-2^ | 4.7×10^-2^ |
| WMH volume | COMTotalTime_s | -3.3×10^-2^ | 1.8×10^-2^ | 6.1×10^-2^ |
| WMH volume | COPSimpleMotor | -3.3×10^-2^ | 1.8×10^-2^ | 6.2×10^-2^ |
| WMH volume | COPOscillatoryMotion_s | -3.1×10^-2^ | 1.7×10^-2^ | 7.3×10^-2^ |
| WMH volume | COMAverageLatency_s | -3.2×10^-2^ | 1.8×10^-2^ | 7.3×10^-2^ |
| WMH volume | COMRelativeLongLatency | 3.1×10^-2^ | 1.8×10^-2^ | 7.6×10^-2^ |
| WMH volume | COPMaxSpeed | -3.0×10^-2^ | 1.7×10^-2^ | 8.5×10^-2^ |
| WMH volume | COPDrawingProcessEfficiency_s | -3.1×10^-2^ | 1.8×10^-2^ | 8.5×10^-2^ |
| WMH volume | COPDrawingEfficiency_s | -3.0×10^-2^ | 1.8×10^-2^ | 9.0×10^-2^ |
| WMH volume | COPHorizontalSpatialPlacement_s | 2.9×10^-2^ | 1.7×10^-2^ | 9.4×10^-2^ |
| WMH volume | COPMaxSpeed_s | -2.8×10^-2^ | 1.7×10^-2^ | 1.0×10^-1^ |
| WMH volume | COMLongLatencyCount | 2.9×10^-2^ | 1.8×10^-2^ | 1.1×10^-1^ |
| WMH volume | COMRelativeLongLatency_s | -2.8×10^-2^ | 1.7×10^-2^ | 1.1×10^-1^ |
| WMH volume | COPPercentInkTime_s | -2.8×10^-2^ | 1.8×10^-2^ | 1.2×10^-1^ |
| WMH volume | COPInkLength_s | -2.6×10^-2^ | 1.7×10^-2^ | 1.3×10^-1^ |
| WMH volume | COPPercentInkTime | 2.6×10^-2^ | 1.8×10^-2^ | 1.5×10^-1^ |
| WMH volume | COMMaxSpeed | -2.5×10^-2^ | 1.8×10^-2^ | 1.6×10^-1^ |
| WMH volume | COPInkLength | -2.4×10^-2^ | 1.8×10^-2^ | 1.7×10^-1^ |
| WMH volume | COMMaxSpeed_s | -2.4×10^-2^ | 1.7×10^-2^ | 1.7×10^-1^ |
| WMH volume | COPLongestLatency_s | -2.4×10^-2^ | 1.8×10^-2^ | 1.7×10^-1^ |
| WMH volume | COPSimpleMotor_s | -2.3×10^-2^ | 1.7×10^-2^ | 1.8×10^-1^ |
| WMH volume | COMInitiationSpeed | -2.1×10^-2^ | 1.7×10^-2^ | 2.2×10^-1^ |
| WMH volume | COPLatencyVariability_s | -2.1×10^-2^ | 1.8×10^-2^ | 2.3×10^-1^ |
| WMH volume | COMLongLatencyCount_s | -2.1×10^-2^ | 1.8×10^-2^ | 2.4×10^-1^ |
| WMH volume | COMInitiationSpeed_s | -2.0×10^-2^ | 1.7×10^-2^ | 2.5×10^-1^ |
| WMH volume | COPLongLatencyCount_s | -2.1×10^-2^ | 1.8×10^-2^ | 2.5×10^-1^ |
| WMH volume | COMAverageSpeed | -2.0×10^-2^ | 1.8×10^-2^ | 2.5×10^-1^ |
| WMH volume | COPTotalTime_s | -2.0×10^-2^ | 1.8×10^-2^ | 2.5×10^-1^ |
| WMH volume | COPTotalTime | 2.1×10^-2^ | 1.8×10^-2^ | 2.5×10^-1^ |
| WMH volume | COPComponentPlacement | 2.0×10^-2^ | 1.8×10^-2^ | 2.6×10^-1^ |
| WMH volume | COPInformationProcessing | -2.0×10^-2^ | 1.8×10^-2^ | 2.7×10^-1^ |
| WMH volume | COPSpatialReasoning | -1.9×10^-2^ | 1.8×10^-2^ | 2.7×10^-1^ |
| WMH volume | COPInformationProcessing_s | -1.8×10^-2^ | 1.8×10^-2^ | 3.0×10^-1^ |
| WMH volume | COPStrokeCountConformity_s | -1.8×10^-2^ | 1.8×10^-2^ | 3.1×10^-1^ |
| WMH volume | COPPercentThinkTime_s | 1.8×10^-2^ | 1.8×10^-2^ | 3.2×10^-1^ |
| WMH volume | COMAverageSpeed_s | -1.7×10^-2^ | 1.7×10^-2^ | 3.2×10^-1^ |
| WMH volume | COMSimpleMotor | -1.8×10^-2^ | 1.8×10^-2^ | 3.2×10^-1^ |
| WMH volume | COPNoise | 1.6×10^-2^ | 1.8×10^-2^ | 3.5×10^-1^ |
| WMH volume | COPTerminationSpeed | -1.6×10^-2^ | 1.7×10^-2^ | 3.5×10^-1^ |
| WMH volume | COPAverageSpeed | -1.6×10^-2^ | 1.7×10^-2^ | 3.6×10^-1^ |
| WMH volume | COPAverageLatency_s | -1.6×10^-2^ | 1.8×10^-2^ | 3.8×10^-1^ |
| WMH volume | COPLongestLatency | 1.5×10^-2^ | 1.8×10^-2^ | 3.9×10^-1^ |
| WMH volume | COMNoise_s | -1.5×10^-2^ | 1.7×10^-2^ | 3.9×10^-1^ |
| WMH volume | COPTerminationSpeed_s | -1.4×10^-2^ | 1.7×10^-2^ | 4.1×10^-1^ |
| WMH volume | COMOscillatoryMotion | 1.5×10^-2^ | 1.9×10^-2^ | 4.2×10^-1^ |
| WMH volume | COPSpatialReasoning_s | -1.4×10^-2^ | 1.8×10^-2^ | 4.3×10^-1^ |
| WMH volume | COPAverageSpeed_s | -1.4×10^-2^ | 1.7×10^-2^ | 4.3×10^-1^ |
| WMH volume | COPVerticalSpatialPlacement | -1.3×10^-2^ | 1.7×10^-2^ | 4.6×10^-1^ |
| WMH volume | COPClockfaceCircularity_s | -1.3×10^-2^ | 1.7×10^-2^ | 4.6×10^-1^ |
| WMH volume | COPInitiationSpeed | -1.2×10^-2^ | 1.7×10^-2^ | 4.7×10^-1^ |
| WMH volume | COPComponentPlacement_s | -1.2×10^-2^ | 1.7×10^-2^ | 4.8×10^-1^ |
| WMH volume | COMVerticalSpatialPlacement_s | 1.2×10^-2^ | 1.7×10^-2^ | 4.8×10^-1^ |
| WMH volume | COPInitiationSpeed_s | -1.1×10^-2^ | 1.7×10^-2^ | 5.1×10^-1^ |
| WMH volume | COPStrokeCountConformity | -1.2×10^-2^ | 1.9×10^-2^ | 5.2×10^-1^ |
| WMH volume | COPLatencyVariability | 1.0×10^-2^ | 1.8×10^-2^ | 5.7×10^-1^ |
| WMH volume | COPAverageLatency | 1.0×10^-2^ | 1.8×10^-2^ | 5.7×10^-1^ |
| WMH volume | COMSimpleMotor_s | -1.0×10^-2^ | 1.8×10^-2^ | 5.7×10^-1^ |
| WMH volume | COPRelativeLongLatency_s | -9.7×10^-3^ | 1.8×10^-2^ | 5.9×10^-1^ |
| WMH volume | COMInkLength_s | -9.7×10^-3^ | 1.8×10^-2^ | 5.9×10^-1^ |
| WMH volume | COMStrokeCountConformity | -9.8×10^-3^ | 1.8×10^-2^ | 5.9×10^-1^ |
| WMH volume | COMTerminationSpeed | -9.3×10^-3^ | 1.8×10^-2^ | 6.0×10^-1^ |
| WMH volume | COMVerticalSpatialPlacement | -8.9×10^-3^ | 1.7×10^-2^ | 6.1×10^-1^ |
| WMH volume | COMInkLength | -8.4×10^-3^ | 1.8×10^-2^ | 6.4×10^-1^ |
| WMH volume | COPLongLatencyCount | -8.5×10^-3^ | 1.9×10^-2^ | 6.5×10^-1^ |
| WMH volume | COMNoise | -7.2×10^-3^ | 1.8×10^-2^ | 6.8×10^-1^ |
| WMH volume | COMTerminationSpeed_s | -7.1×10^-3^ | 1.8×10^-2^ | 6.9×10^-1^ |
| WMH volume | COMPercentInkTime_s | 6.1×10^-3^ | 1.8×10^-2^ | 7.3×10^-1^ |
| WMH volume | COMPercentThinkTime_s | -6.0×10^-3^ | 1.8×10^-2^ | 7.3×10^-1^ |
| WMH volume | COMComponentPlacement | 6.1×10^-3^ | 1.8×10^-2^ | 7.3×10^-1^ |
| WMH volume | COMDrawingSize_s | -5.9×10^-3^ | 1.8×10^-2^ | 7.4×10^-1^ |
| WMH volume | COMClockfaceCircularity | 5.5×10^-3^ | 1.8×10^-2^ | 7.6×10^-1^ |
| WMH volume | COMSpatialReasoning_s | 5.0×10^-3^ | 1.8×10^-2^ | 7.8×10^-1^ |
| WMH volume | COMStrokeCountConformity_s | -4.7×10^-3^ | 1.8×10^-2^ | 7.9×10^-1^ |
| WMH volume | COPVerticalSpatialPlacement_s | 3.9×10^-3^ | 1.7×10^-2^ | 8.2×10^-1^ |
| WMH volume | COMPercentThinkTime | 3.7×10^-3^ | 1.8×10^-2^ | 8.3×10^-1^ |
| WMH volume | COMPercentInkTime | -3.6×10^-3^ | 1.8×10^-2^ | 8.4×10^-1^ |
| WMH volume | COMSpatialReasoning | -2.8×10^-3^ | 1.8×10^-2^ | 8.8×10^-1^ |
| WMH volume | COMHorizontalSpatialPlacement_s | 2.5×10^-3^ | 1.8×10^-2^ | 8.8×10^-1^ |
| WMH volume | COPRelativeLongLatency | 1.9×10^-3^ | 1.8×10^-2^ | 9.1×10^-1^ |
| WMH volume | COPClockfaceCircularity | 1.7×10^-3^ | 1.7×10^-2^ | 9.2×10^-1^ |
| WMH volume | COMComponentPlacement_s | 1.7×10^-3^ | 1.8×10^-2^ | 9.2×10^-1^ |
| WMH volume | COMHorizontalSpatialPlacement | 1.6×10^-3^ | 1.7×10^-2^ | 9.3×10^-1^ |
| WMH volume | COMDrawingSize | -1.6×10^-3^ | 1.8×10^-2^ | 9.3×10^-1^ |
| WMH volume | COMClockfaceCircularity_s | 1.5×10^-3^ | 1.8×10^-2^ | 9.3×10^-1^ |
| WMH volume | COPNoise_s | -9.4×10^-4^ | 1.8×10^-2^ | 9.6×10^-1^ |
| WMH volume | COMOscillatoryMotion_s | -4.9×10^-4^ | 1.8×10^-2^ | 9.8×10^-1^ |
|  |  |  |  |  |
| Large WMH volume | COPPercentThinkTime | -2.5×10^-1^ | 7.2×10^-2^ | 6.7×10^-4^ |
| Large WMH volume | COPPercentInkTime_s | -2.3×10^-1^ | 7.3×10^-2^ | 2.0×10^-3^ |
| Large WMH volume | COPPercentInkTime | 2.2×10^-1^ | 7.2×10^-2^ | 2.7×10^-3^ |
| Large WMH volume | COMDrawingProcess×10fficiency | -2.2×10^-1^ | 7.5×10^-2^ | 3.2×10^-3^ |
| Large WMH volume | COPDrawing×10fficiency | -2.2×10^-1^ | 7.5×10^-2^ | 3.8×10^-3^ |
| Large WMH volume | COMDrawingProcess×10fficiency_s | -2.1×10^-1^ | 7.3×10^-2^ | 4.6×10^-3^ |
| Large WMH volume | COPMaxSpeed | -1.9×10^-1^ | 7.1×10^-2^ | 6.2×10^-3^ |
| Large WMH volume | COPDrawingProcess×10fficiency | -2.0×10^-1^ | 7.5×10^-2^ | 6.8×10^-3^ |
| Large WMH volume | COPPercentThinkTime_s | 2.0×10^-1^ | 7.3×10^-2^ | 7.2×10^-3^ |
| Large WMH volume | COPMaxSpeed_s | -1.9×10^-1^ | 7.0×10^-2^ | 7.7×10^-3^ |
| Large WMH volume | COPDrawing×10fficiency_s | -1.9×10^-1^ | 7.3×10^-2^ | 9.6×10^-3^ |
| Large WMH volume | COMTotalTime | 1.8×10^-1^ | 7.4×10^-2^ | 1.3×10^-2^ |
| Large WMH volume | COPDrawingProcess×10fficiency_s | -1.8×10^-1^ | 7.3×10^-2^ | 1.3×10^-2^ |
| Large WMH volume | COMDrawing×10fficiency | -1.7×10^-1^ | 7.4×10^-2^ | 2.0×10^-2^ |
| Large WMH volume | COPSimpleMotor | -1.7×10^-1^ | 7.2×10^-2^ | 2.2×10^-2^ |
| Large WMH volume | COMSimpleMotor | -1.7×10^-1^ | 7.5×10^-2^ | 2.3×10^-2^ |
| Large WMH volume | COMTotalTime_s | -1.6×10^-1^ | 7.2×10^-2^ | 2.5×10^-2^ |
| Large WMH volume | COPTerminationSpeed | -1.6×10^-1^ | 7.1×10^-2^ | 2.7×10^-2^ |
| Large WMH volume | COMOscillatoryMotion | 1.7×10^-1^ | 7.6×10^-2^ | 2.8×10^-2^ |
| Large WMH volume | COMDrawing×10fficiency_s | -1.6×10^-1^ | 7.3×10^-2^ | 2.9×10^-2^ |
| Large WMH volume | COPAverageSpeed | -1.5×10^-1^ | 7.1×10^-2^ | 2.9×10^-2^ |
| Large WMH volume | COMAverageLatency | 1.6×10^-1^ | 7.4×10^-2^ | 3.3×10^-2^ |
| Large WMH volume | COPTerminationSpeed_s | -1.5×10^-1^ | 7.0×10^-2^ | 3.4×10^-2^ |
| Large WMH volume | COPDrawingSize_s | -1.5×10^-1^ | 7.1×10^-2^ | 3.4×10^-2^ |
| Large WMH volume | COPAverageSpeed_s | -1.5×10^-1^ | 7.0×10^-2^ | 3.6×10^-2^ |
| Large WMH volume | COPDrawingSize | -1.5×10^-1^ | 7.1×10^-2^ | 3.8×10^-2^ |
| Large WMH volume | COMAverageSpeed | -1.4×10^-1^ | 7.2×10^-2^ | 4.7×10^-2^ |
| Large WMH volume | COMSimpleMotor_s | -1.5×10^-1^ | 7.4×10^-2^ | 4.8×10^-2^ |
| Large WMH volume | COPInkLength_s | -1.4×10^-1^ | 7.2×10^-2^ | 5.1×10^-2^ |
| Large WMH volume | COPSimpleMotor_s | -1.4×10^-1^ | 7.0×10^-2^ | 5.3×10^-2^ |
| Large WMH volume | COPInkLength | -1.4×10^-1^ | 7.2×10^-2^ | 5.8×10^-2^ |
| Large WMH volume | COMInformationProcessing | -1.3×10^-1^ | 7.3×10^-2^ | 6.3×10^-2^ |
| Large WMH volume | COMAverageSpeed_s | -1.3×10^-1^ | 7.1×10^-2^ | 6.4×10^-2^ |
| Large WMH volume | COMInitiationSpeed | -1.3×10^-1^ | 7.1×10^-2^ | 6.8×10^-2^ |
| Large WMH volume | COMLatencyVariability | 1.3×10^-1^ | 7.2×10^-2^ | 6.8×10^-2^ |
| Large WMH volume | COMAverageLatency_s | -1.3×10^-1^ | 7.1×10^-2^ | 6.8×10^-2^ |
| Large WMH volume | COMInitiationSpeed_s | -1.3×10^-1^ | 7.1×10^-2^ | 7.4×10^-2^ |
| Large WMH volume | COPInitiationSpeed | -1.2×10^-1^ | 7.0×10^-2^ | 7.7×10^-2^ |
| Large WMH volume | COMMaxSpeed | -1.3×10^-1^ | 7.2×10^-2^ | 7.8×10^-2^ |
| Large WMH volume | COMMaxSpeed_s | -1.3×10^-1^ | 7.2×10^-2^ | 8.0×10^-2^ |
| Large WMH volume | DCTScore | -1.3×10^-1^ | 7.7×10^-2^ | 8.4×10^-2^ |
| Large WMH volume | COMLongestLatency | 1.2×10^-1^ | 7.2×10^-2^ | 8.5×10^-2^ |
| Large WMH volume | COPInitiationSpeed_s | -1.2×10^-1^ | 6.9×10^-2^ | 8.9×10^-2^ |
| Large WMH volume | COMInformationProcessing_s | -1.2×10^-1^ | 7.1×10^-2^ | 1.0×10^-1^ |
| Large WMH volume | COMLatencyVariability_s | -1.2×10^-1^ | 7.1×10^-2^ | 1.0×10^-1^ |
| Large WMH volume | COPOscillatoryMotion | 1.2×10^-1^ | 7.3×10^-2^ | 1.1×10^-1^ |
| Large WMH volume | COPTotalTime_s | -1.2×10^-1^ | 7.3×10^-2^ | 1.1×10^-1^ |
| Large WMH volume | COPTotalTime | 1.2×10^-1^ | 7.5×10^-2^ | 1.1×10^-1^ |
| Large WMH volume | COMLongestLatency_s | -1.1×10^-1^ | 7.1×10^-2^ | 1.1×10^-1^ |
| Large WMH volume | COMTerminationSpeed | -1.1×10^-1^ | 7.2×10^-2^ | 1.4×10^-1^ |
| Large WMH volume | COMRelativeLongLatency | 1.0×10^-1^ | 7.2×10^-2^ | 1.6×10^-1^ |
| Large WMH volume | COMOscillatoryMotion_s | -1.0×10^-1^ | 7.4×10^-2^ | 1.6×10^-1^ |
| Large WMH volume | COMTerminationSpeed_s | -9.9×10^-2^ | 7.2×10^-2^ | 1.7×10^-1^ |
| Large WMH volume | COMRelativeLongLatency_s | -9.0×10^-2^ | 7.1×10^-2^ | 2.0×10^-1^ |
| Large WMH volume | COPHorizontalSpatialPlacement_s | 8.3×10^-2^ | 7.0×10^-2^ | 2.3×10^-1^ |
| Large WMH volume | COMLongLatencyCount | 9.0×10^-2^ | 7.6×10^-2^ | 2.3×10^-1^ |
| Large WMH volume | COMPercentThinkTime | -8.2×10^-2^ | 7.1×10^-2^ | 2.5×10^-1^ |
| Large WMH volume | COMPercentInkTime | 8.2×10^-2^ | 7.1×10^-2^ | 2.5×10^-1^ |
| Large WMH volume | COPOscillatoryMotion_s | -8.1×10^-2^ | 7.1×10^-2^ | 2.5×10^-1^ |
| Large WMH volume | COMPercentInkTime_s | -7.6×10^-2^ | 7.1×10^-2^ | 2.8×10^-1^ |
| Large WMH volume | COMPercentThinkTime_s | 7.6×10^-2^ | 7.1×10^-2^ | 2.9×10^-1^ |
| Large WMH volume | COMVerticalSpatialPlacement_s | 7.3×10^-2^ | 7.1×10^-2^ | 3.0×10^-1^ |
| Large WMH volume | COPHorizontalSpatialPlacement | -7.1×10^-2^ | 7.0×10^-2^ | 3.1×10^-1^ |
| Large WMH volume | COPLongestLatency_s | -7.1×10^-2^ | 7.2×10^-2^ | 3.2×10^-1^ |
| Large WMH volume | COMDrawingSize_s | -6.9×10^-2^ | 7.2×10^-2^ | 3.4×10^-1^ |
| Large WMH volume | COPNoise | 6.7×10^-2^ | 7.4×10^-2^ | 3.7×10^-1^ |
| Large WMH volume | COMDrawingSize | -5.6×10^-2^ | 7.2×10^-2^ | 4.4×10^-1^ |
| Large WMH volume | COPInformationProcessing | -5.2×10^-2^ | 7.4×10^-2^ | 4.8×10^-1^ |
| Large WMH volume | COMVerticalSpatialPlacement | -4.9×10^-2^ | 7.1×10^-2^ | 4.9×10^-1^ |
| Large WMH volume | COMNoise_s | -4.8×10^-2^ | 7.3×10^-2^ | 5.1×10^-1^ |
| Large WMH volume | COPLongLatencyCount_s | -4.6×10^-2^ | 7.1×10^-2^ | 5.2×10^-1^ |
| Large WMH volume | COPLongestLatency | 4.5×10^-2^ | 7.3×10^-2^ | 5.3×10^-1^ |
| Large WMH volume | COPInformationProcessing_s | -4.5×10^-2^ | 7.3×10^-2^ | 5.4×10^-1^ |
| Large WMH volume | COPLatencyVariability_s | -4.1×10^-2^ | 7.3×10^-2^ | 5.7×10^-1^ |
| Large WMH volume | COMInkLength_s | -3.8×10^-2^ | 7.2×10^-2^ | 6.0×10^-1^ |
| Large WMH volume | COMInkLength | -3.6×10^-2^ | 7.2×10^-2^ | 6.1×10^-1^ |
| Large WMH volume | COMSpatialReasoning_s | 3.3×10^-2^ | 7.1×10^-2^ | 6.4×10^-1^ |
| Large WMH volume | COPStrokeCountConformity | 3.6×10^-2^ | 8.0×10^-2^ | 6.6×10^-1^ |
| Large WMH volume | COMNoise | 3.2×10^-2^ | 7.5×10^-2^ | 6.7×10^-1^ |
| Large WMH volume | COPAverageLatency_s | -2.9×10^-2^ | 7.3×10^-2^ | 6.9×10^-1^ |
| Large WMH volume | COPClockfaceCircularity | -2.8×10^-2^ | 7.1×10^-2^ | 6.9×10^-1^ |
| Large WMH volume | COMComponentPlacement_s | 2.6×10^-2^ | 7.1×10^-2^ | 7.2×10^-1^ |
| Large WMH volume | COPClockfaceCircularity_s | 2.6×10^-2^ | 7.2×10^-2^ | 7.2×10^-1^ |
| Large WMH volume | COMClockfaceCircularity | 2.5×10^-2^ | 7.2×10^-2^ | 7.3×10^-1^ |
| Large WMH volume | COPSpatialReasoning | -2.3×10^-2^ | 7.3×10^-2^ | 7.5×10^-1^ |
| Large WMH volume | COPStrokeCountConformity_s | -1.9×10^-2^ | 6.9×10^-2^ | 7.9×10^-1^ |
| Large WMH volume | COMLongLatencyCount_s | -1.8×10^-2^ | 7.0×10^-2^ | 7.9×10^-1^ |
| Large WMH volume | COPNoise_s | 1.7×10^-2^ | 6.9×10^-2^ | 8.0×10^-1^ |
| Large WMH volume | COPLongLatencyCount | 2.0×10^-2^ | 7.8×10^-2^ | 8.0×10^-1^ |
| Large WMH volume | COPRelativeLongLatency | -1.7×10^-2^ | 7.4×10^-2^ | 8.2×10^-1^ |
| Large WMH volume | COMHorizontalSpatialPlacement_s | 1.6×10^-2^ | 7.1×10^-2^ | 8.2×10^-1^ |
| Large WMH volume | COPLatencyVariability | 1.5×10^-2^ | 7.3×10^-2^ | 8.3×10^-1^ |
| Large WMH volume | COPComponentPlacement_s | 1.4×10^-2^ | 7.1×10^-2^ | 8.4×10^-1^ |
| Large WMH volume | COPAverageLatency | 1.4×10^-2^ | 7.4×10^-2^ | 8.5×10^-1^ |
| Large WMH volume | COMHorizontalSpatialPlacement | 1.2×10^-2^ | 7.1×10^-2^ | 8.7×10^-1^ |
| Large WMH volume | COPSpatialReasoning_s | -9.2×10^-3^ | 7.2×10^-2^ | 9.0×10^-1^ |
| Large WMH volume | COMStrokeCountConformity_s | 8.7×10^-3^ | 6.9×10^-2^ | 9.0×10^-1^ |
| Large WMH volume | COPVerticalSpatialPlacement | -8.4×10^-3^ | 7.1×10^-2^ | 9.1×10^-1^ |
| Large WMH volume | COMSpatialReasoning | 8.5×10^-3^ | 7.4×10^-2^ | 9.1×10^-1^ |
| Large WMH volume | COPVerticalSpatialPlacement_s | 7.4×10^-3^ | 7.1×10^-2^ | 9.2×10^-1^ |
| Large WMH volume | COMClockfaceCircularity_s | 5.1×10^-3^ | 7.2×10^-2^ | 9.4×10^-1^ |
| Large WMH volume | COPRelativeLongLatency_s | -4.8×10^-3^ | 7.3×10^-2^ | 9.5×10^-1^ |
| Large WMH volume | COPComponentPlacement | 4.6×10^-3^ | 7.3×10^-2^ | 9.5×10^-1^ |
| Large WMH volume | COMStrokeCountConformity | 3.3×10^-3^ | 7.7×10^-2^ | 9.7×10^-1^ |
| Large WMH volume | COMComponentPlacement | 2.9×10^-3^ | 7.4×10^-2^ | 9.7×10^-1^ |
|  |  |  |  |  |
| Cortical gray matter | COPOscillatoryMotion_s | 7.9×10^-2^ | 1.8×10^-2^ | **1.1×10^-5^** |
| Cortical gray matter | COPOscillatoryMotion | -7.4×10^-2^ | 1.9×10^-2^ | **7.5×10^-5^** |
| Cortical gray matter | COPSimpleMotor | 6.9×10^-2^ | 1.8×10^-2^ | **1.8×10^-4^** |
| Cortical gray matter | COPSimpleMotor_s | 6.0×10^-2^ | 1.8×10^-2^ | 9.2×10^-4^ |
| Cortical gray matter | COMOscillatoryMotion | -6.1×10^-2^ | 1.9×10^-2^ | 1.7×10^-3^ |
| Cortical gray matter | COMOscillatoryMotion_s | 5.8×10^-2^ | 1.9×10^-2^ | 2.0×10^-3^ |
| Cortical gray matter | COPNoise_s | -5.5×10^-2^ | 1.9×10^-2^ | 3.1×10^-3^ |
| Cortical gray matter | DCTScore | 5.6×10^-2^ | 1.9×10^-2^ | 3.9×10^-3^ |
| Cortical gray matter | COMDrawingProcessEfficiency | 5.1×10^-2^ | 1.9×10^-2^ | 7.1×10^-3^ |
| Cortical gray matter | COMSimpleMotor | 4.7×10^-2^ | 1.9×10^-2^ | 1.2×10^-2^ |
| Cortical gray matter | COMTotalTime | -4.3×10^-2^ | 1.9×10^-2^ | 2.0×10^-2^ |
| Cortical gray matter | COMDrawingProcessEfficiency_s | 4.2×10^-2^ | 1.8×10^-2^ | 2.2×10^-2^ |
| Cortical gray matter | COMNoise | -4.1×10^-2^ | 1.8×10^-2^ | 2.6×10^-2^ |
| Cortical gray matter | COMNoise_s | 3.8×10^-2^ | 1.8×10^-2^ | 3.6×10^-2^ |
| Cortical gray matter | COMSimpleMotor_s | 3.8×10^-2^ | 1.8×10^-2^ | 3.6×10^-2^ |
| Cortical gray matter | COMVerticalSpatialPlacement | 3.7×10^-2^ | 1.8×10^-2^ | 3.9×10^-2^ |
| Cortical gray matter | COMComponentPlacement | -3.8×10^-2^ | 1.9×10^-2^ | 4.1×10^-2^ |
| Cortical gray matter | COMInformationProcessing | 3.7×10^-2^ | 1.8×10^-2^ | 4.3×10^-2^ |
| Cortical gray matter | COMTotalTime_s | 3.6×10^-2^ | 1.8×10^-2^ | 4.6×10^-2^ |
| Cortical gray matter | COMComponentPlacement_s | 3.7×10^-2^ | 1.9×10^-2^ | 4.8×10^-2^ |
| Cortical gray matter | COPDrawingProcessEfficiency | 3.7×10^-2^ | 1.9×10^-2^ | 5.0×10^-2^ |
| Cortical gray matter | COMHorizontalSpatialPlacement | 3.5×10^-2^ | 1.8×10^-2^ | 5.4×10^-2^ |
| Cortical gray matter | COMAverageLatency | -3.5×10^-2^ | 1.9×10^-2^ | 5.9×10^-2^ |
| Cortical gray matter | COPHorizontalSpatialPlacement | 3.2×10^-2^ | 1.8×10^-2^ | 7.3×10^-2^ |
| Cortical gray matter | COMInitiationSpeed | 3.2×10^-2^ | 1.8×10^-2^ | 7.7×10^-2^ |
| Cortical gray matter | COMRelativeLongLatency | -3.2×10^-2^ | 1.8×10^-2^ | 7.9×10^-2^ |
| Cortical gray matter | COMInformationProcessing_s | 3.2×10^-2^ | 1.8×10^-2^ | 8.0×10^-2^ |
| Cortical gray matter | COMLatencyVariability | -3.2×10^-2^ | 1.8×10^-2^ | 8.5×10^-2^ |
| Cortical gray matter | COMInitiationSpeed_s | 3.0×10^-2^ | 1.8×10^-2^ | 9.7×10^-2^ |
| Cortical gray matter | COPInformationProcessing | 3.1×10^-2^ | 1.9×10^-2^ | 9.7×10^-2^ |
| Cortical gray matter | COMLongLatencyCount | -3.1×10^-2^ | 1.9×10^-2^ | 1.0×10^-1^ |
| Cortical gray matter | COPInitiationSpeed | 2.9×10^-2^ | 1.8×10^-2^ | 1.0×10^-1^ |
| Cortical gray matter | COPDrawingProcessEfficiency_s | 3.0×10^-2^ | 1.9×10^-2^ | 1.0×10^-1^ |
| Cortical gray matter | COMDrawingEfficiency | 3.0×10^-2^ | 1.9×10^-2^ | 1.1×10^-1^ |
| Cortical gray matter | COPDrawingEfficiency | 3.0×10^-2^ | 1.9×10^-2^ | 1.1×10^-1^ |
| Cortical gray matter | COMLongestLatency | -2.9×10^-2^ | 1.8×10^-2^ | 1.1×10^-1^ |
| Cortical gray matter | COMLongLatencyCount_s | 2.9×10^-2^ | 1.9×10^-2^ | 1.1×10^-1^ |
| Cortical gray matter | COMSpatialReasoning_s | 2.9×10^-2^ | 1.9×10^-2^ | 1.2×10^-1^ |
| Cortical gray matter | COMAverageLatency_s | 2.8×10^-2^ | 1.8×10^-2^ | 1.3×10^-1^ |
| Cortical gray matter | COMSpatialReasoning | 2.8×10^-2^ | 1.9×10^-2^ | 1.3×10^-1^ |
| Cortical gray matter | COPVerticalSpatialPlacement | 2.7×10^-2^ | 1.8×10^-2^ | 1.3×10^-1^ |
| Cortical gray matter | COMStrokeCountConformity | -2.8×10^-2^ | 1.9×10^-2^ | 1.3×10^-1^ |
| Cortical gray matter | COPComponentPlacement | -2.7×10^-2^ | 1.8×10^-2^ | 1.4×10^-1^ |
| Cortical gray matter | COMRelativeLongLatency_s | 2.7×10^-2^ | 1.8×10^-2^ | 1.4×10^-1^ |
| Cortical gray matter | COMAverageSpeed | 2.6×10^-2^ | 1.8×10^-2^ | 1.5×10^-1^ |
| Cortical gray matter | COPLongLatencyCount_s | 2.7×10^-2^ | 1.9×10^-2^ | 1.5×10^-1^ |
| Cortical gray matter | COMLatencyVariability_s | 2.6×10^-2^ | 1.8×10^-2^ | 1.5×10^-1^ |
| Cortical gray matter | COMVerticalSpatialPlacement_s | -2.6×10^-2^ | 1.8×10^-2^ | 1.5×10^-1^ |
| Cortical gray matter | COMDrawingEfficiency_s | 2.6×10^-2^ | 1.9×10^-2^ | 1.6×10^-1^ |
| Cortical gray matter | COPTotalTime_s | 2.6×10^-2^ | 1.8×10^-2^ | 1.6×10^-1^ |
| Cortical gray matter | COMHorizontalSpatialPlacement_s | -2.5×10^-2^ | 1.8×10^-2^ | 1.7×10^-1^ |
| Cortical gray matter | COPNoise | 2.5×10^-2^ | 1.8×10^-2^ | 1.7×10^-1^ |
| Cortical gray matter | COPInformationProcessing_s | 2.5×10^-2^ | 1.9×10^-2^ | 1.8×10^-1^ |
| Cortical gray matter | COPInitiationSpeed_s | 2.4×10^-2^ | 1.8×10^-2^ | 1.8×10^-1^ |
| Cortical gray matter | COPAverageLatency_s | 2.5×10^-2^ | 1.9×10^-2^ | 1.8×10^-1^ |
| Cortical gray matter | COMLongestLatency_s | 2.4×10^-2^ | 1.8×10^-2^ | 1.8×10^-1^ |
| Cortical gray matter | COPRelativeLongLatency_s | 2.4×10^-2^ | 1.9×10^-2^ | 1.9×10^-1^ |
| Cortical gray matter | COPDrawingEfficiency_s | 2.4×10^-2^ | 1.9×10^-2^ | 1.9×10^-1^ |
| Cortical gray matter | COPAverageSpeed | 2.3×10^-2^ | 1.8×10^-2^ | 2.0×10^-1^ |
| Cortical gray matter | COMAverageSpeed_s | 2.3×10^-2^ | 1.8×10^-2^ | 2.0×10^-1^ |
| Cortical gray matter | COPLatencyVariability_s | 2.3×10^-2^ | 1.8×10^-2^ | 2.1×10^-1^ |
| Cortical gray matter | COPComponentPlacement_s | 2.2×10^-2^ | 1.8×10^-2^ | 2.3×10^-1^ |
| Cortical gray matter | COPLongestLatency_s | 2.2×10^-2^ | 1.8×10^-2^ | 2.4×10^-1^ |
| Cortical gray matter | COPTotalTime | -2.2×10^-2^ | 1.9×10^-2^ | 2.4×10^-1^ |
| Cortical gray matter | COPPercentThinkTime | 2.2×10^-2^ | 1.9×10^-2^ | 2.4×10^-1^ |
| Cortical gray matter | COPHorizontalSpatialPlacement_s | -2.0×10^-2^ | 1.8×10^-2^ | 2.6×10^-1^ |
| Cortical gray matter | COPTerminationSpeed | 2.0×10^-2^ | 1.8×10^-2^ | 2.7×10^-1^ |
| Cortical gray matter | COMTerminationSpeed | 2.0×10^-2^ | 1.8×10^-2^ | 2.8×10^-1^ |
| Cortical gray matter | COPDrawingSize_s | 1.9×10^-2^ | 1.8×10^-2^ | 2.9×10^-1^ |
| Cortical gray matter | COPAverageSpeed_s | 1.8×10^-2^ | 1.8×10^-2^ | 3.2×10^-1^ |
| Cortical gray matter | COPAverageLatency | -1.8×10^-2^ | 1.9×10^-2^ | 3.3×10^-1^ |
| Cortical gray matter | COPPercentInkTime_s | 1.8×10^-2^ | 1.8×10^-2^ | 3.3×10^-1^ |
| Cortical gray matter | COPDrawingSize | 1.7×10^-2^ | 1.8×10^-2^ | 3.5×10^-1^ |
| Cortical gray matter | COMStrokeCountConformity_s | 1.7×10^-2^ | 1.8×10^-2^ | 3.5×10^-1^ |
| Cortical gray matter | COPLatencyVariability | -1.7×10^-2^ | 1.9×10^-2^ | 3.6×10^-1^ |
| Cortical gray matter | COPTerminationSpeed_s | 1.6×10^-2^ | 1.8×10^-2^ | 3.7×10^-1^ |
| Cortical gray matter | COPRelativeLongLatency | -1.7×10^-2^ | 1.9×10^-2^ | 3.8×10^-1^ |
| Cortical gray matter | COMInkLength_s | 1.6×10^-2^ | 1.8×10^-2^ | 3.8×10^-1^ |
| Cortical gray matter | COPMaxSpeed | 1.6×10^-2^ | 1.8×10^-2^ | 3.9×10^-1^ |
| Cortical gray matter | COMTerminationSpeed_s | 1.6×10^-2^ | 1.8×10^-2^ | 3.9×10^-1^ |
| Cortical gray matter | COPLongestLatency | -1.5×10^-2^ | 1.9×10^-2^ | 4.1×10^-1^ |
| Cortical gray matter | COPInkLength_s | 1.5×10^-2^ | 1.8×10^-2^ | 4.1×10^-1^ |
| Cortical gray matter | COPStrokeCountConformity | 1.6×10^-2^ | 2.0×10^-2^ | 4.3×10^-1^ |
| Cortical gray matter | COMInkLength | 1.5×10^-2^ | 1.8×10^-2^ | 4.3×10^-1^ |
| Cortical gray matter | COPSpatialReasoning | 1.4×10^-2^ | 1.9×10^-2^ | 4.3×10^-1^ |
| Cortical gray matter | COPVerticalSpatialPlacement_s | -1.4×10^-2^ | 1.8×10^-2^ | 4.4×10^-1^ |
| Cortical gray matter | COPInkLength | 1.3×10^-2^ | 1.8×10^-2^ | 4.7×10^-1^ |
| Cortical gray matter | COMPercentInkTime | -1.2×10^-2^ | 1.8×10^-2^ | 5.1×10^-1^ |
| Cortical gray matter | COMClockfaceCircularity_s | 1.2×10^-2^ | 1.8×10^-2^ | 5.2×10^-1^ |
| Cortical gray matter | COMPercentThinkTime | 1.1×10^-2^ | 1.8×10^-2^ | 5.3×10^-1^ |
| Cortical gray matter | COMPercentThinkTime_s | -1.1×10^-2^ | 1.8×10^-2^ | 5.4×10^-1^ |
| Cortical gray matter | COPClockfaceCircularity_s | 1.1×10^-2^ | 1.8×10^-2^ | 5.5×10^-1^ |
| Cortical gray matter | COPStrokeCountConformity_s | -1.1×10^-2^ | 1.9×10^-2^ | 5.5×10^-1^ |
| Cortical gray matter | COPMaxSpeed_s | 1.0×10^-2^ | 1.8×10^-2^ | 5.6×10^-1^ |
| Cortical gray matter | COMPercentInkTime_s | 1.1×10^-2^ | 1.8×10^-2^ | 5.6×10^-1^ |
| Cortical gray matter | COPSpatialReasoning_s | 9.4×10^-3^ | 1.8×10^-2^ | 6.0×10^-1^ |
| Cortical gray matter | COPPercentInkTime | -8.3×10^-3^ | 1.9×10^-2^ | 6.5×10^-1^ |
| Cortical gray matter | COPLongLatencyCount | -8.3×10^-3^ | 1.9×10^-2^ | 6.7×10^-1^ |
| Cortical gray matter | COMClockfaceCircularity | -7.1×10^-3^ | 1.8×10^-2^ | 7.0×10^-1^ |
| Cortical gray matter | COMMaxSpeed | 5.9×10^-3^ | 1.8×10^-2^ | 7.4×10^-1^ |
| Cortical gray matter | COPClockfaceCircularity | 5.2×10^-3^ | 1.8×10^-2^ | 7.7×10^-1^ |
| Cortical gray matter | COPPercentThinkTime_s | -4.4×10^-3^ | 1.8×10^-2^ | 8.1×10^-1^ |
| Cortical gray matter | COMDrawingSize_s | 3.7×10^-3^ | 1.8×10^-2^ | 8.4×10^-1^ |
| Cortical gray matter | COMMaxSpeed_s | 3.1×10^-3^ | 1.8×10^-2^ | 8.6×10^-1^ |
| Cortical gray matter | COMDrawingSize | 1.9×10^-3^ | 1.9×10^-2^ | 9.2×10^-1^ |
|  |  |  |  |  |
| Frontal cortical gray matter | COPOscillatoryMotion_s | 7.2×10^-2^ | 1.8×10^-2^ | **8.0×10^-5^** |
| Frontal cortical gray matter | COPSimpleMotor | 6.4×10^-2^ | 1.9×10^-2^ | 5.6×10^-4^ |
| Frontal cortical gray matter | COPOscillatoryMotion | -6.4×10^-2^ | 1.9×10^-2^ | 6.5×10^-4^ |
| Frontal cortical gray matter | COPSimpleMotor_s | 5.8×10^-2^ | 1.8×10^-2^ | 1.5×10^-3^ |
| Frontal cortical gray matter | COPVerticalSpatialPlacement | 5.0×10^-2^ | 1.8×10^-2^ | 6.2×10^-3^ |
| Frontal cortical gray matter | COMOscillatoryMotion | -5.1×10^-2^ | 1.9×10^-2^ | 9.4×10^-3^ |
| Frontal cortical gray matter | COMOscillatoryMotion_s | 4.9×10^-2^ | 1.9×10^-2^ | 9.6×10^-3^ |
| Frontal cortical gray matter | COPHorizontalSpatialPlacement | 4.5×10^-2^ | 1.8×10^-2^ | 1.2×10^-2^ |
| Frontal cortical gray matter | COPNoise_s | -4.5×10^-2^ | 1.9×10^-2^ | 1.6×10^-2^ |
| Frontal cortical gray matter | COPVerticalSpatialPlacement_s | -3.8×10^-2^ | 1.8×10^-2^ | 3.7×10^-2^ |
| Frontal cortical gray matter | COMSimpleMotor | 3.9×10^-2^ | 1.9×10^-2^ | 3.9×10^-2^ |
| Frontal cortical gray matter | COPStrokeCountConformity | 4.0×10^-2^ | 2.0×10^-2^ | 4.4×10^-2^ |
| Frontal cortical gray matter | COPNoise | 3.7×10^-2^ | 1.8×10^-2^ | 4.7×10^-2^ |
| Frontal cortical gray matter | COPHorizontalSpatialPlacement_s | -3.3×10^-2^ | 1.8×10^-2^ | 6.7×10^-2^ |
| Frontal cortical gray matter | COMSimpleMotor_s | 3.4×10^-2^ | 1.9×10^-2^ | 7.0×10^-2^ |
| Frontal cortical gray matter | COPPercentThinkTime | 3.1×10^-2^ | 1.9×10^-2^ | 9.6×10^-2^ |
| Frontal cortical gray matter | COMComponentPlacement | -3.1×10^-2^ | 1.9×10^-2^ | 1.0×10^-1^ |
| Frontal cortical gray matter | COMInitiationSpeed | 2.9×10^-2^ | 1.8×10^-2^ | 1.1×10^-1^ |
| Frontal cortical gray matter | COPInitiationSpeed | 2.8×10^-2^ | 1.8×10^-2^ | 1.1×10^-1^ |
| Frontal cortical gray matter | DCTScore | 3.1×10^-2^ | 2.0×10^-2^ | 1.1×10^-1^ |
| Frontal cortical gray matter | COPPercentInkTime_s | 2.9×10^-2^ | 1.9×10^-2^ | 1.1×10^-1^ |
| Frontal cortical gray matter | COMComponentPlacement_s | 2.9×10^-2^ | 1.9×10^-2^ | 1.2×10^-1^ |
| Frontal cortical gray matter | COMInitiationSpeed_s | 2.8×10^-2^ | 1.8×10^-2^ | 1.3×10^-1^ |
| Frontal cortical gray matter | COPAverageSpeed | 2.7×10^-2^ | 1.8×10^-2^ | 1.4×10^-1^ |
| Frontal cortical gray matter | COMVerticalSpatialPlacement | 2.6×10^-2^ | 1.8×10^-2^ | 1.6×10^-1^ |
| Frontal cortical gray matter | COPInitiationSpeed_s | 2.5×10^-2^ | 1.8×10^-2^ | 1.6×10^-1^ |
| Frontal cortical gray matter | COPTerminationSpeed | 2.5×10^-2^ | 1.8×10^-2^ | 1.6×10^-1^ |
| Frontal cortical gray matter | COMHorizontalSpatialPlacement | 2.5×10^-2^ | 1.8×10^-2^ | 1.8×10^-1^ |
| Frontal cortical gray matter | COPMaxSpeed | 2.4×10^-2^ | 1.8×10^-2^ | 1.8×10^-1^ |
| Frontal cortical gray matter | COPAverageSpeed_s | 2.3×10^-2^ | 1.8×10^-2^ | 2.0×10^-1^ |
| Frontal cortical gray matter | COPTerminationSpeed_s | 2.3×10^-2^ | 1.8×10^-2^ | 2.1×10^-1^ |
| Frontal cortical gray matter | COMAverageSpeed | 2.2×10^-2^ | 1.8×10^-2^ | 2.3×10^-1^ |
| Frontal cortical gray matter | COPMaxSpeed_s | 2.1×10^-2^ | 1.8×10^-2^ | 2.4×10^-1^ |
| Frontal cortical gray matter | COMSpatialReasoning_s | 2.2×10^-2^ | 1.9×10^-2^ | 2.5×10^-1^ |
| Frontal cortical gray matter | COPSpatialReasoning_s | -2.1×10^-2^ | 1.8×10^-2^ | 2.6×10^-1^ |
| Frontal cortical gray matter | COMSpatialReasoning | 2.1×10^-2^ | 1.9×10^-2^ | 2.6×10^-1^ |
| Frontal cortical gray matter | COMHorizontalSpatialPlacement_s | -2.0×10^-2^ | 1.8×10^-2^ | 2.7×10^-1^ |
| Frontal cortical gray matter | COMAverageSpeed_s | 2.0×10^-2^ | 1.8×10^-2^ | 2.8×10^-1^ |
| Frontal cortical gray matter | COMVerticalSpatialPlacement_s | -1.9×10^-2^ | 1.8×10^-2^ | 2.8×10^-1^ |
| Frontal cortical gray matter | COMLongLatencyCount_s | 2.0×10^-2^ | 1.9×10^-2^ | 2.9×10^-1^ |
| Frontal cortical gray matter | COMDrawingProcessEfficiency | 2.0×10^-2^ | 1.9×10^-2^ | 3.0×10^-1^ |
| Frontal cortical gray matter | COMTerminationSpeed | 1.9×10^-2^ | 1.8×10^-2^ | 3.1×10^-1^ |
| Frontal cortical gray matter | COPClockfaceCircularity | 1.8×10^-2^ | 1.8×10^-2^ | 3.2×10^-1^ |
| Frontal cortical gray matter | COPSpatialReasoning | -1.8×10^-2^ | 1.9×10^-2^ | 3.3×10^-1^ |
| Frontal cortical gray matter | COMInformationProcessing | 1.8×10^-2^ | 1.9×10^-2^ | 3.3×10^-1^ |
| Frontal cortical gray matter | COMStrokeCountConformity_s | 1.7×10^-2^ | 1.9×10^-2^ | 3.6×10^-1^ |
| Frontal cortical gray matter | COMStrokeCountConformity | -1.7×10^-2^ | 1.9×10^-2^ | 3.6×10^-1^ |
| Frontal cortical gray matter | COMLongLatencyCount | -1.7×10^-2^ | 1.9×10^-2^ | 3.9×10^-1^ |
| Frontal cortical gray matter | COMTerminationSpeed_s | 1.6×10^-2^ | 1.8×10^-2^ | 3.9×10^-1^ |
| Frontal cortical gray matter | COMDrawingProcessEfficiency_s | 1.6×10^-2^ | 1.9×10^-2^ | 3.9×10^-1^ |
| Frontal cortical gray matter | COMTotalTime | -1.6×10^-2^ | 1.9×10^-2^ | 4.0×10^-1^ |
| Frontal cortical gray matter | COPPercentInkTime | -1.5×10^-2^ | 1.9×10^-2^ | 4.1×10^-1^ |
| Frontal cortical gray matter | COMRelativeLongLatency | -1.5×10^-2^ | 1.9×10^-2^ | 4.2×10^-1^ |
| Frontal cortical gray matter | COMInformationProcessing_s | 1.5×10^-2^ | 1.8×10^-2^ | 4.2×10^-1^ |
| Frontal cortical gray matter | COMNoise_s | 1.4×10^-2^ | 1.8×10^-2^ | 4.4×10^-1^ |
| Frontal cortical gray matter | COPLongLatencyCount | 1.5×10^-2^ | 2.0×10^-2^ | 4.4×10^-1^ |
| Frontal cortical gray matter | COMLatencyVariability | -1.4×10^-2^ | 1.9×10^-2^ | 4.4×10^-1^ |
| Frontal cortical gray matter | COPStrokeCountConformity_s | -1.4×10^-2^ | 1.9×10^-2^ | 4.4×10^-1^ |
| Frontal cortical gray matter | COPPercentThinkTime_s | -1.4×10^-2^ | 1.9×10^-2^ | 4.6×10^-1^ |
| Frontal cortical gray matter | COPDrawingSize_s | 1.3×10^-2^ | 1.8×10^-2^ | 4.7×10^-1^ |
| Frontal cortical gray matter | COMMaxSpeed | 1.3×10^-2^ | 1.8×10^-2^ | 4.7×10^-1^ |
| Frontal cortical gray matter | COPDrawingProcessEfficiency | 1.4×10^-2^ | 1.9×10^-2^ | 4.7×10^-1^ |
| Frontal cortical gray matter | COPDrawingSize | 1.2×10^-2^ | 1.8×10^-2^ | 5.1×10^-1^ |
| Frontal cortical gray matter | COMRelativeLongLatency_s | 1.2×10^-2^ | 1.8×10^-2^ | 5.2×10^-1^ |
| Frontal cortical gray matter | COMTotalTime_s | 1.2×10^-2^ | 1.8×10^-2^ | 5.2×10^-1^ |
| Frontal cortical gray matter | COPDrawingEfficiency | 1.2×10^-2^ | 1.9×10^-2^ | 5.2×10^-1^ |
| Frontal cortical gray matter | COMDrawingEfficiency | 1.2×10^-2^ | 1.9×10^-2^ | 5.3×10^-1^ |
| Frontal cortical gray matter | COMAverageLatency | -1.2×10^-2^ | 1.9×10^-2^ | 5.3×10^-1^ |
| Frontal cortical gray matter | COMMaxSpeed_s | 1.1×10^-2^ | 1.8×10^-2^ | 5.3×10^-1^ |
| Frontal cortical gray matter | COMLatencyVariability_s | 1.1×10^-2^ | 1.8×10^-2^ | 5.4×10^-1^ |
| Frontal cortical gray matter | COPInkLength | 1.1×10^-2^ | 1.8×10^-2^ | 5.5×10^-1^ |
| Frontal cortical gray matter | COPInkLength_s | 1.1×10^-2^ | 1.8×10^-2^ | 5.5×10^-1^ |
| Frontal cortical gray matter | COMLongestLatency | -1.0×10^-2^ | 1.8×10^-2^ | 5.7×10^-1^ |
| Frontal cortical gray matter | COPLongestLatency | 9.7×10^-3^ | 1.9×10^-2^ | 6.0×10^-1^ |
| Frontal cortical gray matter | COPLongLatencyCount_s | 9.5×10^-3^ | 1.9×10^-2^ | 6.1×10^-1^ |
| Frontal cortical gray matter | COMDrawingEfficiency_s | 9.3×10^-3^ | 1.9×10^-2^ | 6.2×10^-1^ |
| Frontal cortical gray matter | COPDrawingProcessEfficiency_s | 9.0×10^-3^ | 1.9×10^-2^ | 6.3×10^-1^ |
| Frontal cortical gray matter | COMInkLength_s | 8.7×10^-3^ | 1.9×10^-2^ | 6.4×10^-1^ |
| Frontal cortical gray matter | COMPercentThinkTime_s | -8.2×10^-3^ | 1.8×10^-2^ | 6.6×10^-1^ |
| Frontal cortical gray matter | COPDrawingEfficiency_s | 8.3×10^-3^ | 1.9×10^-2^ | 6.6×10^-1^ |
| Frontal cortical gray matter | COMAverageLatency_s | 8.1×10^-3^ | 1.9×10^-2^ | 6.6×10^-1^ |
| Frontal cortical gray matter | COMPercentInkTime | -7.9×10^-3^ | 1.8×10^-2^ | 6.7×10^-1^ |
| Frontal cortical gray matter | COMPercentInkTime_s | 7.9×10^-3^ | 1.8×10^-2^ | 6.7×10^-1^ |
| Frontal cortical gray matter | COMLongestLatency_s | 7.7×10^-3^ | 1.8×10^-2^ | 6.7×10^-1^ |
| Frontal cortical gray matter | COMClockfaceCircularity_s | 7.8×10^-3^ | 1.8×10^-2^ | 6.7×10^-1^ |
| Frontal cortical gray matter | COMPercentThinkTime | 7.6×10^-3^ | 1.8×10^-2^ | 6.8×10^-1^ |
| Frontal cortical gray matter | COPInformationProcessing | 7.2×10^-3^ | 1.9×10^-2^ | 7.0×10^-1^ |
| Frontal cortical gray matter | COPTotalTime_s | 6.8×10^-3^ | 1.9×10^-2^ | 7.1×10^-1^ |
| Frontal cortical gray matter | COPComponentPlacement | -6.7×10^-3^ | 1.9×10^-2^ | 7.2×10^-1^ |
| Frontal cortical gray matter | COPLatencyVariability | 6.8×10^-3^ | 1.9×10^-2^ | 7.2×10^-1^ |
| Frontal cortical gray matter | COMInkLength | 6.7×10^-3^ | 1.9×10^-2^ | 7.2×10^-1^ |
| Frontal cortical gray matter | COPRelativeLongLatency_s | 6.3×10^-3^ | 1.9×10^-2^ | 7.3×10^-1^ |
| Frontal cortical gray matter | COMNoise | -6.3×10^-3^ | 1.9×10^-2^ | 7.3×10^-1^ |
| Frontal cortical gray matter | COPAverageLatency | 6.3×10^-3^ | 1.9×10^-2^ | 7.4×10^-1^ |
| Frontal cortical gray matter | COPClockfaceCircularity_s | -4.7×10^-3^ | 1.8×10^-2^ | 8.0×10^-1^ |
| Frontal cortical gray matter | COPRelativeLongLatency | 4.8×10^-3^ | 1.9×10^-2^ | 8.0×10^-1^ |
| Frontal cortical gray matter | COMClockfaceCircularity | -4.4×10^-3^ | 1.9×10^-2^ | 8.1×10^-1^ |
| Frontal cortical gray matter | COPAverageLatency_s | 4.2×10^-3^ | 1.9×10^-2^ | 8.2×10^-1^ |
| Frontal cortical gray matter | COPComponentPlacement_s | 3.7×10^-3^ | 1.8×10^-2^ | 8.4×10^-1^ |
| Frontal cortical gray matter | COMDrawingSize_s | 3.2×10^-3^ | 1.9×10^-2^ | 8.6×10^-1^ |
| Frontal cortical gray matter | COPLatencyVariability_s | 2.9×10^-3^ | 1.9×10^-2^ | 8.8×10^-1^ |
| Frontal cortical gray matter | COMDrawingSize | 2.5×10^-3^ | 1.9×10^-2^ | 8.9×10^-1^ |
| Frontal cortical gray matter | COPTotalTime | 2.2×10^-3^ | 1.9×10^-2^ | 9.1×10^-1^ |
| Frontal cortical gray matter | COPInformationProcessing_s | 2.2×10^-3^ | 1.9×10^-2^ | 9.1×10^-1^ |
| Frontal cortical gray matter | COPLongestLatency_s | 4.3×10^-4^ | 1.8×10^-2^ | 9.8×10^-1^ |
|  |  |  |  |  |
| Parietal cortical gray matter | COPSimpleMotor | 9.9×10^-2^ | 2.2×10^-2^ | **8.7×10^-6^** |
| Parietal cortical gray matter | COPDrawingEfficiency | 9.9×10^-2^ | 2.3×10^-2^ | **1.3×10^-5^** |
| Parietal cortical gray matter | COPDrawingProcessEfficiency | 9.6×10^-2^ | 2.3×10^-2^ | **2.5×10^-5^** |
| Parietal cortical gray matter | COPDrawingEfficiency_s | 9.3×10^-2^ | 2.2×10^-2^ | **3.6×10^-5^** |
| Parietal cortical gray matter | COPSimpleMotor_s | 8.9×10^-2^ | 2.2×10^-2^ | **4.3×10^-5^** |
| Parietal cortical gray matter | COPDrawingProcessEfficiency_s | 8.9×10^-2^ | 2.3×10^-2^ | **7.7×10^-5^** |
| Parietal cortical gray matter | COMInitiationSpeed | 8.3×10^-2^ | 2.2×10^-2^ | **1.6×10^-4^** |
| Parietal cortical gray matter | COMDrawingProcessEfficiency | 8.6×10^-2^ | 2.3×10^-2^ | **1.7×10^-4^** |
| Parietal cortical gray matter | COMAverageSpeed | 8.1×10^-2^ | 2.2×10^-2^ | **2.4×10^-4^** |
| Parietal cortical gray matter | COMInitiationSpeed_s | 8.0×10^-2^ | 2.2×10^-2^ | **2.6×10^-4^** |
| Parietal cortical gray matter | COMSimpleMotor | 8.1×10^-2^ | 2.3×10^-2^ | **3.7×10^-4^** |
| Parietal cortical gray matter | COMAverageSpeed_s | 7.7×10^-2^ | 2.2×10^-2^ | **4.6×10^-4^** |
| Parietal cortical gray matter | COPTerminationSpeed | 7.5×10^-2^ | 2.2×10^-2^ | 6.0×10^-4^ |
| Parietal cortical gray matter | COPAverageSpeed | 7.4×10^-2^ | 2.2×10^-2^ | 6.3×10^-4^ |
| Parietal cortical gray matter | COMDrawingProcessEfficiency_s | 7.4×10^-2^ | 2.2×10^-2^ | 9.9×10^-4^ |
| Parietal cortical gray matter | COPInitiationSpeed | 7.0×10^-2^ | 2.2×10^-2^ | 1.2×10^-3^ |
| Parietal cortical gray matter | COMSimpleMotor_s | 7.2×10^-2^ | 2.2×10^-2^ | 1.2×10^-3^ |
| Parietal cortical gray matter | COPTerminationSpeed_s | 7.0×10^-2^ | 2.2×10^-2^ | 1.3×10^-3^ |
| Parietal cortical gray matter | COPOscillatoryMotion_s | 6.9×10^-2^ | 2.2×10^-2^ | 1.5×10^-3^ |
| Parietal cortical gray matter | COPMaxSpeed | 6.9×10^-2^ | 2.2×10^-2^ | 1.6×10^-3^ |
| Parietal cortical gray matter | COMVerticalSpatialPlacement | 6.8×10^-2^ | 2.2×10^-2^ | 1.8×10^-3^ |
| Parietal cortical gray matter | COPAverageSpeed_s | 6.7×10^-2^ | 2.2×10^-2^ | 1.8×10^-3^ |
| Parietal cortical gray matter | DCTScore | 7.0×10^-2^ | 2.4×10^-2^ | 2.9×10^-3^ |
| Parietal cortical gray matter | COPInitiationSpeed_s | 6.3×10^-2^ | 2.1×10^-2^ | 3.5×10^-3^ |
| Parietal cortical gray matter | COMTerminationSpeed | 6.5×10^-2^ | 2.2×10^-2^ | 3.5×10^-3^ |
| Parietal cortical gray matter | COPMaxSpeed_s | 6.2×10^-2^ | 2.2×10^-2^ | 3.8×10^-3^ |
| Parietal cortical gray matter | COPTotalTime_s | 6.4×10^-2^ | 2.2×10^-2^ | 4.0×10^-3^ |
| Parietal cortical gray matter | COPTotalTime | -6.5×10^-2^ | 2.3×10^-2^ | 4.4×10^-3^ |
| Parietal cortical gray matter | COMDrawingEfficiency | 6.4×10^-2^ | 2.3×10^-2^ | 4.6×10^-3^ |
| Parietal cortical gray matter | COMTotalTime | -6.3×10^-2^ | 2.3×10^-2^ | 5.3×10^-3^ |
| Parietal cortical gray matter | COPInformationProcessing | 6.3×10^-2^ | 2.3×10^-2^ | 5.6×10^-3^ |
| Parietal cortical gray matter | COMTerminationSpeed_s | 6.0×10^-2^ | 2.2×10^-2^ | 6.9×10^-3^ |
| Parietal cortical gray matter | COPAverageLatency_s | 6.1×10^-2^ | 2.2×10^-2^ | 7.0×10^-3^ |
| Parietal cortical gray matter | COMDrawingEfficiency_s | 5.9×10^-2^ | 2.2×10^-2^ | 9.0×10^-3^ |
| Parietal cortical gray matter | COPOscillatoryMotion | -5.9×10^-2^ | 2.3×10^-2^ | 9.4×10^-3^ |
| Parietal cortical gray matter | COPAverageLatency | -5.9×10^-2^ | 2.3×10^-2^ | 1.0×10^-2^ |
| Parietal cortical gray matter | COMMaxSpeed | 5.7×10^-2^ | 2.2×10^-2^ | 1.0×10^-2^ |
| Parietal cortical gray matter | COMTotalTime_s | 5.5×10^-2^ | 2.2×10^-2^ | 1.3×10^-2^ |
| Parietal cortical gray matter | COPInformationProcessing_s | 5.6×10^-2^ | 2.2×10^-2^ | 1.3×10^-2^ |
| Parietal cortical gray matter | COPInkLength_s | 5.4×10^-2^ | 2.2×10^-2^ | 1.4×10^-2^ |
| Parietal cortical gray matter | COMNoise_s | 5.3×10^-2^ | 2.2×10^-2^ | 1.5×10^-2^ |
| Parietal cortical gray matter | COMOscillatoryMotion_s | 5.5×10^-2^ | 2.3×10^-2^ | 1.5×10^-2^ |
| Parietal cortical gray matter | COMVerticalSpatialPlacement_s | -5.3×10^-2^ | 2.2×10^-2^ | 1.5×10^-2^ |
| Parietal cortical gray matter | COMMaxSpeed_s | 5.3×10^-2^ | 2.2×10^-2^ | 1.7×10^-2^ |
| Parietal cortical gray matter | COPDrawingSize_s | 5.2×10^-2^ | 2.2×10^-2^ | 1.7×10^-2^ |
| Parietal cortical gray matter | COPInkLength | 5.2×10^-2^ | 2.2×10^-2^ | 1.9×10^-2^ |
| Parietal cortical gray matter | COMOscillatoryMotion | -5.4×10^-2^ | 2.3×10^-2^ | 2.3×10^-2^ |
| Parietal cortical gray matter | COPDrawingSize | 5.0×10^-2^ | 2.2×10^-2^ | 2.4×10^-2^ |
| Parietal cortical gray matter | COPRelativeLongLatency_s | 5.1×10^-2^ | 2.2×10^-2^ | 2.4×10^-2^ |
| Parietal cortical gray matter | COPLatencyVariability_s | 4.9×10^-2^ | 2.2×10^-2^ | 2.8×10^-2^ |
| Parietal cortical gray matter | COMNoise | -4.9×10^-2^ | 2.2×10^-2^ | 2.9×10^-2^ |
| Parietal cortical gray matter | COPLongestLatency_s | 4.8×10^-2^ | 2.2×10^-2^ | 3.2×10^-2^ |
| Parietal cortical gray matter | COPLatencyVariability | -4.7×10^-2^ | 2.3×10^-2^ | 3.6×10^-2^ |
| Parietal cortical gray matter | COPRelativeLongLatency | -4.7×10^-2^ | 2.3×10^-2^ | 3.8×10^-2^ |
| Parietal cortical gray matter | COPLongestLatency | -4.6×10^-2^ | 2.2×10^-2^ | 4.2×10^-2^ |
| Parietal cortical gray matter | COPLongLatencyCount_s | 4.6×10^-2^ | 2.3×10^-2^ | 4.4×10^-2^ |
| Parietal cortical gray matter | COMAverageLatency | -4.2×10^-2^ | 2.3×10^-2^ | 6.5×10^-2^ |
| Parietal cortical gray matter | COMInformationProcessing | 3.9×10^-2^ | 2.2×10^-2^ | 8.2×10^-2^ |
| Parietal cortical gray matter | COPNoise_s | -3.8×10^-2^ | 2.3×10^-2^ | 9.7×10^-2^ |
| Parietal cortical gray matter | COMInkLength_s | 3.7×10^-2^ | 2.2×10^-2^ | 1.0×10^-1^ |
| Parietal cortical gray matter | COMInkLength | 3.6×10^-2^ | 2.2×10^-2^ | 1.1×10^-1^ |
| Parietal cortical gray matter | COMRelativeLongLatency | -3.5×10^-2^ | 2.2×10^-2^ | 1.2×10^-1^ |
| Parietal cortical gray matter | COMAverageLatency_s | 3.4×10^-2^ | 2.2×10^-2^ | 1.3×10^-1^ |
| Parietal cortical gray matter | COMPercentInkTime | -3.3×10^-2^ | 2.2×10^-2^ | 1.4×10^-1^ |
| Parietal cortical gray matter | COMPercentThinkTime_s | -3.3×10^-2^ | 2.2×10^-2^ | 1.4×10^-1^ |
| Parietal cortical gray matter | COMInformationProcessing_s | 3.3×10^-2^ | 2.2×10^-2^ | 1.4×10^-1^ |
| Parietal cortical gray matter | COMPercentThinkTime | 3.2×10^-2^ | 2.2×10^-2^ | 1.4×10^-1^ |
| Parietal cortical gray matter | COMPercentInkTime_s | 3.2×10^-2^ | 2.2×10^-2^ | 1.4×10^-1^ |
| Parietal cortical gray matter | COMLongestLatency | -3.1×10^-2^ | 2.2×10^-2^ | 1.7×10^-1^ |
| Parietal cortical gray matter | COMLatencyVariability | -3.0×10^-2^ | 2.2×10^-2^ | 1.8×10^-1^ |
| Parietal cortical gray matter | COMLongLatencyCount_s | 2.9×10^-2^ | 2.3×10^-2^ | 1.9×10^-1^ |
| Parietal cortical gray matter | COPVerticalSpatialPlacement | 2.9×10^-2^ | 2.2×10^-2^ | 1.9×10^-1^ |
| Parietal cortical gray matter | COMRelativeLongLatency_s | 2.8×10^-2^ | 2.2×10^-2^ | 2.0×10^-1^ |
| Parietal cortical gray matter | COMStrokeCountConformity_s | 2.9×10^-2^ | 2.2×10^-2^ | 2.0×10^-1^ |
| Parietal cortical gray matter | COMLongestLatency_s | 2.5×10^-2^ | 2.2×10^-2^ | 2.6×10^-1^ |
| Parietal cortical gray matter | COPHorizontalSpatialPlacement_s | 2.4×10^-2^ | 2.2×10^-2^ | 2.7×10^-1^ |
| Parietal cortical gray matter | COMLatencyVariability_s | 2.3×10^-2^ | 2.2×10^-2^ | 2.9×10^-1^ |
| Parietal cortical gray matter | COPLongLatencyCount | -2.4×10^-2^ | 2.3×10^-2^ | 3.1×10^-1^ |
| Parietal cortical gray matter | COMDrawingSize_s | 2.0×10^-2^ | 2.2×10^-2^ | 3.7×10^-1^ |
| Parietal cortical gray matter | COMDrawingSize | 1.8×10^-2^ | 2.2×10^-2^ | 4.1×10^-1^ |
| Parietal cortical gray matter | COPClockfaceCircularity_s | 1.8×10^-2^ | 2.2×10^-2^ | 4.2×10^-1^ |
| Parietal cortical gray matter | COMComponentPlacement_s | 1.8×10^-2^ | 2.3×10^-2^ | 4.3×10^-1^ |
| Parietal cortical gray matter | COPComponentPlacement | -1.8×10^-2^ | 2.2×10^-2^ | 4.3×10^-1^ |
| Parietal cortical gray matter | COMLongLatencyCount | -1.8×10^-2^ | 2.3×10^-2^ | 4.3×10^-1^ |
| Parietal cortical gray matter | COMComponentPlacement | -1.6×10^-2^ | 2.3×10^-2^ | 4.8×10^-1^ |
| Parietal cortical gray matter | COPNoise | 1.6×10^-2^ | 2.2×10^-2^ | 4.8×10^-1^ |
| Parietal cortical gray matter | COPStrokeCountConformity | 1.6×10^-2^ | 2.4×10^-2^ | 5.2×10^-1^ |
| Parietal cortical gray matter | COMHorizontalSpatialPlacement_s | 1.3×10^-2^ | 2.2×10^-2^ | 5.4×10^-1^ |
| Parietal cortical gray matter | COPVerticalSpatialPlacement_s | -1.3×10^-2^ | 2.2×10^-2^ | 5.5×10^-1^ |
| Parietal cortical gray matter | COPComponentPlacement_s | 1.3×10^-2^ | 2.2×10^-2^ | 5.6×10^-1^ |
| Parietal cortical gray matter | COMClockfaceCircularity_s | 1.2×10^-2^ | 2.2×10^-2^ | 5.9×10^-1^ |
| Parietal cortical gray matter | COPHorizontalSpatialPlacement | -1.1×10^-2^ | 2.2×10^-2^ | 6.1×10^-1^ |
| Parietal cortical gray matter | COMSpatialReasoning_s | 1.1×10^-2^ | 2.3×10^-2^ | 6.4×10^-1^ |
| Parietal cortical gray matter | COMSpatialReasoning | 8.7×10^-3^ | 2.3×10^-2^ | 7.0×10^-1^ |
| Parietal cortical gray matter | COPSpatialReasoning | 8.5×10^-3^ | 2.2×10^-2^ | 7.1×10^-1^ |
| Parietal cortical gray matter | COMStrokeCountConformity | -7.9×10^-3^ | 2.3×10^-2^ | 7.3×10^-1^ |
| Parietal cortical gray matter | COPPercentThinkTime | 7.1×10^-3^ | 2.3×10^-2^ | 7.5×10^-1^ |
| Parietal cortical gray matter | COPStrokeCountConformity_s | 7.0×10^-3^ | 2.3×10^-2^ | 7.6×10^-1^ |
| Parietal cortical gray matter | COPPercentThinkTime_s | 5.6×10^-3^ | 2.2×10^-2^ | 8.0×10^-1^ |
| Parietal cortical gray matter | COPPercentInkTime_s | 4.7×10^-3^ | 2.2×10^-2^ | 8.4×10^-1^ |
| Parietal cortical gray matter | COPSpatialReasoning_s | 4.6×10^-3^ | 2.2×10^-2^ | 8.4×10^-1^ |
| Parietal cortical gray matter | COMClockfaceCircularity | -3.7×10^-3^ | 2.2×10^-2^ | 8.7×10^-1^ |
| Parietal cortical gray matter | COPPercentInkTime | 3.2×10^-3^ | 2.2×10^-2^ | 8.9×10^-1^ |
| Parietal cortical gray matter | COPClockfaceCircularity | 2.6×10^-3^ | 2.2×10^-2^ | 9.1×10^-1^ |
| Parietal cortical gray matter | COMHorizontalSpatialPlacement | 4.4×10^-4^ | 2.2×10^-2^ | 9.8×10^-1^ |
|  |  |  |  |  |
| Temporal cortical gray matter | COPOscillatoryMotion_s | 8.7×10^-2^ | 2.1×10^-2^ | **4.2×10^-5^** |
| Temporal cortical gray matter | COPOscillatoryMotion | -8.5×10^-2^ | 2.2×10^-2^ | **1.2×10^-4^** |
| Temporal cortical gray matter | DCTScore | 7.4×10^-2^ | 2.3×10^-2^ | 1.3×10^-3^ |
| Temporal cortical gray matter | COMOscillatoryMotion | -6.6×10^-2^ | 2.3×10^-2^ | 3.8×10^-3^ |
| Temporal cortical gray matter | COMOscillatoryMotion_s | 6.3×10^-2^ | 2.2×10^-2^ | 4.1×10^-3^ |
| Temporal cortical gray matter | COPComponentPlacement | -6.0×10^-2^ | 2.2×10^-2^ | 6.0×10^-3^ |
| Temporal cortical gray matter | COPSimpleMotor | 5.8×10^-2^ | 2.2×10^-2^ | 8.1×10^-3^ |
| Temporal cortical gray matter | COPComponentPlacement_s | 5.5×10^-2^ | 2.1×10^-2^ | 9.8×10^-3^ |
| Temporal cortical gray matter | COMDrawingProcessEfficiency | 5.7×10^-2^ | 2.2×10^-2^ | 1.1×10^-2^ |
| Temporal cortical gray matter | COMComponentPlacement | -5.6×10^-2^ | 2.2×10^-2^ | 1.1×10^-2^ |
| Temporal cortical gray matter | COMComponentPlacement_s | 5.5×10^-2^ | 2.2×10^-2^ | 1.2×10^-2^ |
| Temporal cortical gray matter | COMTotalTime | -5.4×10^-2^ | 2.2×10^-2^ | 1.4×10^-2^ |
| Temporal cortical gray matter | COMNoise | -5.3×10^-2^ | 2.2×10^-2^ | 1.6×10^-2^ |
| Temporal cortical gray matter | COPSpatialReasoning | 5.2×10^-2^ | 2.2×10^-2^ | 1.7×10^-2^ |
| Temporal cortical gray matter | COPSimpleMotor_s | 5.1×10^-2^ | 2.1×10^-2^ | 1.7×10^-2^ |
| Temporal cortical gray matter | COMTotalTime_s | 4.9×10^-2^ | 2.2×10^-2^ | 2.2×10^-2^ |
| Temporal cortical gray matter | COMDrawingProcessEfficiency_s | 4.9×10^-2^ | 2.2×10^-2^ | 2.4×10^-2^ |
| Temporal cortical gray matter | COPSpatialReasoning_s | 4.8×10^-2^ | 2.1×10^-2^ | 2.5×10^-2^ |
| Temporal cortical gray matter | COPNoise_s | -4.9×10^-2^ | 2.2×10^-2^ | 2.8×10^-2^ |
| Temporal cortical gray matter | COMSpatialReasoning_s | 4.8×10^-2^ | 2.2×10^-2^ | 2.9×10^-2^ |
| Temporal cortical gray matter | COMHorizontalSpatialPlacement | 4.5×10^-2^ | 2.1×10^-2^ | 3.3×10^-2^ |
| Temporal cortical gray matter | COMInformationProcessing | 4.6×10^-2^ | 2.2×10^-2^ | 3.6×10^-2^ |
| Temporal cortical gray matter | COMSpatialReasoning | 4.6×10^-2^ | 2.2×10^-2^ | 3.7×10^-2^ |
| Temporal cortical gray matter | COMAverageLatency | -4.5×10^-2^ | 2.2×10^-2^ | 4.1×10^-2^ |
| Temporal cortical gray matter | COMLatencyVariability | -4.4×10^-2^ | 2.2×10^-2^ | 4.3×10^-2^ |
| Temporal cortical gray matter | COMRelativeLongLatency | -4.3×10^-2^ | 2.2×10^-2^ | 4.8×10^-2^ |
| Temporal cortical gray matter | COMSimpleMotor | 4.3×10^-2^ | 2.2×10^-2^ | 5.2×10^-2^ |
| Temporal cortical gray matter | COMInformationProcessing_s | 4.2×10^-2^ | 2.2×10^-2^ | 5.2×10^-2^ |
| Temporal cortical gray matter | COMLatencyVariability_s | 4.0×10^-2^ | 2.1×10^-2^ | 6.1×10^-2^ |
| Temporal cortical gray matter | COMLongestLatency | -4.0×10^-2^ | 2.2×10^-2^ | 6.1×10^-2^ |
| Temporal cortical gray matter | COMStrokeCountConformity | -4.1×10^-2^ | 2.2×10^-2^ | 6.6×10^-2^ |
| Temporal cortical gray matter | COMAverageLatency_s | 4.0×10^-2^ | 2.2×10^-2^ | 7.0×10^-2^ |
| Temporal cortical gray matter | COMRelativeLongLatency_s | 3.9×10^-2^ | 2.1×10^-2^ | 7.2×10^-2^ |
| Temporal cortical gray matter | COMSimpleMotor_s | 3.7×10^-2^ | 2.2×10^-2^ | 8.9×10^-2^ |
| Temporal cortical gray matter | COMLongestLatency_s | 3.6×10^-2^ | 2.1×10^-2^ | 9.2×10^-2^ |
| Temporal cortical gray matter | COMLongLatencyCount | -3.8×10^-2^ | 2.2×10^-2^ | 9.2×10^-2^ |
| Temporal cortical gray matter | COMHorizontalSpatialPlacement_s | -3.5×10^-2^ | 2.1×10^-2^ | 1.0×10^-1^ |
| Temporal cortical gray matter | COMLongLatencyCount_s | 3.2×10^-2^ | 2.2×10^-2^ | 1.4×10^-1^ |
| Temporal cortical gray matter | COPLongLatencyCount | -3.3×10^-2^ | 2.3×10^-2^ | 1.5×10^-1^ |
| Temporal cortical gray matter | COMDrawingEfficiency | 3.2×10^-2^ | 2.2×10^-2^ | 1.5×10^-1^ |
| Temporal cortical gray matter | COPInformationProcessing | 3.2×10^-2^ | 2.2×10^-2^ | 1.5×10^-1^ |
| Temporal cortical gray matter | COPHorizontalSpatialPlacement | 3.0×10^-2^ | 2.1×10^-2^ | 1.6×10^-1^ |
| Temporal cortical gray matter | COPLongLatencyCount_s | 3.1×10^-2^ | 2.2×10^-2^ | 1.6×10^-1^ |
| Temporal cortical gray matter | COMNoise_s | 2.8×10^-2^ | 2.1×10^-2^ | 1.8×10^-1^ |
| Temporal cortical gray matter | COMDrawingEfficiency_s | 2.9×10^-2^ | 2.2×10^-2^ | 1.8×10^-1^ |
| Temporal cortical gray matter | COPInformationProcessing_s | 2.8×10^-2^ | 2.2×10^-2^ | 2.0×10^-1^ |
| Temporal cortical gray matter | COPLongestLatency_s | 2.7×10^-2^ | 2.2×10^-2^ | 2.0×10^-1^ |
| Temporal cortical gray matter | COPLatencyVariability_s | 2.7×10^-2^ | 2.2×10^-2^ | 2.2×10^-1^ |
| Temporal cortical gray matter | COMClockfaceCircularity_s | 2.6×10^-2^ | 2.1×10^-2^ | 2.2×10^-1^ |
| Temporal cortical gray matter | COPLongestLatency | -2.5×10^-2^ | 2.2×10^-2^ | 2.5×10^-1^ |
| Temporal cortical gray matter | COPRelativeLongLatency_s | 2.5×10^-2^ | 2.2×10^-2^ | 2.5×10^-1^ |
| Temporal cortical gray matter | COPLatencyVariability | -2.5×10^-2^ | 2.2×10^-2^ | 2.5×10^-1^ |
| Temporal cortical gray matter | COPDrawingProcessEfficiency | 2.3×10^-2^ | 2.2×10^-2^ | 2.9×10^-1^ |
| Temporal cortical gray matter | COMClockfaceCircularity | -2.2×10^-2^ | 2.2×10^-2^ | 3.1×10^-1^ |
| Temporal cortical gray matter | COPTotalTime | -2.2×10^-2^ | 2.2×10^-2^ | 3.2×10^-1^ |
| Temporal cortical gray matter | COPTotalTime_s | 2.1×10^-2^ | 2.2×10^-2^ | 3.3×10^-1^ |
| Temporal cortical gray matter | COPRelativeLongLatency | -2.2×10^-2^ | 2.2×10^-2^ | 3.3×10^-1^ |
| Temporal cortical gray matter | COMVerticalSpatialPlacement | 2.1×10^-2^ | 2.1×10^-2^ | 3.3×10^-1^ |
| Temporal cortical gray matter | COPHorizontalSpatialPlacement_s | -1.9×10^-2^ | 2.1×10^-2^ | 3.8×10^-1^ |
| Temporal cortical gray matter | COPPercentThinkTime | 1.9×10^-2^ | 2.2×10^-2^ | 3.8×10^-1^ |
| Temporal cortical gray matter | COPStrokeCountConformity | -2.0×10^-2^ | 2.3×10^-2^ | 3.8×10^-1^ |
| Temporal cortical gray matter | COPAverageLatency_s | 1.9×10^-2^ | 2.2×10^-2^ | 3.9×10^-1^ |
| Temporal cortical gray matter | COPDrawingProcessEfficiency_s | 1.9×10^-2^ | 2.2×10^-2^ | 4.0×10^-1^ |
| Temporal cortical gray matter | COMMaxSpeed_s | -1.6×10^-2^ | 2.1×10^-2^ | 4.4×10^-1^ |
| Temporal cortical gray matter | COPAverageLatency | -1.7×10^-2^ | 2.2×10^-2^ | 4.5×10^-1^ |
| Temporal cortical gray matter | COPPercentInkTime_s | 1.6×10^-2^ | 2.2×10^-2^ | 4.7×10^-1^ |
| Temporal cortical gray matter | COPStrokeCountConformity_s | -1.5×10^-2^ | 2.2×10^-2^ | 5.0×10^-1^ |
| Temporal cortical gray matter | COMMaxSpeed | -1.5×10^-2^ | 2.1×10^-2^ | 5.0×10^-1^ |
| Temporal cortical gray matter | COMInitiationSpeed | 1.4×10^-2^ | 2.1×10^-2^ | 5.2×10^-1^ |
| Temporal cortical gray matter | COMStrokeCountConformity_s | 1.4×10^-2^ | 2.2×10^-2^ | 5.2×10^-1^ |
| Temporal cortical gray matter | COPVerticalSpatialPlacement_s | 1.4×10^-2^ | 2.1×10^-2^ | 5.2×10^-1^ |
| Temporal cortical gray matter | COPDrawingSize_s | 1.3×10^-2^ | 2.1×10^-2^ | 5.4×10^-1^ |
| Temporal cortical gray matter | COPDrawingSize | 1.3×10^-2^ | 2.1×10^-2^ | 5.6×10^-1^ |
| Temporal cortical gray matter | COMInitiationSpeed_s | 1.2×10^-2^ | 2.1×10^-2^ | 5.6×10^-1^ |
| Temporal cortical gray matter | COMAverageSpeed | 1.2×10^-2^ | 2.1×10^-2^ | 5.7×10^-1^ |
| Temporal cortical gray matter | COPInitiationSpeed | 1.2×10^-2^ | 2.1×10^-2^ | 5.8×10^-1^ |
| Temporal cortical gray matter | COPPercentInkTime | -1.2×10^-2^ | 2.2×10^-2^ | 5.9×10^-1^ |
| Temporal cortical gray matter | COMTerminationSpeed | 1.1×10^-2^ | 2.2×10^-2^ | 6.1×10^-1^ |
| Temporal cortical gray matter | COMInkLength_s | 1.0×10^-2^ | 2.2×10^-2^ | 6.4×10^-1^ |
| Temporal cortical gray matter | COPClockfaceCircularity_s | 9.9×10^-3^ | 2.1×10^-2^ | 6.4×10^-1^ |
| Temporal cortical gray matter | COMVerticalSpatialPlacement_s | -9.6×10^-3^ | 2.1×10^-2^ | 6.5×10^-1^ |
| Temporal cortical gray matter | COMAverageSpeed_s | 9.1×10^-3^ | 2.1×10^-2^ | 6.7×10^-1^ |
| Temporal cortical gray matter | COMInkLength | 9.0×10^-3^ | 2.2×10^-2^ | 6.8×10^-1^ |
| Temporal cortical gray matter | COPPercentThinkTime_s | -8.5×10^-3^ | 2.2×10^-2^ | 7.0×10^-1^ |
| Temporal cortical gray matter | COMTerminationSpeed_s | 8.2×10^-3^ | 2.1×10^-2^ | 7.0×10^-1^ |
| Temporal cortical gray matter | COPInitiationSpeed_s | 7.7×10^-3^ | 2.1×10^-2^ | 7.1×10^-1^ |
| Temporal cortical gray matter | COPInkLength_s | -7.2×10^-3^ | 2.1×10^-2^ | 7.4×10^-1^ |
| Temporal cortical gray matter | COPInkLength | -7.1×10^-3^ | 2.2×10^-2^ | 7.4×10^-1^ |
| Temporal cortical gray matter | COPMaxSpeed_s | -6.7×10^-3^ | 2.1×10^-2^ | 7.5×10^-1^ |
| Temporal cortical gray matter | COPTerminationSpeed_s | -6.2×10^-3^ | 2.1×10^-2^ | 7.7×10^-1^ |
| Temporal cortical gray matter | COPDrawingEfficiency | 6.4×10^-3^ | 2.2×10^-2^ | 7.7×10^-1^ |
| Temporal cortical gray matter | COPNoise | 5.0×10^-3^ | 2.2×10^-2^ | 8.2×10^-1^ |
| Temporal cortical gray matter | COPVerticalSpatialPlacement | -3.6×10^-3^ | 2.1×10^-2^ | 8.7×10^-1^ |
| Temporal cortical gray matter | COPTerminationSpeed | -3.4×10^-3^ | 2.1×10^-2^ | 8.7×10^-1^ |
| Temporal cortical gray matter | COMPercentThinkTime_s | -3.3×10^-3^ | 2.2×10^-2^ | 8.8×10^-1^ |
| Temporal cortical gray matter | COMPercentInkTime | -3.3×10^-3^ | 2.2×10^-2^ | 8.8×10^-1^ |
| Temporal cortical gray matter | COPAverageSpeed_s | -3.0×10^-3^ | 2.1×10^-2^ | 8.9×10^-1^ |
| Temporal cortical gray matter | COPClockfaceCircularity | 3.0×10^-3^ | 2.1×10^-2^ | 8.9×10^-1^ |
| Temporal cortical gray matter | COMPercentThinkTime | 2.9×10^-3^ | 2.2×10^-2^ | 8.9×10^-1^ |
| Temporal cortical gray matter | COMPercentInkTime_s | 2.7×10^-3^ | 2.2×10^-2^ | 9.0×10^-1^ |
| Temporal cortical gray matter | COPMaxSpeed | -2.6×10^-3^ | 2.1×10^-2^ | 9.0×10^-1^ |
| Temporal cortical gray matter | COPDrawingEfficiency_s | 1.7×10^-3^ | 2.2×10^-2^ | 9.4×10^-1^ |
| Temporal cortical gray matter | COMDrawingSize | -1.3×10^-3^ | 2.2×10^-2^ | 9.5×10^-1^ |
| Temporal cortical gray matter | COPAverageSpeed | 5.4×10^-4^ | 2.1×10^-2^ | 9.8×10^-1^ |
| Temporal cortical gray matter | COMDrawingSize_s | -5.3×10^-5^ | 2.2×10^-2^ | 1.0×10^0^ |
|  |  |  |  |  |
| Occipital cortical gray matter | COPMaxSpeed_s | -6.0×10^-2^ | 2.2×10^-2^ | 7.3×10^-3^ |
| Occipital cortical gray matter | COPMaxSpeed | -5.6×10^-2^ | 2.3×10^-2^ | 1.2×10^-2^ |
| Occipital cortical gray matter | COMMaxSpeed_s | -4.8×10^-2^ | 2.3×10^-2^ | 3.5×10^-2^ |
| Occipital cortical gray matter | COPTerminationSpeed_s | -4.7×10^-2^ | 2.3×10^-2^ | 3.7×10^-2^ |
| Occipital cortical gray matter | COMMaxSpeed | -4.7×10^-2^ | 2.3×10^-2^ | 4.1×10^-2^ |
| Occipital cortical gray matter | COPTerminationSpeed | -4.5×10^-2^ | 2.3×10^-2^ | 4.9×10^-2^ |
| Occipital cortical gray matter | COMNoise | -4.5×10^-2^ | 2.3×10^-2^ | 5.2×10^-2^ |
| Occipital cortical gray matter | COPAverageSpeed_s | -4.2×10^-2^ | 2.3×10^-2^ | 6.5×10^-2^ |
| Occipital cortical gray matter | COMTerminationSpeed_s | -4.2×10^-2^ | 2.3×10^-2^ | 6.5×10^-2^ |
| Occipital cortical gray matter | COMHorizontalSpatialPlacement | 4.1×10^-2^ | 2.3×10^-2^ | 7.0×10^-2^ |
| Occipital cortical gray matter | COMNoise_s | 4.0×10^-2^ | 2.2×10^-2^ | 7.3×10^-2^ |
| Occipital cortical gray matter | COPNoise_s | -4.2×10^-2^ | 2.4×10^-2^ | 7.3×10^-2^ |
| Occipital cortical gray matter | COMAverageSpeed_s | -3.9×10^-2^ | 2.3×10^-2^ | 9.0×10^-2^ |
| Occipital cortical gray matter | COMTerminationSpeed | -3.9×10^-2^ | 2.3×10^-2^ | 9.1×10^-2^ |
| Occipital cortical gray matter | COPAverageSpeed | -3.8×10^-2^ | 2.3×10^-2^ | 9.6×10^-2^ |
| Occipital cortical gray matter | COMHorizontalSpatialPlacement_s | -3.7×10^-2^ | 2.3×10^-2^ | 1.0×10^-1^ |
| Occipital cortical gray matter | COMAverageSpeed | -3.7×10^-2^ | 2.3×10^-2^ | 1.1×10^-1^ |
| Occipital cortical gray matter | COMInitiationSpeed_s | -3.2×10^-2^ | 2.3×10^-2^ | 1.6×10^-1^ |
| Occipital cortical gray matter | COMLongLatencyCount | -3.3×10^-2^ | 2.4×10^-2^ | 1.7×10^-1^ |
| Occipital cortical gray matter | COMInitiationSpeed | -3.1×10^-2^ | 2.3×10^-2^ | 1.7×10^-1^ |
| Occipital cortical gray matter | COMSimpleMotor_s | -3.2×10^-2^ | 2.3×10^-2^ | 1.7×10^-1^ |
| Occipital cortical gray matter | COPInitiationSpeed_s | -2.9×10^-2^ | 2.2×10^-2^ | 1.9×10^-1^ |
| Occipital cortical gray matter | COPSimpleMotor_s | -2.6×10^-2^ | 2.3×10^-2^ | 2.5×10^-1^ |
| Occipital cortical gray matter | COPInitiationSpeed | -2.5×10^-2^ | 2.2×10^-2^ | 2.6×10^-1^ |
| Occipital cortical gray matter | COMStrokeCountConformity | -2.6×10^-2^ | 2.4×10^-2^ | 2.7×10^-1^ |
| Occipital cortical gray matter | COPClockfaceCircularity_s | 2.5×10^-2^ | 2.3×10^-2^ | 2.7×10^-1^ |
| Occipital cortical gray matter | COMAverageLatency | -2.4×10^-2^ | 2.4×10^-2^ | 3.0×10^-1^ |
| Occipital cortical gray matter | COPSpatialReasoning | 2.3×10^-2^ | 2.3×10^-2^ | 3.2×10^-1^ |
| Occipital cortical gray matter | COMSimpleMotor | -2.4×10^-2^ | 2.4×10^-2^ | 3.2×10^-1^ |
| Occipital cortical gray matter | COMInformationProcessing | 2.3×10^-2^ | 2.3×10^-2^ | 3.2×10^-1^ |
| Occipital cortical gray matter | COPDrawingSize | -2.3×10^-2^ | 2.3×10^-2^ | 3.2×10^-1^ |
| Occipital cortical gray matter | COPClockfaceCircularity | -2.3×10^-2^ | 2.3×10^-2^ | 3.2×10^-1^ |
| Occipital cortical gray matter | COPHorizontalSpatialPlacement_s | -2.2×10^-2^ | 2.3×10^-2^ | 3.2×10^-1^ |
| Occipital cortical gray matter | COPDrawingSize_s | -2.0×10^-2^ | 2.3×10^-2^ | 3.8×10^-1^ |
| Occipital cortical gray matter | COPHorizontalSpatialPlacement | 1.9×10^-2^ | 2.3×10^-2^ | 4.0×10^-1^ |
| Occipital cortical gray matter | COMLongestLatency | -1.9×10^-2^ | 2.3×10^-2^ | 4.2×10^-1^ |
| Occipital cortical gray matter | COMInformationProcessing_s | 1.8×10^-2^ | 2.3×10^-2^ | 4.2×10^-1^ |
| Occipital cortical gray matter | COPSimpleMotor | -1.8×10^-2^ | 2.3×10^-2^ | 4.3×10^-1^ |
| Occipital cortical gray matter | COMLatencyVariability | -1.8×10^-2^ | 2.3×10^-2^ | 4.3×10^-1^ |
| Occipital cortical gray matter | COMAverageLatency_s | 1.8×10^-2^ | 2.3×10^-2^ | 4.5×10^-1^ |
| Occipital cortical gray matter | COPDrawingEfficiency_s | -1.7×10^-2^ | 2.3×10^-2^ | 4.6×10^-1^ |
| Occipital cortical gray matter | COPSpatialReasoning_s | 1.6×10^-2^ | 2.3×10^-2^ | 4.8×10^-1^ |
| Occipital cortical gray matter | COMDrawingSize | -1.6×10^-2^ | 2.3×10^-2^ | 4.9×10^-1^ |
| Occipital cortical gray matter | COPVerticalSpatialPlacement_s | 1.5×10^-2^ | 2.3×10^-2^ | 5.0×10^-1^ |
| Occipital cortical gray matter | COMTotalTime | -1.6×10^-2^ | 2.4×10^-2^ | 5.0×10^-1^ |
| Occipital cortical gray matter | COMRelativeLongLatency | -1.5×10^-2^ | 2.3×10^-2^ | 5.2×10^-1^ |
| Occipital cortical gray matter | COPPercentThinkTime_s | 1.5×10^-2^ | 2.3×10^-2^ | 5.2×10^-1^ |
| Occipital cortical gray matter | COMLongestLatency_s | 1.4×10^-2^ | 2.3×10^-2^ | 5.3×10^-1^ |
| Occipital cortical gray matter | COPInkLength | -1.4×10^-2^ | 2.3×10^-2^ | 5.4×10^-1^ |
| Occipital cortical gray matter | COMClockfaceCircularity_s | -1.4×10^-2^ | 2.3×10^-2^ | 5.5×10^-1^ |
| Occipital cortical gray matter | COMDrawingSize_s | -1.3×10^-2^ | 2.3×10^-2^ | 5.7×10^-1^ |
| Occipital cortical gray matter | COMOscillatoryMotion | -1.4×10^-2^ | 2.4×10^-2^ | 5.7×10^-1^ |
| Occipital cortical gray matter | COMLatencyVariability_s | 1.3×10^-2^ | 2.3×10^-2^ | 5.8×10^-1^ |
| Occipital cortical gray matter | COPVerticalSpatialPlacement | -1.2×10^-2^ | 2.3×10^-2^ | 6.0×10^-1^ |
| Occipital cortical gray matter | COMLongLatencyCount_s | 1.2×10^-2^ | 2.4×10^-2^ | 6.0×10^-1^ |
| Occipital cortical gray matter | COMClockfaceCircularity | 1.2×10^-2^ | 2.3×10^-2^ | 6.0×10^-1^ |
| Occipital cortical gray matter | COPOscillatoryMotion | -1.2×10^-2^ | 2.4×10^-2^ | 6.1×10^-1^ |
| Occipital cortical gray matter | COMComponentPlacement | -1.2×10^-2^ | 2.4×10^-2^ | 6.1×10^-1^ |
| Occipital cortical gray matter | COMStrokeCountConformity_s | -1.1×10^-2^ | 2.3×10^-2^ | 6.2×10^-1^ |
| Occipital cortical gray matter | COMDrawingProcessEfficiency | 1.2×10^-2^ | 2.4×10^-2^ | 6.2×10^-1^ |
| Occipital cortical gray matter | COPDrawingEfficiency | -1.2×10^-2^ | 2.4×10^-2^ | 6.3×10^-1^ |
| Occipital cortical gray matter | COMRelativeLongLatency_s | 1.1×10^-2^ | 2.3×10^-2^ | 6.4×10^-1^ |
| Occipital cortical gray matter | COPNoise | 1.0×10^-2^ | 2.3×10^-2^ | 6.6×10^-1^ |
| Occipital cortical gray matter | COPDrawingProcessEfficiency_s | -1.0×10^-2^ | 2.4×10^-2^ | 6.7×10^-1^ |
| Occipital cortical gray matter | COPInformationProcessing | 9.9×10^-3^ | 2.4×10^-2^ | 6.8×10^-1^ |
| Occipital cortical gray matter | COMDrawingEfficiency_s | -9.7×10^-3^ | 2.3×10^-2^ | 6.8×10^-1^ |
| Occipital cortical gray matter | COPPercentInkTime_s | -9.7×10^-3^ | 2.3×10^-2^ | 6.8×10^-1^ |
| Occipital cortical gray matter | COMPercentInkTime_s | -9.5×10^-3^ | 2.3×10^-2^ | 6.8×10^-1^ |
| Occipital cortical gray matter | COMSpatialReasoning | 9.6×10^-3^ | 2.4×10^-2^ | 6.9×10^-1^ |
| Occipital cortical gray matter | COMPercentThinkTime_s | 9.0×10^-3^ | 2.3×10^-2^ | 7.0×10^-1^ |
| Occipital cortical gray matter | COMTotalTime_s | 8.7×10^-3^ | 2.3×10^-2^ | 7.1×10^-1^ |
| Occipital cortical gray matter | COPPercentInkTime | 8.5×10^-3^ | 2.3×10^-2^ | 7.2×10^-1^ |
| Occipital cortical gray matter | COPInkLength_s | -8.0×10^-3^ | 2.3×10^-2^ | 7.2×10^-1^ |
| Occipital cortical gray matter | COPAverageLatency_s | 8.0×10^-3^ | 2.3×10^-2^ | 7.3×10^-1^ |
| Occipital cortical gray matter | COPLongLatencyCount_s | 7.8×10^-3^ | 2.4×10^-2^ | 7.4×10^-1^ |
| Occipital cortical gray matter | COMSpatialReasoning_s | 7.6×10^-3^ | 2.4×10^-2^ | 7.5×10^-1^ |
| Occipital cortical gray matter | COPComponentPlacement | -7.3×10^-3^ | 2.3×10^-2^ | 7.6×10^-1^ |
| Occipital cortical gray matter | COMComponentPlacement_s | 6.9×10^-3^ | 2.4×10^-2^ | 7.7×10^-1^ |
| Occipital cortical gray matter | COMVerticalSpatialPlacement | 6.5×10^-3^ | 2.3×10^-2^ | 7.7×10^-1^ |
| Occipital cortical gray matter | COPAverageLatency | -6.8×10^-3^ | 2.4×10^-2^ | 7.7×10^-1^ |
| Occipital cortical gray matter | COPInformationProcessing_s | 6.5×10^-3^ | 2.3×10^-2^ | 7.8×10^-1^ |
| Occipital cortical gray matter | COPStrokeCountConformity_s | -6.5×10^-3^ | 2.4×10^-2^ | 7.8×10^-1^ |
| Occipital cortical gray matter | COMDrawingEfficiency | -6.3×10^-3^ | 2.4×10^-2^ | 7.9×10^-1^ |
| Occipital cortical gray matter | COMDrawingProcessEfficiency_s | 6.0×10^-3^ | 2.3×10^-2^ | 8.0×10^-1^ |
| Occipital cortical gray matter | DCTScore | 6.2×10^-3^ | 2.5×10^-2^ | 8.0×10^-1^ |
| Occipital cortical gray matter | COPStrokeCountConformity | -6.1×10^-3^ | 2.5×10^-2^ | 8.1×10^-1^ |
| Occipital cortical gray matter | COMOscillatoryMotion_s | 5.8×10^-3^ | 2.4×10^-2^ | 8.1×10^-1^ |
| Occipital cortical gray matter | COMPercentThinkTime | -5.5×10^-3^ | 2.3×10^-2^ | 8.1×10^-1^ |
| Occipital cortical gray matter | COPOscillatoryMotion_s | 5.4×10^-3^ | 2.3×10^-2^ | 8.1×10^-1^ |
| Occipital cortical gray matter | COMPercentInkTime | 5.2×10^-3^ | 2.3×10^-2^ | 8.2×10^-1^ |
| Occipital cortical gray matter | COPLongestLatency_s | 5.1×10^-3^ | 2.3×10^-2^ | 8.2×10^-1^ |
| Occipital cortical gray matter | COPLatencyVariability_s | 4.7×10^-3^ | 2.3×10^-2^ | 8.4×10^-1^ |
| Occipital cortical gray matter | COPDrawingProcessEfficiency | -4.5×10^-3^ | 2.4×10^-2^ | 8.5×10^-1^ |
| Occipital cortical gray matter | COPRelativeLongLatency_s | 4.1×10^-3^ | 2.3×10^-2^ | 8.6×10^-1^ |
| Occipital cortical gray matter | COPLatencyVariability | -4.1×10^-3^ | 2.4×10^-2^ | 8.6×10^-1^ |
| Occipital cortical gray matter | COPLongestLatency | -3.3×10^-3^ | 2.3×10^-2^ | 8.9×10^-1^ |
| Occipital cortical gray matter | COPPercentThinkTime | -3.3×10^-3^ | 2.3×10^-2^ | 8.9×10^-1^ |
| Occipital cortical gray matter | COPLongLatencyCount | 2.5×10^-3^ | 2.4×10^-2^ | 9.2×10^-1^ |
| Occipital cortical gray matter | COMInkLength | -2.0×10^-3^ | 2.3×10^-2^ | 9.3×10^-1^ |
| Occipital cortical gray matter | COPRelativeLongLatency | -1.7×10^-3^ | 2.4×10^-2^ | 9.4×10^-1^ |
| Occipital cortical gray matter | COMInkLength_s | -1.5×10^-3^ | 2.3×10^-2^ | 9.5×10^-1^ |
| Occipital cortical gray matter | COPTotalTime_s | -1.1×10^-3^ | 2.3×10^-2^ | 9.6×10^-1^ |
| Occipital cortical gray matter | COPComponentPlacement_s | 6.8×10^-4^ | 2.3×10^-2^ | 9.8×10^-1^ |
| Occipital cortical gray matter | COMVerticalSpatialPlacement_s | -3.9×10^-4^ | 2.3×10^-2^ | 9.9×10^-1^ |
| Occipital cortical gray matter | COPTotalTime | -4.0×10^-4^ | 2.4×10^-2^ | 9.9×10^-1^ |

The association of individual dCDT features with MRI measures was tested by linear regression models and adjusted for age, sex, and education.

^a^ All MRI measures were corrected for head size by calculating the percent of these volumes over the total cerebral cranial volume (TCV) above the tentorium. The percent of the white matter hyperintensity (WMH) volume/TCV was log-transformed.

^b^ Bonferroni correction was used to adjust for multiple testing, and significant dCDT features for each MRI variable were claimed if *P*<.05/105=4.76×10^-4^, where 105 was the number of tests performed. Significant *P* were indicated in **bold**.
